# L-Type Voltage-Gated Ca^2+^ Channels Are Targeted by Terpenes from *Hyptis crenata* Essential Oil in Vascular Electromechanical Coupling

**DOI:** 10.3390/medsci14020262

**Published:** 2026-05-20

**Authors:** André Nogueira Cardeal dos Santos, José Ednésio da Cruz Freire, Francisco Sydney Henrique Félix, Marília Cavalcante Araújo, Savyo Mikael Lacerda Gomes, Alexandre Lucas Lima França Cabral, Amanda Batista Nascimento, Cleisla Costa Barbosa, Marcus Vinícius Vieira Torquato, Lívia de Souza Oliveira, Luiz Henrique Batista Assunção, Sofia Moura de Sousa Brasil, Cecília Bessa Freitas, Julianne Ferreira da Silva, João Henrique Andrade de Menezes, Átila Pereira-Gonçalves, José Henrique Leal-Cardoso, Adélia Justina Aguiar Aquino, Andrelina Noronha Coelho de Sousa

**Affiliations:** 1 Experimental Physiology Laboratory, Superior Institute of Biomedical Sciences, State University of Ceará, Fortaleza 60714-903, CE, Brazil; sidhenriquee.08@gmail.com (F.S.H.F.); mariliaraujo@outlook.com (M.C.A.); savyolacerda@gmail.com (S.M.L.G.); prof.alexandrelucasflfc@gmail.com (A.L.L.F.C.); amanda.nascimento@aluno.uece.br (A.B.N.); cleisla.barbosa@aluno.uece.br (C.C.B.); vinicius.torquato@aluno.uece.br (M.V.V.T.); liviasouza.oliveira@aluno.uece.br (L.d.S.O.); henrique.batista@aluno.uece.br (L.H.B.A.); sofia.brasil@aluno.uece.br (S.M.d.S.B.); ceciliabessa31@gmail.com (C.B.F.); julianne.silva@aluno.uece.br (J.F.d.S.); joaohenriquejhadm@gmail.com (J.H.A.d.M.); atila.goncalves@uece.br (Á.P.-G.); 2 Department of Biomedicine and Pharmacy, Fametro University Center, Fortaleza 60010-470, CE, Brazil; 3 Biochemistry and Gene Expression Laboratory, Superior Institute of Biomedical Sciences, State University of Ceará, Fortaleza 60714-903, CE, Brazil; jednesio@gmail.com; 4 Faculty of Education and Integrated Sciences of Crateús, FAEC, State University of Ceará, Fortaleza 63704-155, CE, Brazil; 5 Electrophysiology Laboratory, Superior Institute of Biomedical Sciences, State University of Ceará, Fortaleza 60714-903, CE, Brazil; lealcard@gmail.com; 6 Department of Mechanical Engineering and Aerospace, Texas Tech University, Lubbock, TX 79409, USA; adelia.aquino@ttu.edu

**Keywords:** voltage-gated calcium channels, vascular smooth muscle contraction, synergistic interaction

## Abstract

Background: Electromechanical coupling is a fundamental process in the regulation of vascular smooth muscle contraction. It is characterized by changes in electrical potential membrane (depolarization). Voltage-gated calcium channels (VGCCs) play a central role in this process by mediating calcium influx necessary for vascular contraction. As highly conserved macromolecules in mammals, VGCCs represent translationally relevant targets for the development of vasorelaxant agents. Inhibition of these channels reduces calcium influx and attenuates the tonic smooth muscle contraction, making them strategic targets for novel therapeutic approaches. This is particularly important given the high prevalence of cardiovascular diseases, which remain the leading cause of global mortality. Methods: The aim of this study is to investigate the mechanism of action of terpenes in VGCCs. Terpenes are phytochemicals that have been widely studied as drug candidates. To this end, the oil from *Hyptis crenata* was extracted and characterized, revealing monoterpenes and sesquiterpenes as its main constituents. Results: In vitro assays on isolated aortic rings, with and without endothelium, demonstrated that these compounds reverse and block KCl (80 mM)-induced contractions in an endothelium-independent manner. Conclusions: Analyses of the ionic influx of calcium and barium indicated a progressive blockade of contraction, reinforcing the hypothesis of a direct interaction with the macromolecules of the VGCCs. Computational analyses, for the first time, suggest a potential synergistic interaction among terpenes in their binding to these macromolecules.

## 1. Introduction

**Figure 1:**
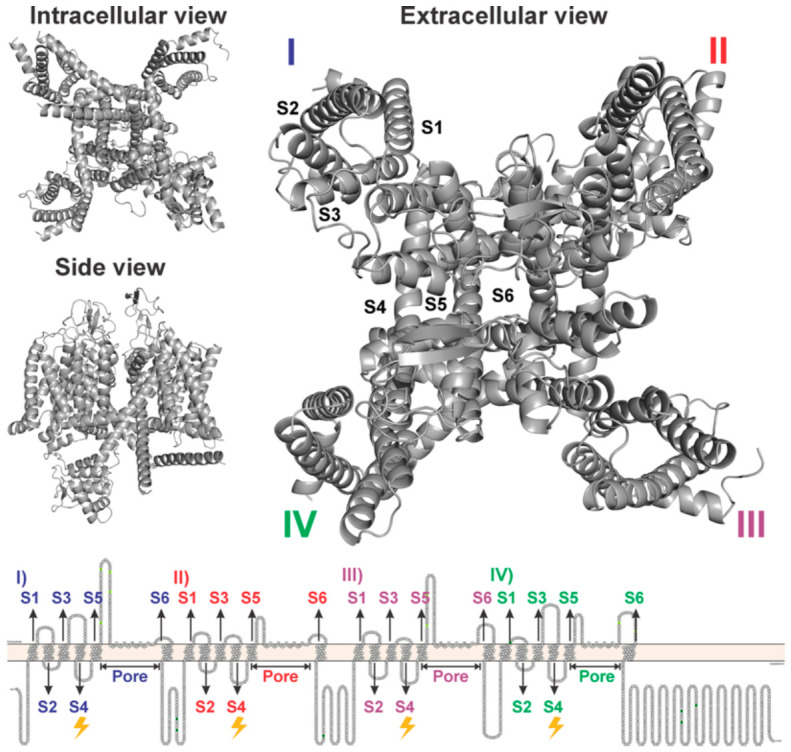
Representations of the VGCCs alpha-subunit structure from different angles. Includes views from the intracellular side, extracellular side, and a side view, along with a schematic diagram of the S1–S6 organization in the four domains (I–IV).

Vascular smooth muscle cells (VSMCs) regulate arterial tone through a tightly orchestrated system of signaling pathways that transduce extracellular cues into contractile responses [1]. Among these, the electromechanical coupling mechanism, initiated by membrane depolarization and mediated by voltage-gated calcium channels (VGCCs), plays a central role in the dynamic control of vessel diameter and tissue perfusion (see Figure 1) [2,3,4].

The influx of Ca^2+^ through L-type VGCCs is a primary trigger for actomyosin interaction [5], linking membrane excitability to mechanical force generation [6,7]. Due to their pivotal role in contractile activation, VGCCs have been extensively studied at the structural, functional, and pharmacological levels [8,9]. In addition to the pore-forming α_1_ subunit, voltage-gated calcium channels are composed of auxiliary subunits, including α_2_δ, β, and, in some isoforms, γ subunits. These auxiliary components play essential roles in channel trafficking to the plasma membrane, the stabilization of channel expression, and modulation of biophysical properties such as voltage dependence, kinetics, and current amplitude. In particular, the β subunit regulates channel gating and surface expression, while the α_2_δ subunit is critically involved in membrane targeting and pharmacological sensitivity, highlighting their importance in the functional regulation of VGCCs [8,9]. The α_1_-subunit, highly conserved across vertebrates, harbors critical domains for voltage sensing, pore formation, and ligand recognition [10]. These conserved features support their translational relevance and explain why VGCCs remain prominent molecular targets in the modulation of vascular reactivity. Small molecules capable of modulating VGCC activity can produce immediate and reversible changes in vascular tone, underscoring their therapeutic potential.

Natural products have emerged as valuable sources of ion channel modulators due to their structural diversity, bioactivity, and long-standing use in traditional medicine [11,12,13]. Essential oils (EOs), in particular, are complex mixtures of volatile secondary metabolites produced by aromatic plants. Many EOs exhibit pronounced effects on excitable and contractile tissues, and their biological activity is largely attributed to terpenoids, especially mono- and sesquiterpenes, which are the most abundant and pharmacologically active constituents [9,10,14,15]. Their small size, lipophilicity, and conformational flexibility favor interaction with membrane proteins, including calcium channels involved in electromechanical coupling. The essential oil of *Hyptis crenata* (EOHc), a South American aromatic species widely used in Brazilian folk medicine for inflammatory and gastrointestinal conditions [16,17], is particularly rich in terpenes such as camphor, β-caryophyllene, 1,8-cineole, β-caryophyllene oxide, and aromadendrene [18,19,20]. Other research demonstrates that the oil provides anti-edematogenic, anxiolytic, and hepatoprotective effects [21,22,23]. Previous studies have shown that EOHc induces smooth muscle relaxation, suggesting an effect on calcium-dependent contractile mechanisms. However, the precise molecular targets and signaling pathways involved in vascular smooth muscle remain poorly defined, and the interaction between its constituent terpenes and VGCCs has not been directly addressed. Importantly, the terpenes identified in EOHc are not unique to *H. crenata*, but are widely distributed among essential oils from various medicinal plants [24,25,26,27]. This ubiquity reinforces their broader pharmacological relevance and suggests that findings obtained from *H. crenata* may have generalizable implications for ion channel modulation.

In this study, we investigated the effect of EOHc on the electromechanical coupling pathway in rat aortic rings, both in the presence and absence of functional endothelium. Contractile responses were elicited by high-potassium depolarization (KCl, 80 mM), and cumulative concentrations of EOHc were applied to evaluate concentration-dependent relaxation. To further explore ion influx pathways, additional assays involving calcium and barium-induced contractions were performed. To help investigate these mechanisms, molecular simulation techniques, particularly molecular docking, have become indispensable tools. Docking calculations allow for the prediction of ligand–receptor interactions at the atomic level, enabling researchers to estimate binding affinities and identify key residues involved in molecular recognition [28,29]. To complement the functional data and explore potential molecular interactions, we also performed docking simulations targeting the α_1_-subunit of L-type VGCCs, aiming to identify putative binding sites for the major terpenes in EOHc.

## 2. Materials and Methods

### 2.1. Extraction and EOHc Characterization

This study was carried out using essential oil extracted from the dried leaves of *Hyptis crenata*, collected in the municipality of São Raimundo das Mangabeiras, in the state of Maranhão, Brazil. The essential oil extraction was carried out at the Superior Institute of Biomedical Sciences of the State University of Ceará (ISCB/UECE) through steam distillation. At the Technological Development Park (PADETEC) of the Federal University of Ceará (UFC), Fortaleza, Ceará, Brazil; the chemical composition of EOHc was analyzed, then an Agilent 7890B gas chromatograph coupled to a 5977A mass selective detector (quadrupole) equipped with a flame ionization detector was used to perform gas chromatography-mass spectrometry (GC-MS). The system was manufactured by Agilent Technologies, Santa Clara, CA, USA. Separation was conducted with an HP-5 MS methylpolysiloxane column (30 m × 0.25 mm × 0.25 μm; Agilent Technologies, Santa Clara, CA, USA). The injector, detector, and transfer line temperatures were set to 250 °C, 150 °C, and 280 °C, respectively. The oven temperature was initially set at 70 °C, increased at 4 °C/min to 180 °C, and subsequently ramped at 10 °C/min to 250 °C, resulting in a total run time of 34.5 min. Using MassHunter B.06.00 software (Agilent Technologies), mass spectra were recorded in the 40–600 *m*/*z* range. Compound identification was accomplished through a comparison of the acquired spectra with reference spectra from the National Institute of Standards and Technology (NIST) library.

### 2.2. Solutions

The Krebs–Henseleit solution (KHS) used had the following composition (in mM): 118.0 NaCl, 25 NaHCO_3_, 4.7 KCl, 1.2 KH_2_PO_4_, 1.2 MgSO_4_, 2.5 CaCl_2_, 0.026 EDTA, and 11 glucose, maintained at 37 °C with the pH adjusted to 7.4 using 1 M HCl and/or 1 M NaOH. The EOHc was prepared by diluting stock solutions directly in distilled water with 0.1% Tween. The calcium-free (ØCa^2+^) solution was prepared by omitting CaCl_2_ from the Krebs–Henseleit solution and adding 0.2 mM ethylene glycol-bis(β-aminoethyl ether)-N,N,N′,N′-tetraacetic acid (EGTA). All salts and reagents, including nifedipine (NIFE), were of analytical grade and obtained from Sigma-Aldrich (St. Louis, MI, USA).

### 2.3. Animals

Male Wistar rats (*Rattus norvegicus*) with weights between 200 and 300 g were obtained from the Christus University Center (UNICHRISTUS) and housed at the ISCB/UECE. The animals were maintained under controlled humidity and a temperature of 23 ± 2 °C, with a 12-h light/dark cycle and ad libitum access to water and food. All procedures followed the guidelines of the Brazilian College of Animal Experimentation (COBEA). The animal study protocol was approved by the Institutional Ethics Committee of the State University of Ceará (protocol code 31032.001146/2023-23, approved on 17 August 2023).

### 2.4. Preparation of Tissue

The animals were euthanized, followed by dissection of the thoracic aorta, which was sectioned into segments measuring 4–5 mm in length. The segments were maintained in a 5 mL chamber containing KHS and aerated with a carbogenic mixture (95% O_2_ and 5% CO_2_), at a temperature of 37 °C and pH 7.4. The contractile activity of the tissue was measured using a force transducer (Grass Technologies, Model FT03, West Warwick, RI, USA), with one end of the preparation connected to the transducer and the other to a fixed base via a stainless-steel rod. The transducer was coupled to a preamplifier (Model PM-100, DATAQ Instruments, Akron, OH, USA), which was connected to a data acquisition system (PowerLab 4/35, ADInstruments, Sydney, Australia), in turn linked to a computer. Data were visualized using LabChart 8.0 Pro software (ADInstruments). Data collected were stored in files using LabChart software (ADInstruments, Inc., Colorado Springs, CO, USA). Isolated aortic rings were subjected to a resting tension of 1 g and allowed to stabilize for 1 h. All protocols were initiated with a contraction induced by the addition of 80 mM KCl (K80) to the aortic rings. Upon reaching a stable plateau, the amplitude of the response was established as a reference value. Only those experiments that exhibited reproducible contractions of ≥1.5 gF were considered for experimental analysis. All experiments included a control preparation subjected to the same experimental protocols but without the essential oil concentrations. Endothelial integrity was accessed by initially contracting the tissue with 1 μM norepinephrine (NE), and after reaching a stable plateau, 10 μM of acetylcholine (ACh) was added to induce relaxation. Endothelial integrity was confirmed when the tissue exhibited a relaxation greater than 75%.

### 2.5. Electromechanical Coupling Reversal Protocol

Aortic rings were pre-contracted with K^+^ 80 mM to induce contraction through electromechanical coupling, following assessment of endothelial viability and integrity. Once the contraction plateaued, cumulative concentrations of EOHc (10, 30, 60, 100, 300, and 600 µg/mL) were added to the aortic rings with and without endothelium. Similarly, the vehicle control (Tween) was added to aortic rings under the same conditions.

### 2.6. Electromechanical Coupling Blocking Protocol

After assessing endothelial viability and integrity, the aortic rings were initially contracted with K^+^ 80 mM to induce contraction through electromechanical coupling. This contraction was established as a reference for subsequent K80-induced contractions, which were elicited after prior immersion in non-cumulative concentrations (10, 30, 60, 100, 300, 600 and 1000 µg/mL) of EOHc.

### 2.7. Calcium and Barium Ionic Curves Protocol

Once tissue viability had been assessed, the aortas were immersed in a Ca^2+^-free medium containing EGTA. K^+^ 80 mM was added to the extracellular solution. Increasing cumulative concentrations of CaCl_2_ (0.1, 0.3, 1, 3, 10, and 30 mM) were administered to induce contraction. After washing the tissue with a Ca^2+^-free solution, followed by the addition of K80, the preparations were incubated with different concentrations of EOHc (10, 100, 300, and 600 µg/mL). Tween was added in a similar manner at a single concentration of 600 µg/mL. The ability of CaCl_2_ (0.1, 0.3, 1, 3, 10, and 30 mM) to induce contraction was then reassessed. Additionally, the effects of NIFE (10^−8^, 10^−7^, and 10^−6^ µM), diluted in 0.05% DMSO, on BaCl_2_-induced contractions were evaluated using the same experimental protocol.

### 2.8. Statistical Analyses

The experimental data are expressed as the mean ± S.E.M. Statistical analysis and graph generations were performed using GraphPad Prism 8.0.2. Results were considered statistically significant when the null hypothesis probability was less than 5% (*p* < 0.05). The Student’s *t*-test and one- or two-way analysis of variance (ANOVA) were used, followed by Bonferroni’s and/or Tukey’s *t*-test and the Holm–Sidak multiple comparison method, when appropriate. Logarithmic interpolation was performed for calculations in each experiment.

### 2.9. Molecular Structure Analysis

We utilized the α1S subunit model of the L-type voltage-dependent calcium channel from *Rattus norvegicus*, as described in the literature [8]. Initially, the amino acid sequence of the L-type voltage-dependent calcium channel α1S (ID: rno:682930) from *R. norvegicus* was retrieved in FASTA format from the Kyoto Encyclopedia of Genes and Genomes (KEGG) database [30,31]. The native Ca^2+^ channel sequence of *Oryctolagus cuniculus* (NCBI Reference Sequence: NP_001095190.1) served as the template sequence. A structural model of the α1S subunit from *R. norvegicus* was predicted using the Swiss-Model server [32], employing the crystallographic structure of the Ca^2+^ channel from *O. cuniculus* (PDB ID: 5GJV) as a template. The initial model underwent structural refinement using the WinCoot software v0.9.8.92 (https://bernhardcl.github.io/coot/wincoot-download.html accessed on 16 April 2025) and was subsequently subjected to energy minimization via the YASARA server [33,34]. The final optimized model was validated through root mean square deviation (RMSD) calculations using the PyMOL Molecular Graphics System (version 1.7.4; Schrödinger LLC, New York, NY, USA). The physicochemical properties of the structure were assessed using the MolProbity server (http://molprobity.biochem.duke.edu/ accessed on 16 April 2025), while its overall structural quality was evaluated using the QMEAN [35] and VoroMQA [36] servers. Although CaV1.2 is the predominant L-type calcium channel expressed in vascular smooth muscle, the CaV1.1 structure was selected as a template due to its early availability and extensive structural validation. CaV1.1 and CaV1.2 share approximately 80% amino acid identity, with highly conserved transmembrane segments and ligand-binding regions. These conserved features support the use of CaV1.1 as a reliable surrogate model for predicting interactions relevant to CaV1.2. Furthermore, the recently resolved cryo-EM structure of human CaV1.2 corroborates the structural conservation among L-type calcium channels and reinforces the translational relevance of the present findings [37].

### 2.10. Molecular Docking Studies

We utilized AutoDock Tools v. 1.5.6 [38] and AutoDock Vina v. 1.2.0 [28,29,39] to obtain more detailed structural results from our computations. Initially, we conducted simulations of site-directed redocking, with all torsional bonds of the ligands allowed to rotate freely while maintaining the host receptors in a rigid state. This validation process was applied to the following models for the L-type VGCC subunit α1S: the crystal structure (PDB ID: 6JP5), co-crystallized with the ligand nifedipine, and a newly generated model based on the *R. norvegicus* organism. All polar hydrogens were added to the L-type VGCC subunit α1S structure, and the targets were properly parameterized with Gasteiger charges. Similarly, the ligands camphor (CID: 2537), caryophyllene (CID: 5281515), caryophyllene oxide (CID: 1742210), cineole (CID: 2758), and aromadendrene (CID: 91354) were prepared and parameterized using the same charge model. All simulations were performed under the following conditions: the number of conformations was set to 50, the exhaustiveness to 33, and the seed to 2009. For the VGCC subunit α1S and the ligand system, the box dimensions were uniformly set at XYZ = 30 Å, with center coordinates of X= −10.839, Y= 7.562, and Z= −4.064.

### 2.11. Virtual Screening, Pharmacokinetics, and Toxicity Prediction

SMILES strings of the major OEHc compounds were obtained from PubChem and evaluated using multiple in silico platforms, including pKCSM [40], ADMETlab 2 [41], SwissADME [42], Molinspiration Cheminformatics [43], AI Drug Lab [44], CODD-PRED [45], PRED-hERGG [46], XenoSite [47], VNN-ADMET [48], ToxinPRED [49], and ADMETopt [50]. Physicochemical properties analyzed included molecular weight, topological polar surface area (TPSA), partition coefficient (LogP), distribution coefficient (LogD), aqueous solubility (LogS), volume of distribution at steady state (VDss), and hydrogen bond capacity. ADME predictions encompassed gastrointestinal absorption, Caco-2 permeability, glycoprotein P (P-gp) interaction, plasma protein binding, volume of distribution, blood–brain barrier (BBB) and central nervous system (CNS) permeability, metabolism via CYP450 enzymes, and total clearance. Toxicity models assessed mutagenicity, carcinogenicity, hepatotoxicity, human Ether-à-go-go-related gene (hERG) inhibition, and other systemic effects. Drug-likeness was evaluated using the Lipinski, Pfizer, GSK, Golden Triangle, and Veber rules.

## 3. Results

### 3.1. Chemical Composition

The EOHc yield obtained by distillation was 1.2%. Chromatographic analysis revealed 47 peaks, with 99.99% of the constituents identified. The major constituents were camphor (28.16%), followed by β-caryophyllene (16.81%), caryophyllene oxide (peak 36: 7.27%; peak 45: 1.83%; peak 47: 0.51%; total: 9.61%), eucalyptol/cineole (7.33%), and Alloaromadendrene (peak 26: 4.48%; peak 29: 0.94%; total 5,42%), as shown in Figures S1–S47 and Table S1. These major constituents can be classified into two groups: monoterpenes (camphor and 1,8-cineole) and sesquiterpenes (β-caryophyllene, β-caryophyllene oxide, and aromadendrene). Literature reports have highlighted their potential pharmacological effects, including anti-inflammatory, antioxidant, gastroprotective, hepatoprotective, neuroprotective, and dermatoprotective activity, as well as their ability to modulate smooth muscle relaxation.

### 3.2. Electromechanical Coupling Reversal and Blocking Protocol

**Figure 2:**
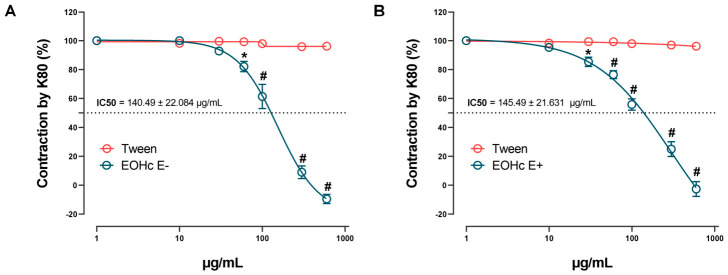
Effect of EOHc on 80 mM KCl-induced contraction in rat aortic rings. (**A**) Response in endothelium-denuded aorta (E−). (**B**) Response in endothelium-intact aorta (E+). Data points represent the mean ± standard error of the mean (SEM) from *n* = 6 experiments. The vehicle control (Tween) showed no significant effect on contraction. EOHc induced a concentration-dependent relaxation, with a significant effect starting at 30 μg/mL (E+) 60 μg/mL (E−) * *p* < 0.01, and becoming more pronounced at higher concentrations, # *p* < 0.0001, two-way ANOVA, Bonferroni’s test. The IC_50_ values were 140.49 ± 22.084 μg/mL for endothelium-denuded aorta and 145.49 ± 21.631 μg/mL for endothelium-intact aorta, with no statistically significant difference between the groups (one-way ANOVA, Tukey’s test).

The influence of EOHc on the steady-state (plateau) of K^+^-induced contractions was evaluated using increasing cumulative concentrations of the oil (10, 30, 60, 100, 300, and 600 μg/mL) in rat aortic preparations precontracted with 80 mM KCl. Experiments were conducted on both endothelium-intact and endothelium-denuded aortas (see Figure 2).

**Figure 3:**
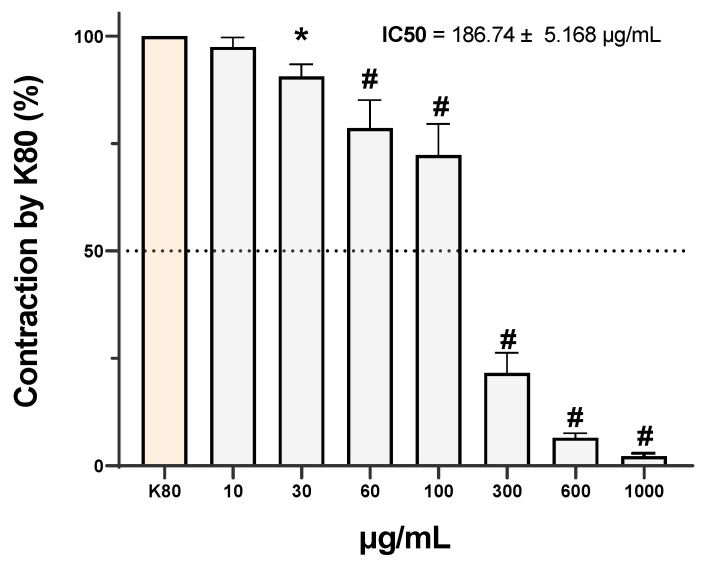
Effect of EOHc on 80 mM KCl-induced contraction in rat aortic rings. EOHc was applied at non-cumulative concentrations (10, 30, 60, 100, 300, 600, and 1000 μg/mL) before contraction induction. Data are expressed as mean ± standard error of the mean (SEM) from *n* = 4 experiments. EOHc induced a concentration-dependent inhibition, with a significant effect starting at 30 μg/mL (* *p* < 0.01) and becoming more pronounced at higher concentrations (# *p* < 0.0001, one-way ANOVA, Dunnett’s test). The IC_50_ value was 186.74 ± 5.168 μg/mL.

The results showed that EOHc induced concentration-dependent relaxation. Statistical analysis (two-way ANOVA followed by Bonferroni’s test) revealed significant differences at 60 μg/mL (*p* < 0.01) and at higher concentrations (*p* < 0.0001). Additional analysis determined IC_50_ values of 140.49 ± 22.084 μg/mL in endothelium-denuded aortas (Figure 2A) and 145.49 ± 21.631 μg/mL in endothelium-intact aortas (Figure 2B). No statistically significant difference was found between the IC_50_ values of the two groups (Student’s *t*-test, *p* > 0.05). Non-cumulative concentrations of the oil (10, 30, 60, 100, 300, 600, and 1000 μg/mL) in rat aortic preparations resulted in a concentration-dependent inhibition induced by EOHc (see Figure 3). Statistical analysis using one-way ANOVA followed by Dunnett’s test confirmed significant inhibition at concentrations ≥ 30 μg/mL (*p* < 0.01), with a more pronounced effect observed at higher concentrations (*p* < 0.0001) compared to the K80 control. Under this non-cumulative concentration protocol, EOHc demonstrated an IC_50_ value of 186.74 ± 5.168 μg/mL.

### 3.3. Evaluation of Voltage-Dependent Calcium Channel Involvement Using Calcium and Barium Ion Curves

**Figure 4:**
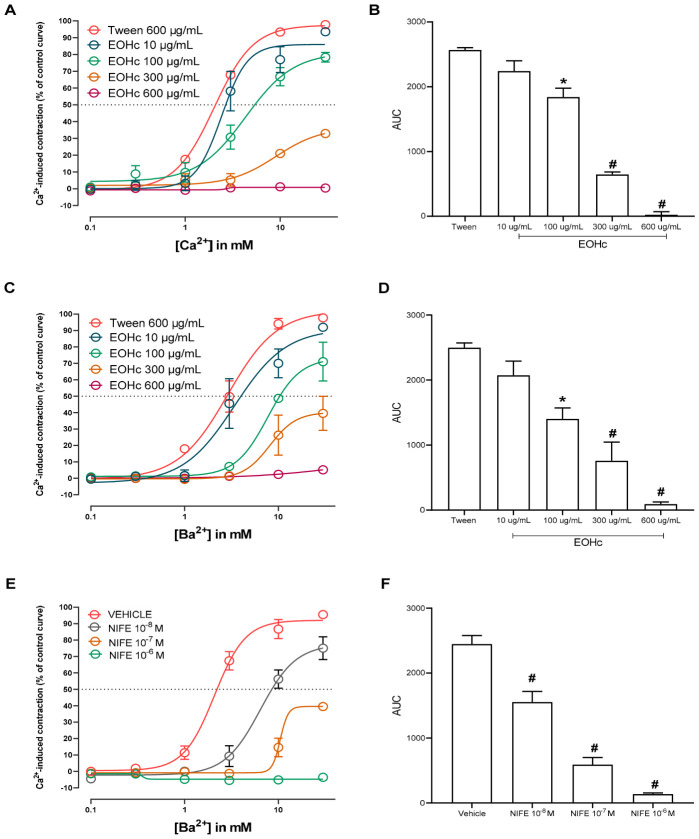
The effect of EOHc on Ca^2+^ and Ba^2+^-induced contractions in rat aortic rings maintained depolarized in a calcium-free medium was evaluated. (**A**) The contractile effect curve induced by increasing Ca^2+^ concentrations demonstrated a concentration-dependent response. (**B**) Analysis of the area under the curve revealed that EOHc at concentrations of 100 μg/mL and 300 μg/mL significantly reduced the Ca^2+^-induced contractile response, while 600 μg/mL resulted in complete blockade. (**C**) The contractile effect curve induced by increasing Ba^2+^ concentrations was also assessed. (**D**) The area under the curve analysis indicated that EOHc significantly inhibited Ba^2+^-induced contractions at concentrations ≥ 100 μg/mL (* *p* < 0.001 and # *p* < 0.0001). (**E**) The contractile effect curve induced by increasing Ba^2+^ concentrations in the presence of NIFE demonstrated a concentration-dependent inhibitory effect on vascular contraction. (**F**) Analysis of the area under the curve showed that NIFE significantly reduced the Ba^2+^-induced contractile response at concentrations of 10^−8^ μM, 10^−7^ μM and 10^−6^ μM, with near-complete inhibition observed at the highest concentration tested. Data are expressed as mean ± standard error of the mean (SEM) and were analyzed using one-way ANOVA followed by Tukey’s test.

The evaluation of the calcium response curve revealed that the cumulative addition of Ca^2+^ in a calcium-free medium following K80-induced depolarization produced concentration-dependent contractions, with a maximal response observed at 30 mM Ca^2+^ (Figure 4A). Administration of EOHc at 10 μg/mL did not produce a statistically significant difference from the control (Tween). However, concentrations of 100 μg/mL and 300 μg/mL significantly reduced the Ca^2+^-induced contractile response (*p* < 0.0001), as indicated by a shift of the curve to the right and a decrease in its amplitude, while 600 μg/mL completely abolished contraction. This rightward shift suggests a reduction in Ca^2+^ sensitivity in accord the requirement of a tenfold increase in extracellular Ca^2+^ concentration to achieve a 20% contraction level in the presence of 300 μg/mL EOHc compared to the control (600 μg/mL Tween). Analysis of the area under the curve confirmed this significant attenuation of contractile response at concentrations ≥ 100 μg/mL, thereby reinforcing the hypothesis of a concentration-dependent inhibitory effect (Figure 4B).

**Figure 5:**
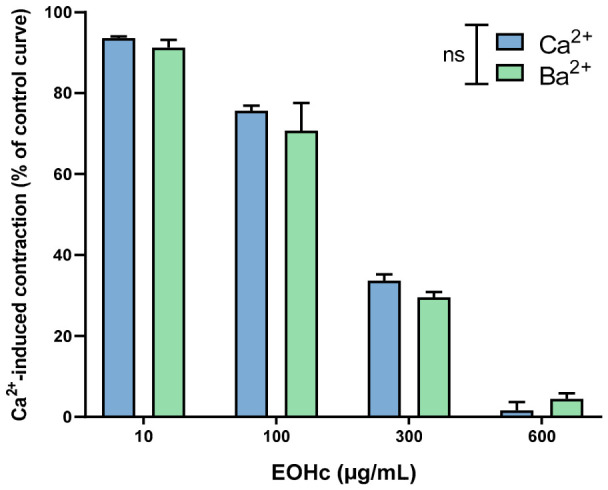
Comparison of the effects of EOHc on Ca^2+^- and Ba^2+^-induced contractions in depolarized rat aortic rings. Although EOHc significantly reduced the contractile responses induced by both ions, the comparison between the curves did not reveal any statistically significant differences at any concentration (one-way ANOVA followed by Tukey’s test).

To investigate whether EOHc activity was associated with the modulation of VGCCs, concentration–response curves were obtained using Ba^2+^. The data showed a similar pattern to that observed for Ca^2+^: EOHc significantly reduced Ba^2+^-induced contractions at 100 μg/mL (* *p* < 0.001) and 300 μg/mL (# *p* < 0.0001), and completely suppressed the contractile response at 600 μg/mL (Figure 4C,D). The area under the Ba^2+^ curve further confirmed a significant reduction at concentrations ≥ 100 μg/mL, indicating inhibition of VGCCs-mediated ion influx. To further confirm the involvement of VGCCs in the vasorelaxant effect of EOHc, concentration–response curves to Ba^2+^ were also obtained in the presence of NIFE, a selective L-type calcium channel blocker. NIFE significantly reduced Ba^2+^-induced contractions at 10^−7^ µM (# *p* < 0.0001) and completely abolished the contractile response at 10^−6^ µM (# *p* < 0.0001), while 10^−8^ µM produced a partial inhibitory effect (Figure 4E,F). The comparison between the Ca^2+^ and Ba^2+^ response curves revealed that although EOHc attenuated contractions induced by both ions, no statistically significant differences were observed between the areas under the curves of the two groups, regardless of the tested concentration (Figure 5).

### 3.4. Model Structure and Validation

**Figure 6:**
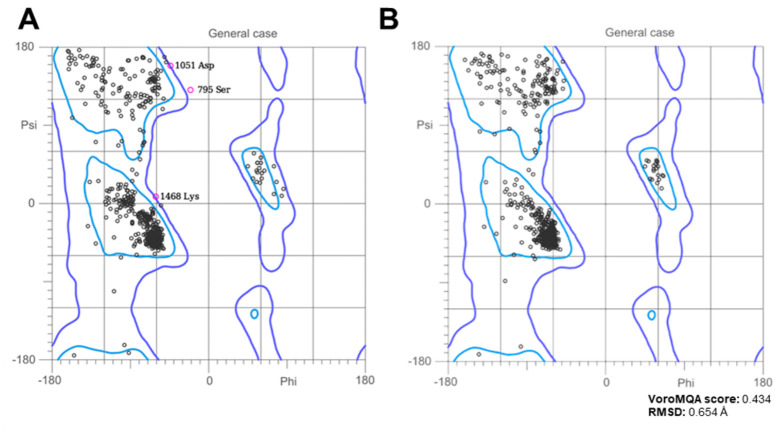
Ramachandran plots of the voltage-dependent L-type calcium channel model before (**A**) and after (**B**) energy minimization using YASARA. The black dots represent the distribution of backbone dihedral angles (Φ and Ψ) of the amino acid residues. After minimization, a greater number of residues are located within the allowed and favored regions, indicating an improvement in structural quality. The VoroMQA score (0.434) and RMSD (0.654 Å) were used to assess model refinement, suggesting moderate improvement while maintaining structural integrity.

To assess the structural quality of the modeled voltage-dependent L-type calcium channel, *Ramachandran* plots were generated before (Figure 6A) and after (Figure 6B) energy minimization using YASARA. Initially, a significant number of residues were found outside the allowed regions, indicating structural strain and potential inaccuracies in the model. Following energy minimization, a redistribution of residues into the favored regions was observed, suggesting an improvement in structural stability. Additionally, structural validation was performed using VoroMQA, VERIFY3D, QMEAN, and RMSD calculations. The VoroMQA score of 0.434 suggests a moderate structural quality, as values above 0.4 are typically associated with high-quality models. The RMSD of 0.654 Å, calculated using PyMOL, indicates that the structural refinement did not cause major perturbations while optimizing the model.

### 3.5. Site-Specific Molecular Docking Results and Interaction Mapping

Molecular docking calculations were performed using a search box of the entire pore region formed by the S5 and S6 segments and the adjacent areas including the voltage sensor located in the S4 segment. This configuration made it possible to assess both direct interactions within the pore and potential modulation of the voltage sensor mediated by the S4 segment. A total of 20 simulations were carried out resulting in multiple interaction configurations. A detailed analysis of all generated configurations resulted in five main interaction sites (Sites 1–5) within the α1S subunit (Table S2). The specific characteristics of these interaction sites are described and analyzed in detail in the following sections.

**Figure 7:**
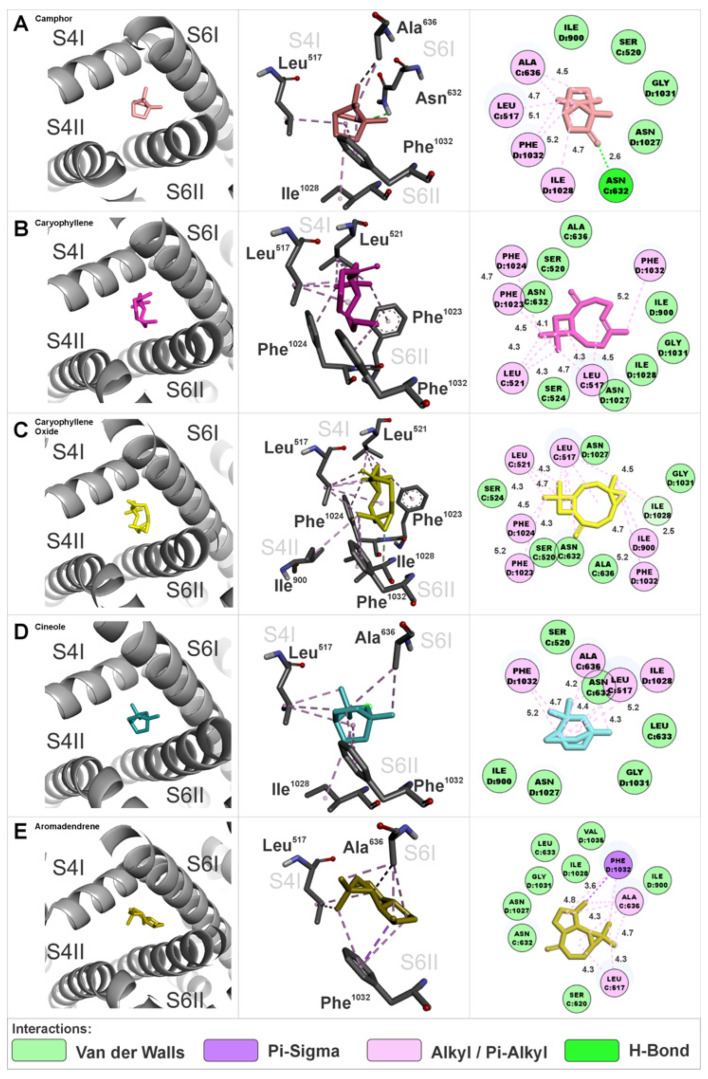
Interaction site 1 in the α1S subunit of the VGCCs, involving segments S4 and S6 of domain I, and S4 and S6 of domain II. Three-dimensional and two-dimensional representations illustrate the amino acids interacting with the EOHc constituents.

At interaction site 1, the EOHc constituents were anchored in a region defined by the S4 segment of domain I, the S6 segment of domain I, the S4 segment of domain II, and the S6 segment of domain II. The amino acids involved in these interactions are presented in three-dimensional and two-dimensional representations specific to each constituent (Figure 7A–E). The calculated Gibbs free energy variation (ΔG) values for the major constituents were as follows: (A) camphor (ΔG = −6.0 kcal.mol^−1^), (B) β-caryophyllene (ΔG = −7.9 kcal.mol^−1^), (C) β-caryophyllene oxide (ΔG = −8.0 kcal.mol^−1^), (D) cineole (ΔG = −6.2 kcal.mol^−1^), and (E) aromadendrene (ΔG = −7.5 kcal.mol^−1^).

**Figure 8:**
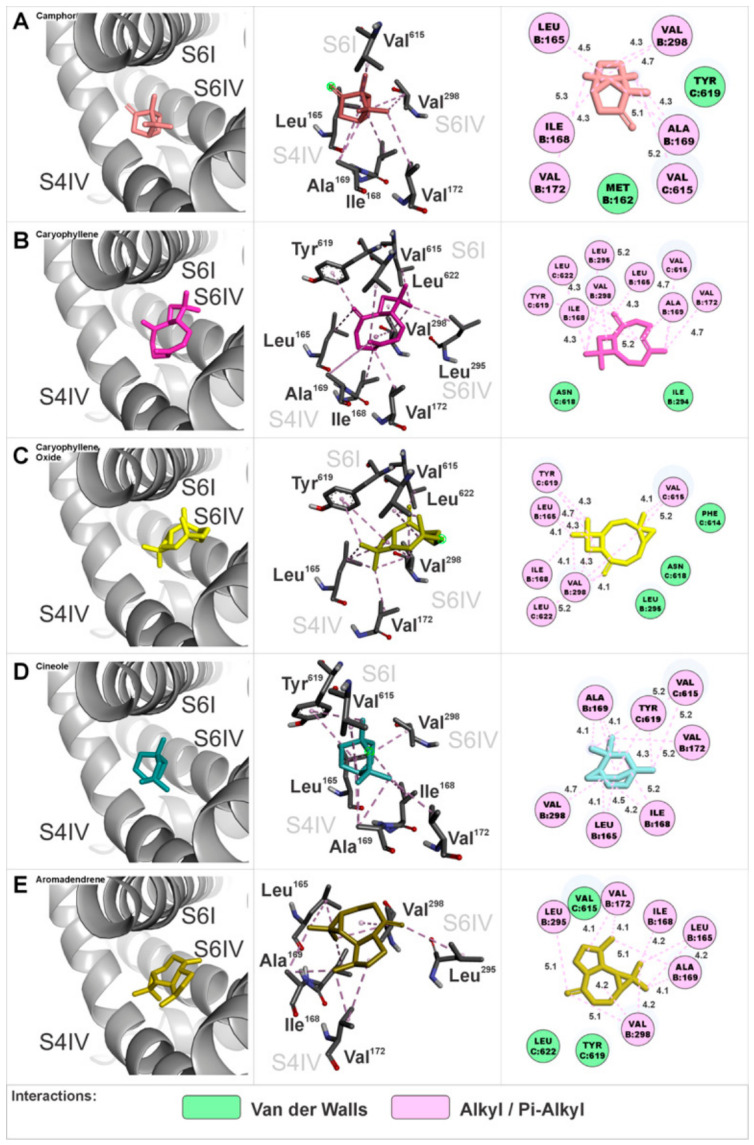
Interaction site 2 in the α1S subunit of the VGCCs, involving segments S6 of domain I and S4 and S6 of domain IV. Three-dimensional and two-dimensional representations illustrate the amino acids interacting with the EOHc constituents.

The EOHc constituents at interaction site 2 were anchored in a region composed of the S4 segment of domain IV, the S6 segment of domain I, and the S6 segment of domain IV. The amino acids involved in these interactions are presented in three-dimensional and two-dimensional representations specific to each constituent (Figure 8A–E). The calculated Gibbs free energy variation (ΔG) values for the major constituents were as follows: (A) camphor (ΔG = −5.7 kcal.mol^−1^), (B) β-caryophyllene (ΔG = −7.9 kcal.mol^−1^), (C) β-caryophyllene oxide (ΔG = −7.8 kcal.mol^−1^), (D) cineole (ΔG = −5.6 kcal.mol^−1^), and (E) aromadendrene (ΔG = −7.4 kcal.mol^−1^).

**Figure 9:**
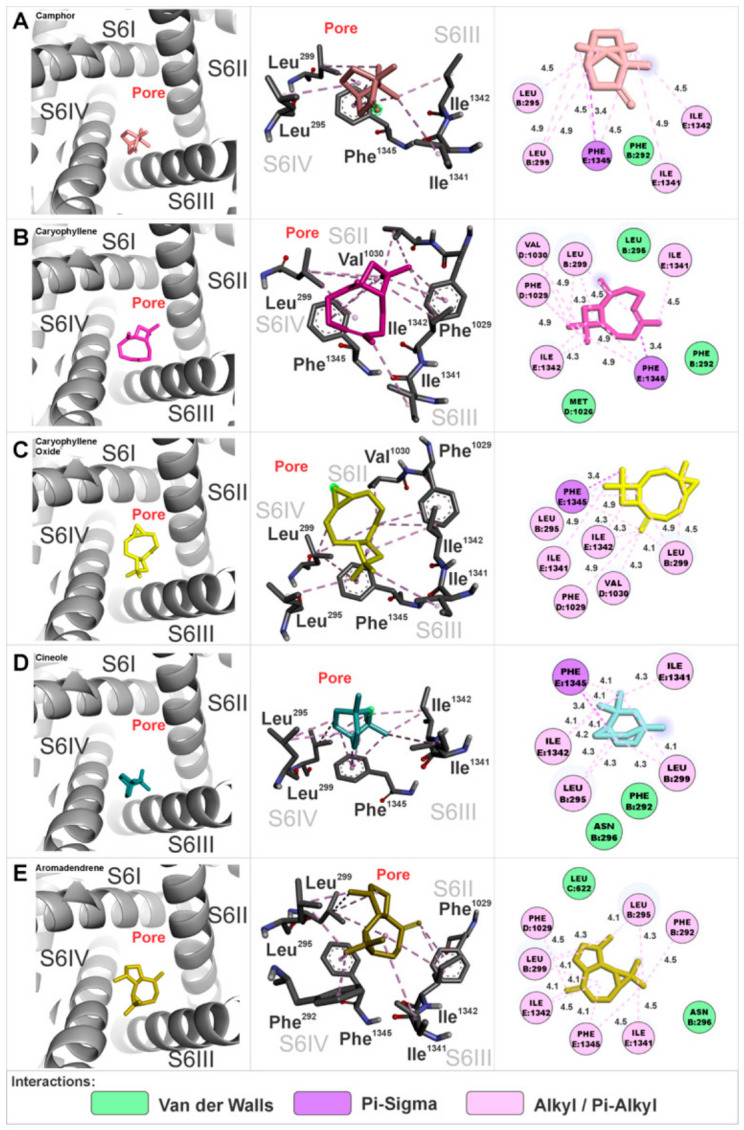
Interaction site 3 in the α1S subunit of the VGCCs, involving segments S6 of domain I, S6 of domain II, S6 of domain III, and S6 of domain IV, which form the channel pore. Three-dimensional and two-dimensional representations illustrate the amino acids interacting with the EOHc constituents.

Interaction site 3 is located in the pore-forming region of the channel, anchoring the constituent molecules of EOHc in an area composed of the S6 segments of domains I, II, III, and IV. The amino acids involved in these interactions are represented in detail in three-dimensional and two-dimensional figures specific to each constituent (Figure 9A–E).

Notably, this region serves as the binding site for calcium channel blockers belonging to the phenylalkylamine and benzothiazepine classes, represented by compounds such as verapamil and diltiazem, respectively. The calculated Gibbs free energy variation (ΔG) values for the major constituents were as follows: (A) camphor (ΔG = −5.6 kcal.mol^−1^), (B) β-caryophyllene (ΔG = −7.3 kcal.mol^−1^), (C) β-caryophyllene oxide (ΔG = −7.3 kcal.mol^−1^), (D) cineole (ΔG = −5.5 kcal.mol^−1^), and (E) aromadendrene (ΔG = −7.6 kcal.mol^−1^).

**Figure 10:**
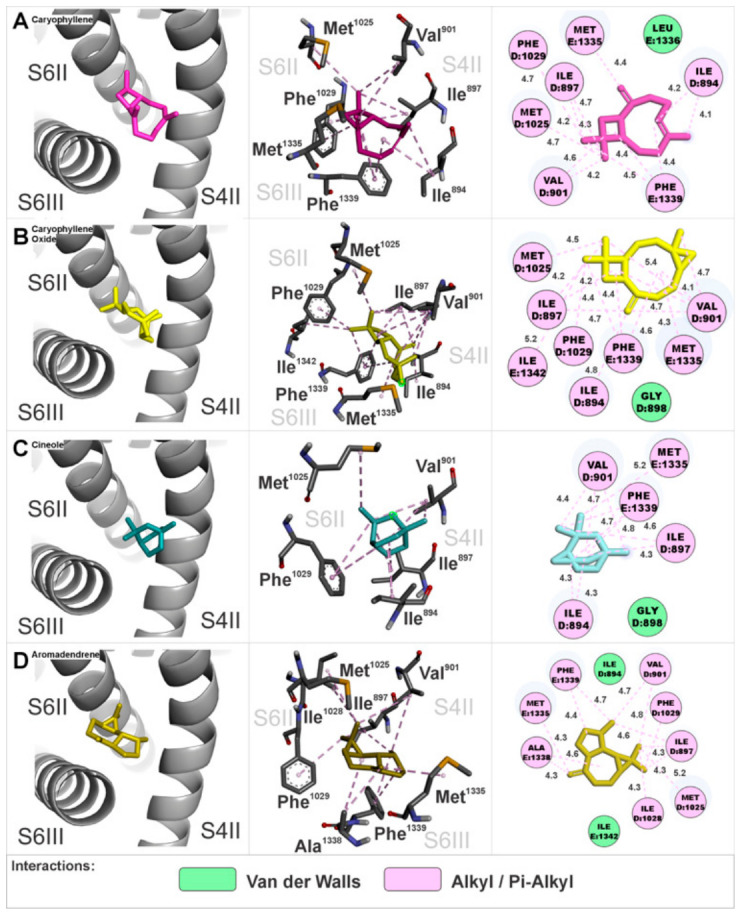
Interaction site 4 in the α1S subunit of the VGCCs, involving segments S4 of domain II, S6 of domain II, and S6 of domain III. Three-dimensional and two-dimensional representations illustrate the amino acids interacting with the EOHc constituents.

Anchoring of EOHc constituents at interaction site 4 was in a region composed of the S4 segment of domain II as well as the S6 segments of domains II and III. The amino acids involved in these interactions are represented in detail in three-dimensional and two-dimensional figures specific to each constituent (Figure 10A–D).

We noted that camphor did not exhibit anchoring at this site, unlike the other constituents. It is important to point out that this a region adjacent to the canonical dihydropyridine-binding pocket, such as NIFE and Bay K. The calculated Gibbs free energy variation (ΔG) values for the major constituents were as follows: (A) β-caryophyllene (ΔG = −7.4 kcal.mol^−1^), (B) β-caryophyllene oxide (ΔG = −7.2 kcal.mol^−1^), (C) cineole (ΔG = −5.3 kcal.mol^−1^), and (D) aromadendrene (ΔG = −7.3 kcal.mol^−1^).

**Figure 11:**
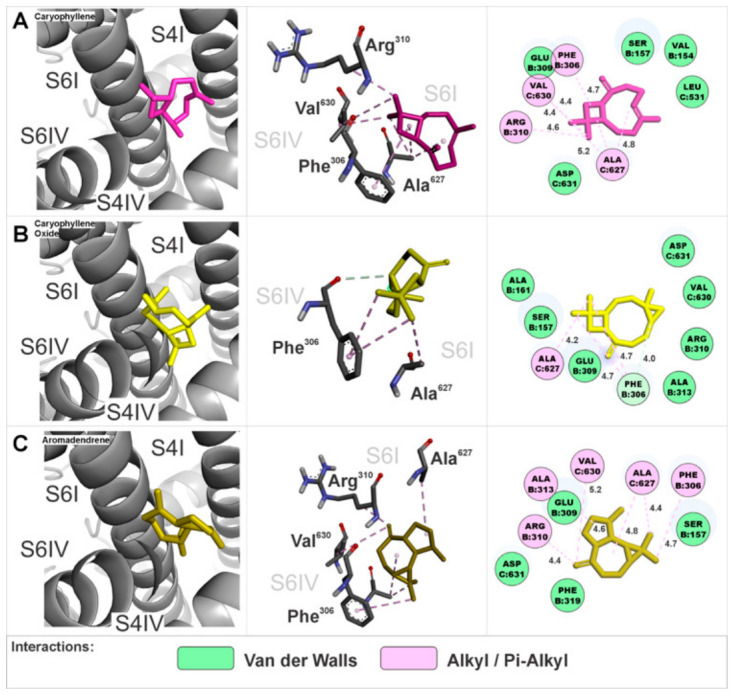
Interaction site 5 in the α1S subunit of the calcium channel, involving segments S4 of domain I, S4 of domain IV, S6 of domain I, and S6 of domain IV. Three-dimensional and two-dimensional representations illustrate the amino acids interacting with the EOHc constituents.

All of the interaction site 5 anchoring of EOHc constituents was found in a region composed of the S4 segments of domains I and IV as well as the S6 segments of domains I and IV. The amino acids involved in these interactions are detailed in three-dimensional and two-dimensional representations specific to each constituent (Figure 11A–C). The calculated Gibbs free energy variation (ΔG) values for the major compounds were: (A) β-caryophyllene (ΔG = −6.9 kcal.mol^−1^), (B) β-caryophyllene oxide (ΔG = −6.8 kcal.mol^−1^), and (C) aromadendrene (ΔG = −6.9 kcal.mol^−1^). The monoterpenoids did not exhibit anchoring at this site.

### 3.6. Molecular Bioprospecting

In silico predictions of the five major ligands revealed that molecular weight (MW), topological polar surface area (tPSA), and partition coefficient (LogP) conform to Lipinski’s Rule of Five, with no violations observed. However, only the major monoterpenes (camphor and cineole) met Pfizer’s rule (LogP < 3), whereas only the sesquiterpenes (caryophyllene, caryophyllene oxide, and aromadendrene) complied with the Golden Triangle rule (MW > 200 Da and LogP < 5). All compounds showed one hydrogen bond acceptor and no hydrogen bond donors. Quantitative estimate of drug-likeness (QED) scores ranged from 0.445 to 0.521, indicating moderate drug-likeness. The predicted gastrointestinal absorption (GIA) was high for most compounds, primarily driven by passive diffusion. Human intestinal absorption (HIA) was estimated at up to 95%. However, the Caco-2 cell permeability results suggest only moderate permeability. None of the compounds acted as inhibitors of P-glycoprotein (P-gp) classes I or II, though camphor was predicted to be a P-gp substrate. The volume of distribution at steady state (VDss) ranged from 2.14 to 5.67 L/kg, suggesting moderate to extensive tissue distribution. All compounds showed high blood–brain barrier (BBB) permeability, indicating potential CNS exposure. Total clearance varied between 0.04 and 0.762 mL/min/kg, with monoterpenes displaying higher clearance rates, implying faster elimination. None of the compounds interacted with the renal OCT2 transporter, suggesting low potential for renal drug–drug interactions. Aromadendrene and caryophyllene oxide were predicted to inhibit CYP2C19, CYP2C9, CYP2D6, and CYP3A4 enzymes but were unlikely to act as substrates. Camphor, cineole, and caryophyllene showed no relevant CYP450 interactions, indicating a lower risk for metabolic drug–drug interactions. Toxicity predictions indicated low mutagenic risk (AMES ≤ 0.025) and low potential for hepatotoxicity, DILI, nephrotoxicity, hematotoxicity, and cardiotoxicity (no hERG I/II inhibition). However, the high BBB permeability raises concern for potential neurotoxicity, warranting further investigation into CNS safety (Table S3).

## 4. Discussion

### 4.1. Electromechanical Coupling Reversal and Blocking Protocol

Electromechanical coupling in vascular smooth muscle relies on membrane depolarization to activate VGCCs [51], leading to increased intracellular Ca^2+^ levels and subsequent contractile responses via the calmodulin–MLCK pathway [52]. In this study, EOHc effectively reversed contractions induced by K80, displaying similar IC_50_ values in preparations with and without endothelium, indicating an endothelium-independent mechanism. Notably, under the pre-contracted (blockade) condition, the IC_50_ was slightly higher, suggesting increased sensitivity of depolarized cells to EOHc.

While KCl-induced depolarization primarily activates VGCCs, additional pathways, such as store-operated calcium channels (SOCCs), transient receptor potential (TRP) channels, and Rho-kinase signaling, may also contribute to the contractile response [53,54]. Thus, although EOHc reversed and attenuated K^+^-induced contractions, this effect alone is insufficient to attribute its mechanism exclusively to VGCC inhibition. To further dissect the specificity of EOHc’s action, we employed Ca^2+^ and Ba^2+^ cumulative concentration–response protocols under depolarizing conditions.

### 4.2. Evaluation of Voltage-Dependent Calcium Channel Involvement Using Calcium and Barium Ion Curves

This experiment aimed to elucidate the involvement of voltage-gated calcium channels (VGCCs) in vascular contraction and to investigate whether the mechanism of action of EOHc terpenes involves the direct blockade of these channels. In terms of membrane ion channels [55], the nearly identical inhibition of contractions induced by Ca^2+^ and Ba^2+^ suggests that the inhibitory effect is mediated specifically through the blockade of VGCCs [56]. This conclusion is supported by the fact that while Ca^2+^ can enter the cell through multiple pathways [57], Ba^2+^ is highly selective for VGCCs, as it does not readily permeate other calcium-permeable channels [58]. Furthermore, the hypothesis that this blockade occurs primarily through intracellular mechanisms is improbable. For such a hypothesis to be valid, it would be necessary to assume that the intracellular effects of Ba^2+^ on the contractile machinery are identical to those of Ca^2+^. Given that Ba^2+^ is a poor substitute for Ca^2+^ in activating downstream signaling pathways (such as the calmodulin-MLCK cascade), the equipotent inhibition observed indicates that EOHc acts as a direct modulator of VGCC-mediated influx, thereby interfering with electromechanical coupling in vascular smooth muscle [59,60,61].

The comparison between the Ca^2+^ and Ba^2+^ response curves revealed that although EOHc attenuated contractions induced by both ions, no statistically significant differences were observed between the areas under the curves of the two groups, regardless of the tested concentration. This finding indicates that the inhibitory effect of EOHc primarily occurs through the modulation of Ca^2+^ entry via VGCCs, with no result influence on downstream intracellular signaling pathways. Therefore, the present results support the hypothesis that EOHc acts as a direct modulator of VGCC-mediated Ca^2+^ influx, thereby affecting electromechanical coupling in vascular smooth muscle. Accordingly, in silico analyses using molecular docking were performed to elucidate and support the potential interactions of the molecules in this channel.

The use of NIFE as a pharmacological control was essential to confirm the functional presence of VGCCs in the rat aortic rings under the experimental conditions employed. Since Ba^2+^ is a well-established permeant ion capable of passing through L-type calcium channels and sustaining smooth muscle contraction; the marked inhibition of Ba^2+^-induced contractions by NIFE demonstrates that the contractile responses obtained in this model were probability mediated by L-type VGCC activation [59,60,61]. The progressive reduction in vascular contraction observed in the presence of NIFE, culminating in near-complete blockade at the highest concentration tested, validates the sensitivity and reliability of the experimental protocol for evaluating compounds that interfere with calcium influx through these channels. Thus, the similarity between the inhibitory profile produced by NIFE and that observed with EOHc strengthens our hypothesis that the vasorelaxant effect of the extract is, at least in part, associated with the blockade of L-type calcium channels in vascular smooth muscle.

### 4.3. Structural Consistency and Translational Potential of VGCCs

VGCCs are complex transmembrane proteins composed of five subunits: α_1_, α_2_, β, γ, and δ [62]. Among these, the α_1_ subunit is the primary component, as it contains the selective pore responsible for calcium ion influx into the intracellular environment. The remaining subunits play structural and regulatory roles, modulating channel activity. The α_1_ subunit consists of four distinct domains (I, II, III, and IV), each of which comprises six transmembrane segments (S1 to S6) [63]. The S4 segment plays a crucial role in voltage sensitivity, as it functions as a sensor for membrane potential variations. The S5 and S6 segments, on the other hand, along with interdomain regions, form the central pore of the channel, enabling the selective passage of calcium ions [63]. Experimental results indicated that the constituents present in EOHc act through an electromechanical mechanism, blocking and/or modulating voltage-dependent calcium channels. This hypothesis was investigated further by a molecular docking analysis, focusing on the α1S subunit of the L-type calcium channel.

It is important to highlight the translational potential of this biological target, given that the amino acid sequence of this channel is highly consistent among mammals with over 95% identity [10]. Furthermore, the interaction sites of dihydropyridines such as nifedipine, phenylalkylamines such as verapamil, and benzodiazepines such as diltiazem, are 100% consistent for these molecules [10,64]. The anchoring of the major terpenes present in EOHc to these sites therefore suggests a high likelihood of translational applicability across different mammalian species. The experimental findings support this hypothesis and demonstrate contraction reversal, indicating that the voltage-dependent calcium channel is the primary molecular target of these compounds. The effects observed in this study may then have translational relevance for other mammalian species. Previous studies have reported the involvement of terpenes in the modulation of this specific channel. Recent evidence has also suggested that cineole, one of the major monoterpenes, may act as a blocker of this target [9,14,65]. However, this hypothesis has not yet been thoroughly investigated. The present study provides a more detailed elucidation of this mechanism of action, not only for cineole but also for other EOHc constituents, and offers a stronger foundation for future investigations into their pharmacological potential.

### 4.4. Progressive and Synergistic Blockade of VGCCs Channels by EOHc Terpenes: A Molecular Docking Approach

Given that the experimental findings suggested the interaction of terpenes present in EOHc on VGCCs, a pharmacodynamics analysis was conducted using docking computations to further elucidate the molecular mechanisms underlying this interaction. Molecular docking analysis identified five preferential interaction sites, referred to as hotspots or interaction clusters. These clusters are located between segments S4 and S6 in each of the four domains (I, II, III, and IV). The distribution of these interactions may suggest a synergistic mechanism of action, in which small molecules, such as EOHc terpenes with a molecular weight below 300 Daltons, anchor at these sites in a concentration-dependent manner. As the EOHc concentration increases, a greater number of these clusters become occupied, leading to a progressive modulation of the channel. The interaction sites can be categorized into two main functional groups based on their effects on channel activity. Modulatory sites (clusters 1, 2, 4, and 5): The major constituents of EOHc bind laterally to the pore, forming interactions between segments S4 and S6 without completely obstructing calcium influx. Notably, cluster 4 corresponds to the binding site of dihydropyridines such as nifedipine [64]. This interaction suggests a modulatory role in the channel’s activation and inactivation kinetics. Blocking site (cluster 3): This site is particularly relevant, as interactions occur between the four S6 segments, which directly form the channel pore. The anchoring of molecules at this site may result in an effective blockade of calcium ion passage, thereby preventing smooth vascular muscle contraction. This region is also known to serve as the binding site for calcium channel blockers of the phenylalkylamine class such as verapamil, and the benzothiazepine class such as diltiazem [64].

**Figure 12:**
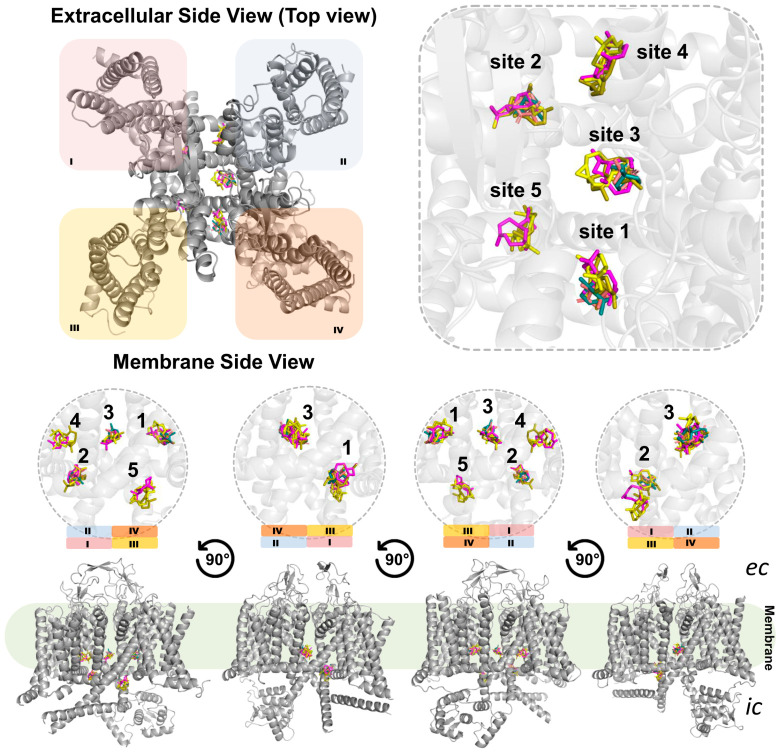
Structural mapping of ligand-binding sites in a membrane protein. Extracellular and membrane side views reveal the spatial distribution of five distinct binding sites (sites 1–5), with ligands shown in multiple colors representing different docking conformations. Enlarged panels highlight each site, demonstrating consistent clustering and overlap of ligand poses within defined cavities. Membrane side view obtained by successive 90° rotations illustrate the orientation of binding sites across the membrane, with the lipid bilayer indicated, separating the extracellular (ec) and intracellular (ic) regions. The protein is depicted in gray cartoon representation, while ligands are shown as colored sticks. The four homologous domains of the α_1_ subunit (domains I–IV) are indicated using color-coded regions to facilitate topological orientation and structural interpretation (domain I, purple; domain II, blue; domain III, yellow; domain IV, orange). Enlarged panels emphasize each binding site, demonstrating the overlap and preferential localization of ligands in specific regions of the channel.

A key experimental finding supporting the hypothesis of progressive, concentration-dependent VGCC blockade by EOHc is the rightward shift in the calcium response curve. When the extracellular calcium concentration is increased in the presence of 10 and 100 µg/mL of EOHc, the contraction percentage remains constant, which indicates a partial modulatory effect on channel activity. This effect may be attributed to the occupation of lateral clusters (sites 1, 2, 4, and 5) or even cluster 3, acting as a “sub-gate” at lower concentrations. As the extracellular calcium gradient increases, however, the molecules are progressively displaced from the pore interaction site. This displacement allows calcium ions to overcome the channel blockade, as reflected in the contraction percentages observed at 100 and 300 µg/mL. In contrast, a complete inhibition of calcium-induced contraction was observed at 600 µg/mL, suggesting that all interaction clusters, including site 3, were occupied by EOHc molecules, resulting in a full blockade of calcium influx (Figure 12). The vasorelaxant effect of the essential oil may result from cooperative interactions among its constituents. Molecular docking suggests that monoterpenes and sesquiterpenes can bind to distinct sites of L-type voltage-gated calcium channels, potentially producing additive or synergistic effects. However, these findings should be interpreted with caution, as they are based on computational predictions. Definitive confirmation of synergism requires quantitative analyses, such as isobolographic methods or combination index (CI) determinations, which should be addressed in future studies.

### 4.5. Potential Involvement of Different Calcium Channel Subtypes

Although L-type calcium channels (particularly CaV1.2) are the primary mediators of electromechanical coupling in vascular smooth muscle, low-threshold calcium channels (e.g., CaV3.x) are also expressed and may contribute to vascular tone under specific conditions. However, the experimental approaches employed in this study, including KCl-induced depolarization and Ca^2+^/Ba^2+^-induced contraction assays, predominantly assess high-voltage-activated channels, supporting the involvement of L-type calcium channels. Furthermore, given the high structural conservation among L-type isoforms, it is plausible that terpene compounds from *Hyptis crenata* essential oil may interact not only with CaV1.2 but also with CaV1.3. Nevertheless, further studies are required to evaluate isoform-specific interactions and the potential contribution of low-threshold calcium channels [37,64].

### 4.6. Prediction of Physicochemical and Pharmacokinetic Properties and Main Limitations

All five ligands complied with Lipinski’s Rule of Five, suggesting favorable oral bioavailability [66]. However, differences emerged under other pharmacokinetic filters [67]. According to Pfizer’s rule (LogP < 3) and the Golden Triangle (MW > 200 Da and LogP < 5), monoterpenes (LogP < 3) showed better solubility but may have reduced permeability, while sesquiterpenes (LogP > 3) were more hydrophobic, which may hinder gastrointestinal dissolution [68]. Most compounds demonstrated high predicted gastrointestinal absorption, with human intestinal absorption exceeding 90% [69]. Despite moderate Caco-2 permeability, the absence of P-glycoprotein interaction favors absorption via passive diffusion. The presence of hydrogen bond acceptors and the absence of donors further support membrane permeability [70].

The VDss values suggest tissue accumulation, particularly for sesquiterpenes, which showed higher VDss compared to monoterpenes [71]. All compounds are lipophilic and of low molecular weight, facilitating BBB permeability [72]. While advantageous for CNS-targeted drugs, this also raises concerns about potential neurotoxicity [73]. Major sesquiterpenes (aromadendrene and caryophyllene oxide) inhibit key CYP450 isoforms (CYP2C19, CYP2C9, CYP2D6, CYP3A4), indicating potential drug–drug interaction risks [74]. Other molecules showed minimal interaction with these enzymes, suggesting a lower likelihood of metabolic interference [75]. Monoterpenes exhibited faster clearance, potentially requiring more frequent dosing. Sesquiterpenes showed slower clearance, which may enhance efficacy but also increase accumulation and toxicity risk [76]. None of the compounds interacted with OCT2, reducing concerns about renal drug–drug interactions [77,78,79]. Toxicological predictions indicate low mutagenic, hepatotoxic, and hematological risk, as evidenced by negative Ames test results. Low or absent hERG channel inhibition suggests minimal cardiotoxicity. However, given the high BBB permeability, further studies are needed to evaluate the neurotoxicity risks [46,80,81].

## 5. Conclusions

The key original study was to demonstrate the synergistic interaction between terpenes and voltage-dependent calcium channels, suggesting a potential modulatory mechanism. While many studies in the literature on vasorelaxant agents highlight their effects and propose possible mechanisms of action, few provide an in-depth exploration of the molecular behavior of these compounds and their direct interaction with ion channels. Calcium channels play a central role in various physiological processes [82] by regulating the influx of this ion into the cell. Calcium is involved in essential mechanisms such as vascular smooth muscle contraction [83], neurotransmitter release [84], ATP production [85], bioenergetic processes [86], and blood pH homeostasis [87]. The modulation of these channels may therefore have implications not only for vascular function but also for other physiological processes [88,89,90]. This study accordingly contributes to the understanding of the effects of terpenes on calcium channels, and suggests a possible synergistic interaction that may be associated with their therapeutic potential. The results presented herein provide a basis for future investigations that support the identification of molecular interaction patterns of these small molecules with different biological targets and expanding perspectives for the development of novel therapeutic approaches.

We concluded from this study that the terpenes present in EOHc exert vasorelaxant effects on the isolated rat aorta through electromechanical coupling, independently of the endothelium. This effect occurs via the blockade and modulation of voltage-dependent calcium channels, with a synergistic interaction of EOHc molecules at key regions of these channels. Elucidation of the molecular behavior of these interactions may serve as a foundation for the development of pharmacological strategies, and expand the potential applications of small molecules in vascular function regulation and other calcium-mediated physiological processes.

## Data Availability

The original contributions presented in this study are included in the article/Supplementary Materials. Further inquiries can be directed to the corresponding author(s).

## Supplementary Materials

The following supporting information can be downloaded at: https://www.mdpi.com/article/10.3390/medsci14020262/s1, Table S1. GC-MS peak list of the essential oil, including retention time, peak area, height, and relative abundance. Figure S1. Total ion chromatogram (TIC) of the essential oil from *Hyptis crenata* obtained by GC-MS analysis. Figure S2. Mass spectrum of Peak 1 assigned to Bicyclo[3.1.0]hex-2-ene, 2-methyl-5-(1-methylethyl)-. Figure S3. Mass spectrum of Peak 2 assigned to 2,6,6-Trimethylbicyclo[3.1.1]hept-2-ene. Figure S4. Mass spectrum of Peak 3 assigned to Camphene. Figure S5. Mass spectrum of Peak 4 assigned to Bicyclo[3.1.1]heptane, 6,6-dimethyl-2-methylene-. Figure S6. Mass spectrum of Peak 5 assigned to β-Myrcene. Figure S7. Mass spectrum of Peak 6 assigned to 1,3-Cyclohexadiene, 1-methyl-4-(1-methylethyl)-. Figure S8. Mass spectrum of Peak 7 assigned to o-Cymene. Figure S9. Mass spectrum of Peak 8 assigned to D-Limonene. Figure S10. Mass spectrum of Peak 9 assigned to Eucalyptol. Figure S11. Mass spectrum of Peak 10 assigned to γ-Terpinene. Figure S12. Mass spectrum of Peak 11 assigned to Bicyclo[3.1.0]hexan-2-ol, 2-methyl-5-(1-methylethyl)-. Figure S13. Mass spectrum of Peak 12 assigned to 2-Carene. Figure S14. Mass spectrum of Peak 13 assigned to Bicyclo[3.1.0]hexan-2-ol, 2-methyl-5-(1-methylethyl)-. Figure S15. Mass spectrum of Peak 14 assigned to (+)-2-Bornanone. Figure S16. Mass spectrum of Peak 15 assigned to endo-Borneol. Figure S17. Mass spectrum of Peak 16 assigned to Terpinen-4-ol. Figure S18. Mass spectrum of Peak 17 assigned to α-Terpineol. Figure S19. Mass spectrum of Peak 18 assigned to Thymol. Figure S20. Mass spectrum of Peak 19 assigned to Phenol, 2-methyl-5-(1-methylethyl)-. Figure S21. Mass spectrum of Peak 20 assigned to Thymol. Figure S22. Mass spectrum of Peak 21 assigned to Isoledene. Figure S23. Mass spectrum of Peak 22 assigned to 1H-Cycloprop[e]azulene derivative. Figure S24. Mass spectrum of Peak 23 assigned to Caryophyllene. Figure S25. Mass spectrum of Peak 24 assigned to Guaia-1(10),11-diene. Figure S26. Mass spectrum of Peak 25 assigned to α-Panasinsen. Figure S27. Mass spectrum of Peak 26 assigned to Alloaromadendrene. Figure S28. Mass spectrum of Peak 27 assigned to α-Guaiene. Figure S29. Mass spectrum of Peak 28 assigned to 1,4,7-Cycloundecatriene derivative. Figure S30. Mass spectrum of Peak 29 assigned to Alloaromadendrene. Figure S31. Mass spectrum of Peak 30 assigned to Azulene derivative. Figure S32. Mass spectrum of Peak 31 assigned to 1H-Cycloprop[e]azulene derivative. Figure S33. Mass spectrum of Peak 32 assigned to Naphthalene derivative. Figure S34. Mass spectrum of Peak 33 assigned to Alloaromadendrene oxide-(1). Figure S35. Mass spectrum of Peak 34 assigned to Alloaromadendrene. Figure S36. Mass spectrum of Peak 35 assigned to (-)-Spathulenol. Figure S37. Mass spectrum of Peak 36 assigned to Caryophyllene oxide. Figure S38. Mass spectrum of Peak 37 assigned to 1H-Cycloprop[e]azulene derivative. Figure S39. Mass spectrum of Peak 38 assigned to 2-Naphthalenemethanol derivative. Figure S40. Mass spectrum of Peak 39 assigned to Caryophyllene. Figure S41. Mass spectrum of Peak 40 assigned to 8-epi-γ-eudesmol. Figure S42. Mass spectrum of Peak 41 assigned to Guaia-1(10),11-diene. Figure S43. Mass spectrum of Peak 42 assigned to Tetracyclo[6.3.2.0(2,5).0(1,8)]tridecan-9-ol derivative. Figure S44. Mass spectrum of Peak 43 assigned to (-)-Aristolene. Figure S45. Mass spectrum of Peak 44 assigned to Guaiol. Figure S46. Mass spectrum of Peak 45 assigned to Caryophyllene oxide. Figure S47. Mass spectrum of Peak 46 assigned to Cycloheptane derivative. Figure S48. Mass spectrum of Peak 47 assigned to Caryophyllene oxide. Table S2. Molecular docking interactions of selected compounds with the target protein across five predicted binding sites, including amino acid residues, interaction types, and binding affinities. Table S3. In silico physicochemical, pharmacokinetic, and toxicological profiling of Camphor, Caryophyllene, Caryophyllene Oxide, Cineole, and Aromadendrene.
