# Supplementary material for: L-Type Voltage-Gated Ca2+ Channels Are Targeted by Terpenes from Hyptis crenata Essential Oil in Vascular Electromechanical Coupling
Source: Med Sci (Basel). 2026 May 20;14(2):262. doi: 10.3390/medsci14020262 (PMC13214819; doi:10.3390/medsci14020262)
Supplement: Supplementary file 1 [file medsci-14-00262-s001.zip › medsci-4241147-supplementary.pdf]

TABLE S1

Table S1 - GCMS peak list of the essential oil, including Peak, Start, retention times

(RT), End, Height, Area, and Area Sum Percent.

Integration Peak List

Peak	Start	RT	End	Height	Area	AreaSumPercent
1	3.773	3.817	3.861	111080.22	177644.51	0.23
2	3.913	3.951	4.046	1471780.41	2198217.37	2.85
3	4.161	4.212	4.25	315406.25	510543.8	0.66
4	4.657	4.695	4.746	361230.08	606373.28	0.79
5	4.823	4.861	4.899	101280.13	173742.5	0.23
6	5.395	5.44	5.495	111312.17	200433.32	0.26
7	5.554	5.605	5.656	1644821.62	2991123.75	3.88
8	5.656	5.7	5.732	287387.83	587537.08	0.76
9	5.732	5.77	5.869	2935599.06	5649381.25	7.33
10	6.324	6.375	6.455	521074.78	1027510.87	1.33
11	6.534	6.578	6.693	129525.65	270499.73	0.35
12	7.056	7.113	7.195	83994.3	210663.93	0.27
13	7.291	7.348	7.425	159108.15	382464.48	0.5
14	8.576	8.646	8.799	8754472.31	21714420.2	28.16
15	9.168	9.219	9.371	255292.94	708185.86	0.92
16	9.479	9.543	9.619	313544.73	782744.39	1.02
17	9.855	9.918	10.02	329179.49	837439.65	1.09
18	12.661	12.724	12.851	309468.11	875905.11	1.14
19	12.966	13.023	13.138	178339.22	493060.01	0.64
20	13.138	13.208	13.316	191824.42	532962.3	0.69
21	15.403	15.479	15.53	66878.81	190691.35	0.25
22	16.56	16.611	16.809	62263.03	249527.79	0.32
23	16.828	16.91	17.044	4508833.99	12964999.62	16.81
24	17.092	17.152	17.222	104566.38	305633.5	0.4
25	17.311	17.362	17.413	127378.47	374299.89	0.49
26	17.413	17.489	17.566	1160153.72	3452748.34	4.48
27	17.566	17.617	17.673	112024.99	340744.1	0.44
28	17.852	17.922	18.03	215646.01	659575.39	0.86
29	18.03	18.138	18.237	218237.15	723757.59	0.94
30	18.844	18.965	19.01	75091.35	269953.21	0.35
31	19.086	19.156	19.245	558421.24	1677458.85	2.18
32	19.913	19.971	20.032	58134.3	169588.94	0.22
33	20.76	20.829	20.886	73528.81	220925.69	0.29
34	20.963	21.02	21.078	50896.7	151105.11	0.2
35	21.466	21.542	21.599	339732.87	1025406.55	1.33
36	21.618	21.714	21.873	1546468.57	5602079.24	7.27
37	21.873	21.949	22.057	333497.9	1127344.11	1.46
38	22.172	22.242	22.388	59037.8	310772.38	0.4
39	22.388	22.445	22.515	42826.82	154835.68	0.2
40	22.655	22.744	22.891	548605.78	1863354.01	2.42
41	22.929	23.062	23.12	32058.93	207474.04	0.27
42	23.12	23.19	23.349	175417.19	813840.79	1.06
43	23.349	23.419	23.521	86504.46	378360.98	0.49
44	23.521	23.648	23.718	209459.73	880246.55	1.14
45	23.718	23.775	23.877	428387.99	1412449.98	1.83
46	23.877	23.991	24.074	26262.13	259014.56	0.34
47	24.074	24.131	24.341	81181.52	389830.73	0.51

FIGURE S1

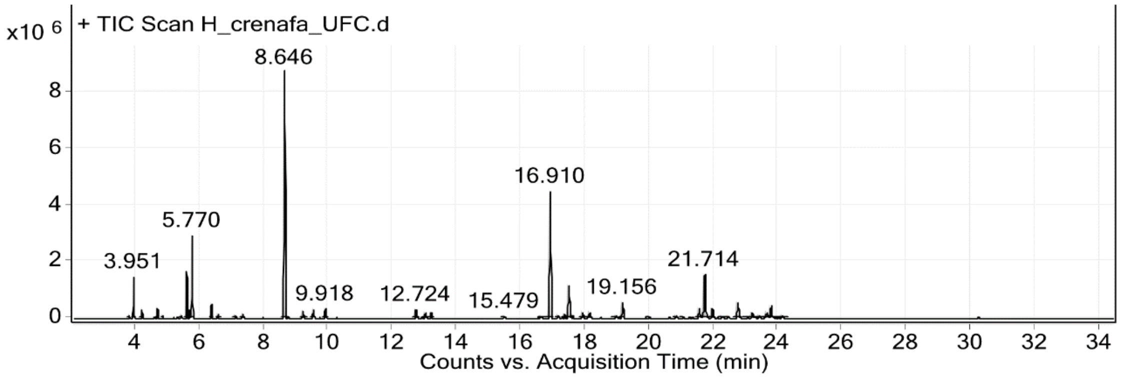

Figure S1 – Total Ion Chromatogram (TIC) of the essential oil from *Hyptis crenata* obtained by GC-MS analysis. The peaks represent the volatile constituents identified in the sample, with major components indicated by their respective retention times (min).

FIGURE S2

A **Spectrum Source**
Peak (1) in "+ TIC Scan"

Collision Energy
0

Ionization Mode
Unspecified

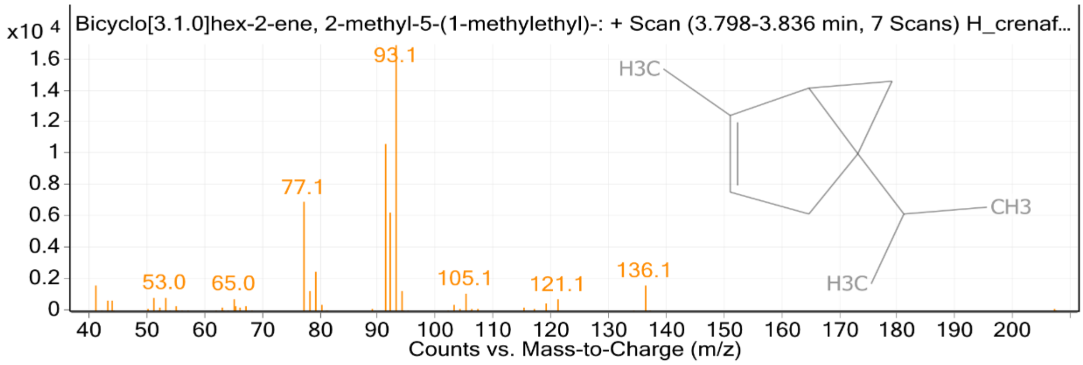

B **Library Spectrum**

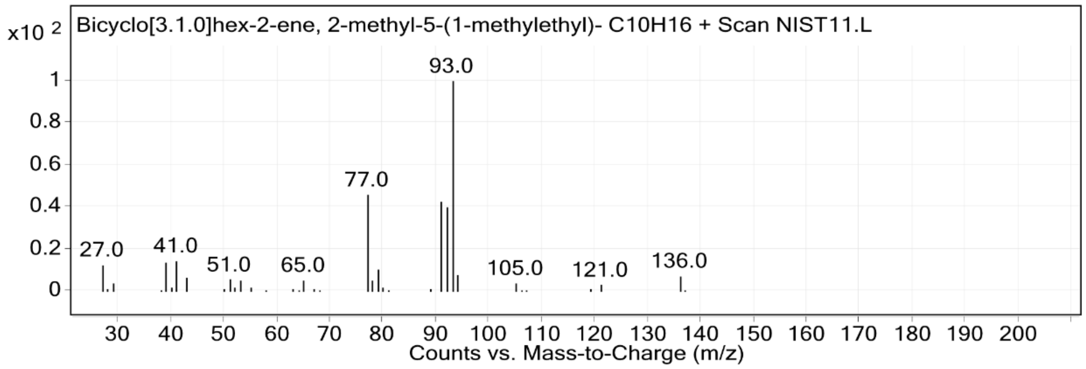

C **Spectrum Structure**
Bicyclo[3.1.0]hex-2-ene, 2-methyl-5-(1-methylethyl)-

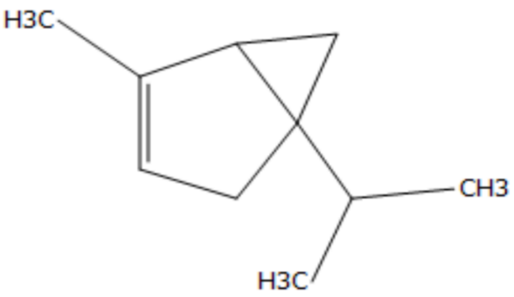

Figure S2 - Mass spectrum of Peak 1 from the TIC chromatogram, assigned to **Bicyclo[3.1.0]hex-2-ene, 2-methyl-5-(1-methylethyl)-.** (A) Experimental mass spectrum extracted from the chromatographic analysis. (B) Reference mass spectrum from the NIST11 library used for identification. (C) Chemical structure of the identified compound.

FIGURE S3

A **Spectrum Source** Peak (2) in "+ TIC Scan" | **Collision Energy** 0 | **Ionization Mode** Unspecified

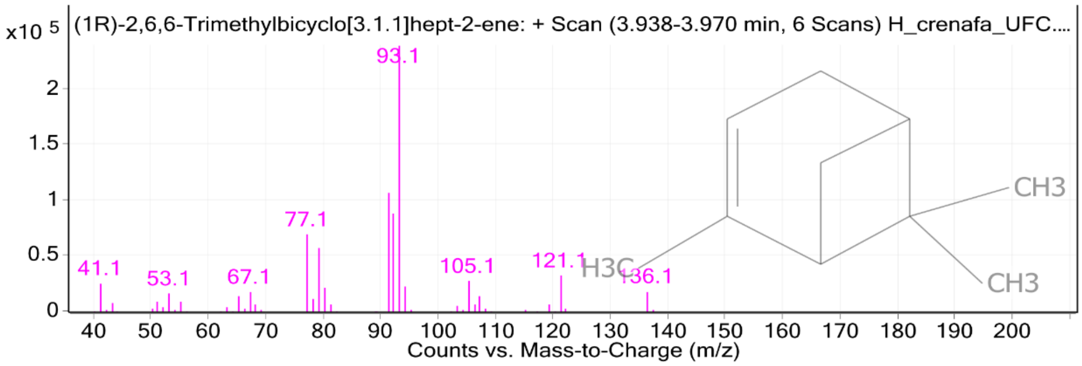

B **Library Spectrum**

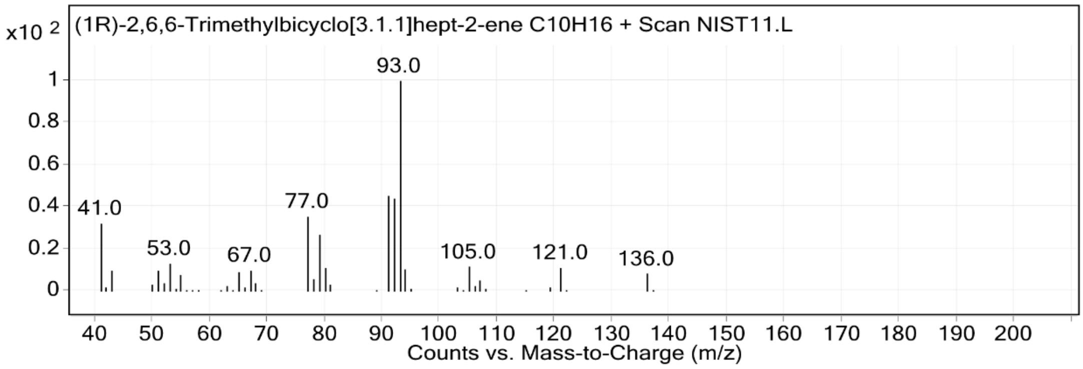

C **Spectrum Structure**
(1R)-2,6,6-Trimethylbicyclo[3.1.1]hept-2-ene

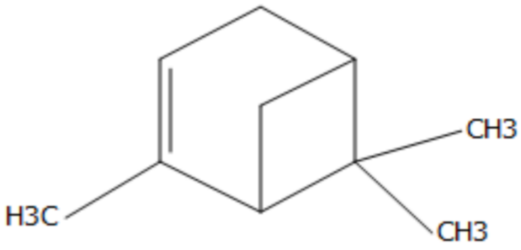

Figure S3 - Mass spectrum of Peak 2 from the TIC chromatogram, assigned to **(IR) – 2,6,6-Trimethylbicyclo[3. 1. 1]hept-2-ene.** (A) Experimental mass spectrum extracted from the chromatographic analysis. (B) Reference mass spectrum from the NIST11 library used for identification. (C) Chemical structure of the identified compound.

FIGURE S4

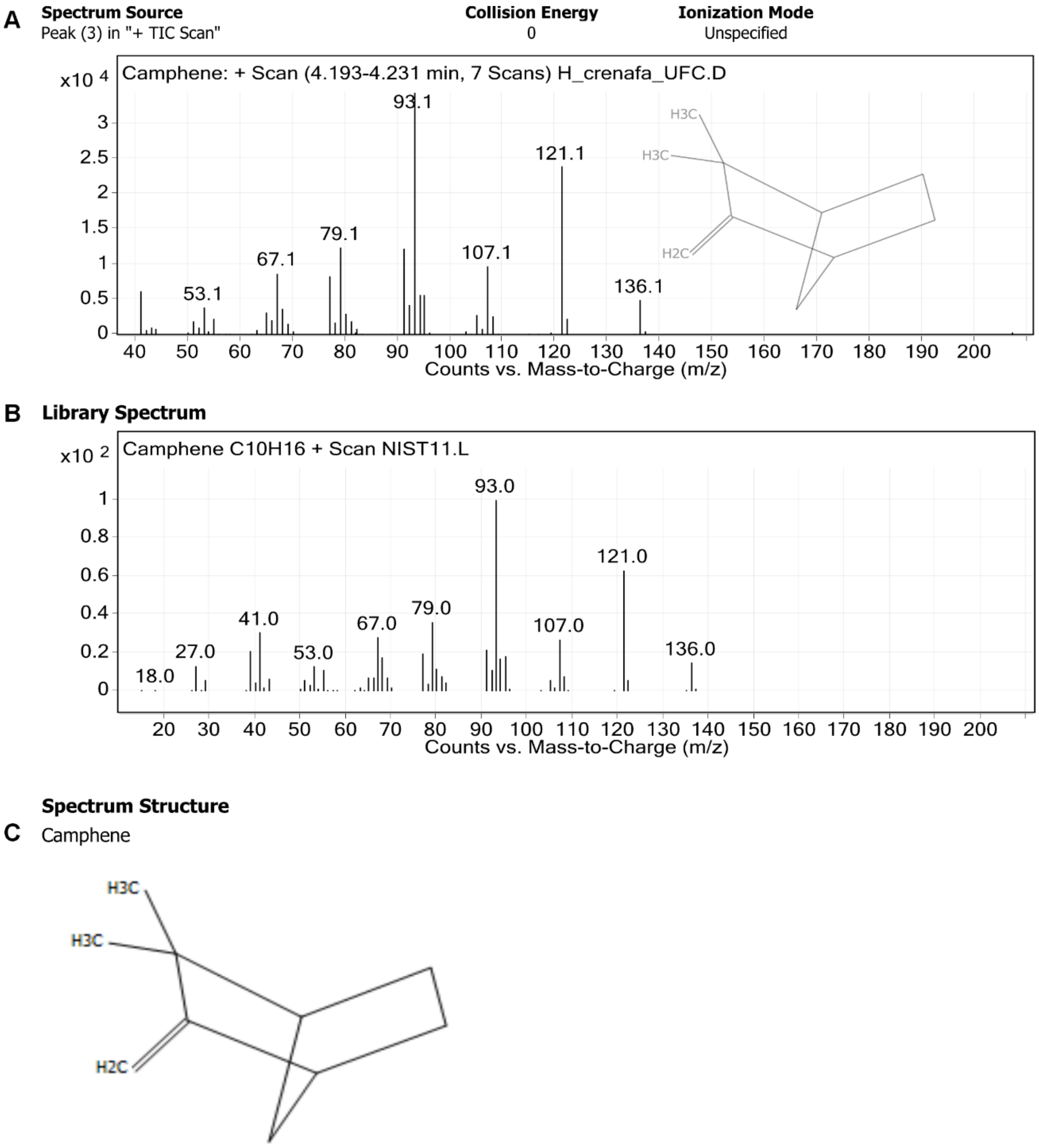

Figure S4 - Mass spectrum of Peak 3 from the TIC chromatogram, assigned to **Camphene.** (A) Experimental mass spectrum extracted from the chromatographic analysis. (B) Reference mass spectrum from the NIST11 library used for identification. (C) Chemical structure of the identified compound.

FIGURE S5

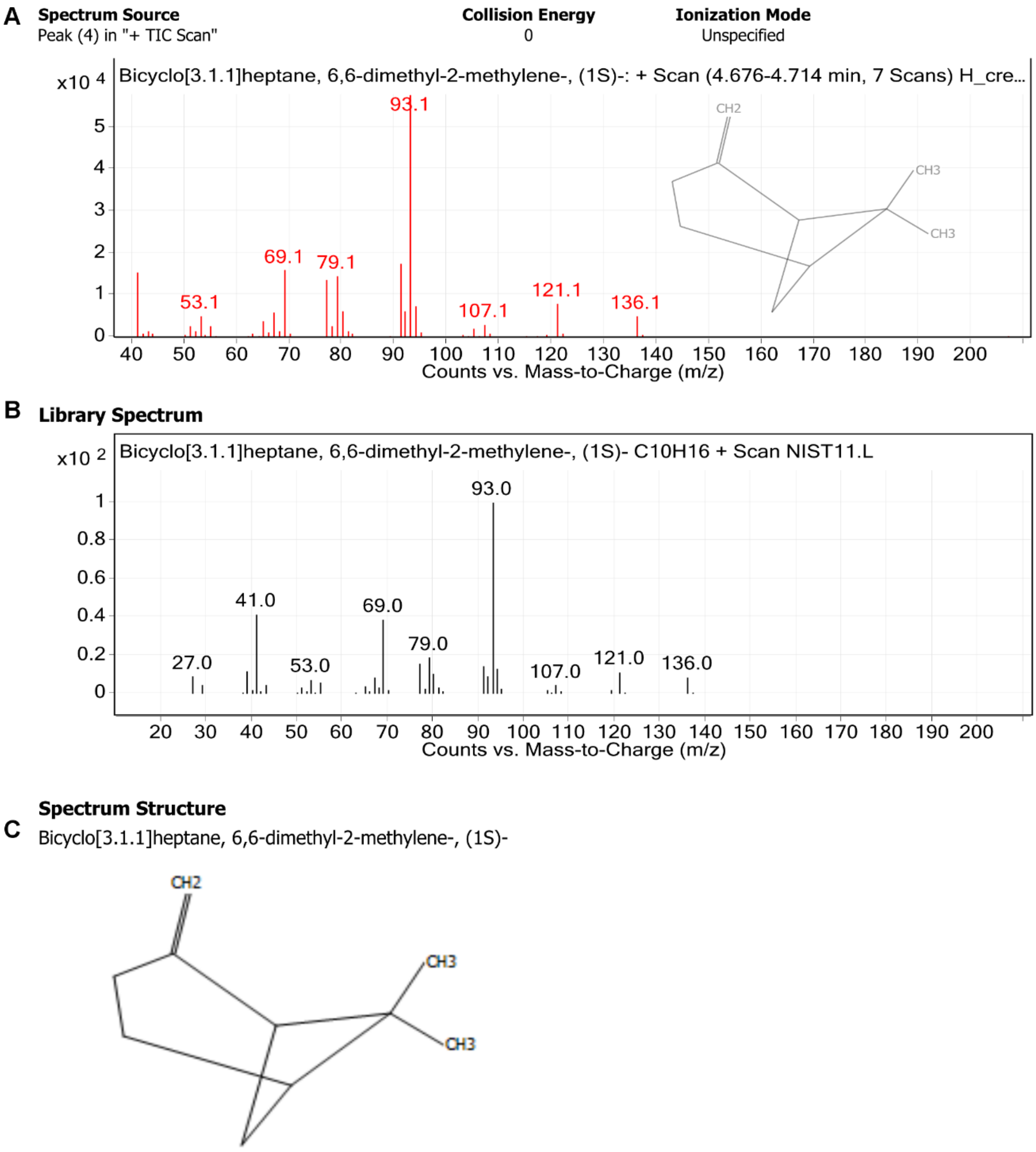

Figure S5 - Mass spectrum of Peak 4 from the TIC chromatogram, assigned to **Bicyclo[3. 1. 1]heptane, 6, 6-dimethyl-2-methylene-**. (A) Experimental mass spectrum extracted from the chromatographic analysis. (B) Reference mass spectrum from the NIST11 library used for identification. (C) Chemical structure of the identified compound.

FIGURE S6

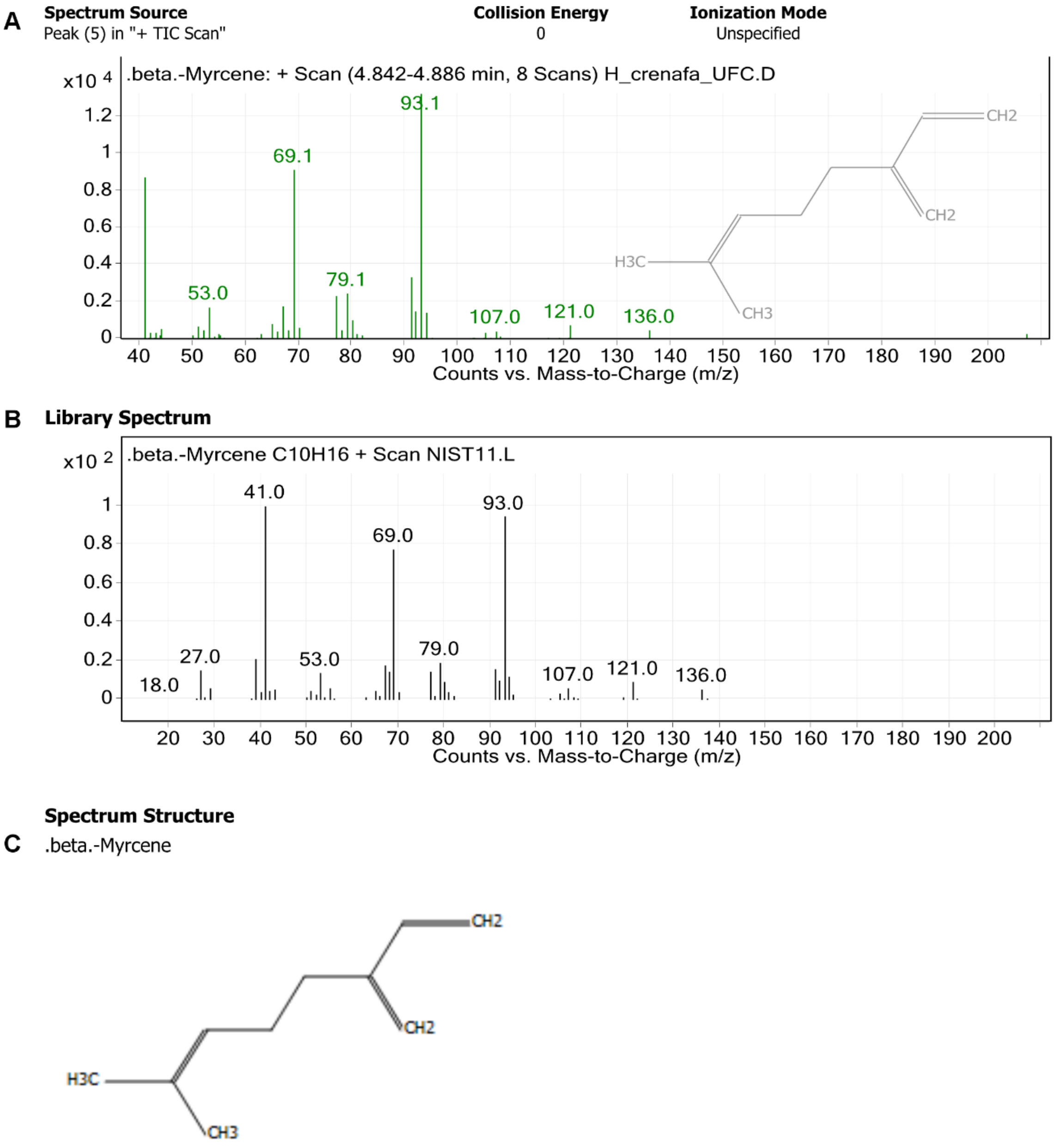

Figure S6 - Mass spectrum of Peak 5 from the TIC chromatogram, assigned to **.beta.-Myrcene.** (A) Experimental mass spectrum extracted from the chromatographic analysis. (B) Reference mass spectrum from the NIST11 library used for identification. (C) Chemical structure of the identified compound.

FIGURE S7

A **Spectrum Source** Peak (6) in "+ TIC Scan" | **Collision Energy** 0 | **Ionization Mode** Unspecified

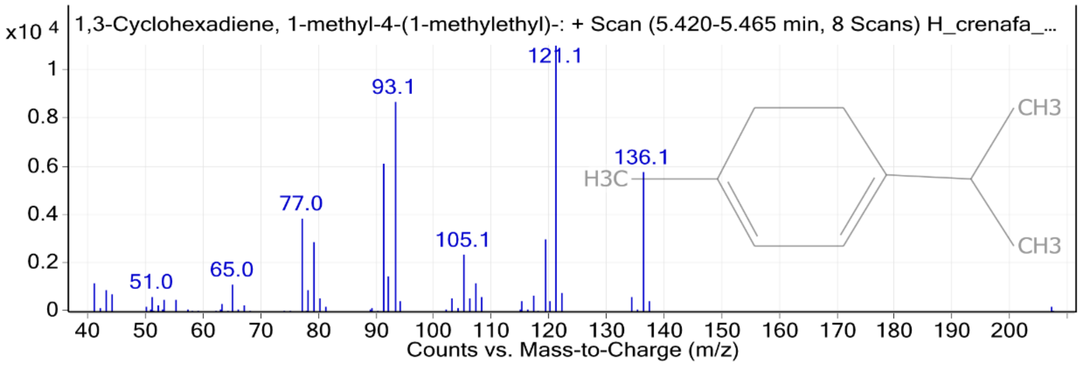

B **Library Spectrum**

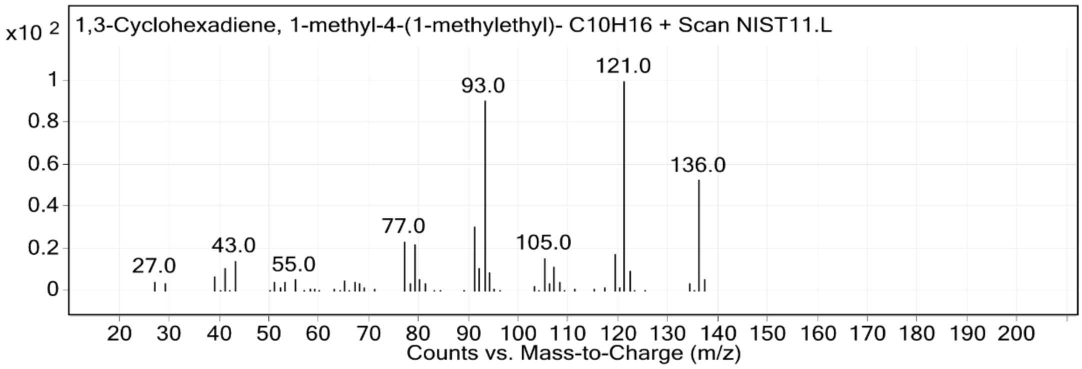

Spectrum Structure

C 1,3-Cyclohexadiene, 1-methyl-4-(1-methylethyl)-

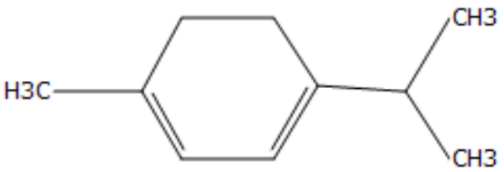

Figure S7 - Mass spectrum of Peak 6 from the TIC chromatogram, assigned to **1,3-Cyclohexadiene, 1-methyl-4-(1-methylethyl)-.** (A) Experimental mass spectrum extracted from the chromatographic analysis. (B) Reference mass spectrum from the NIST11 library used for identification. (C) Chemical structure of the identified compound.

FIGURE S8

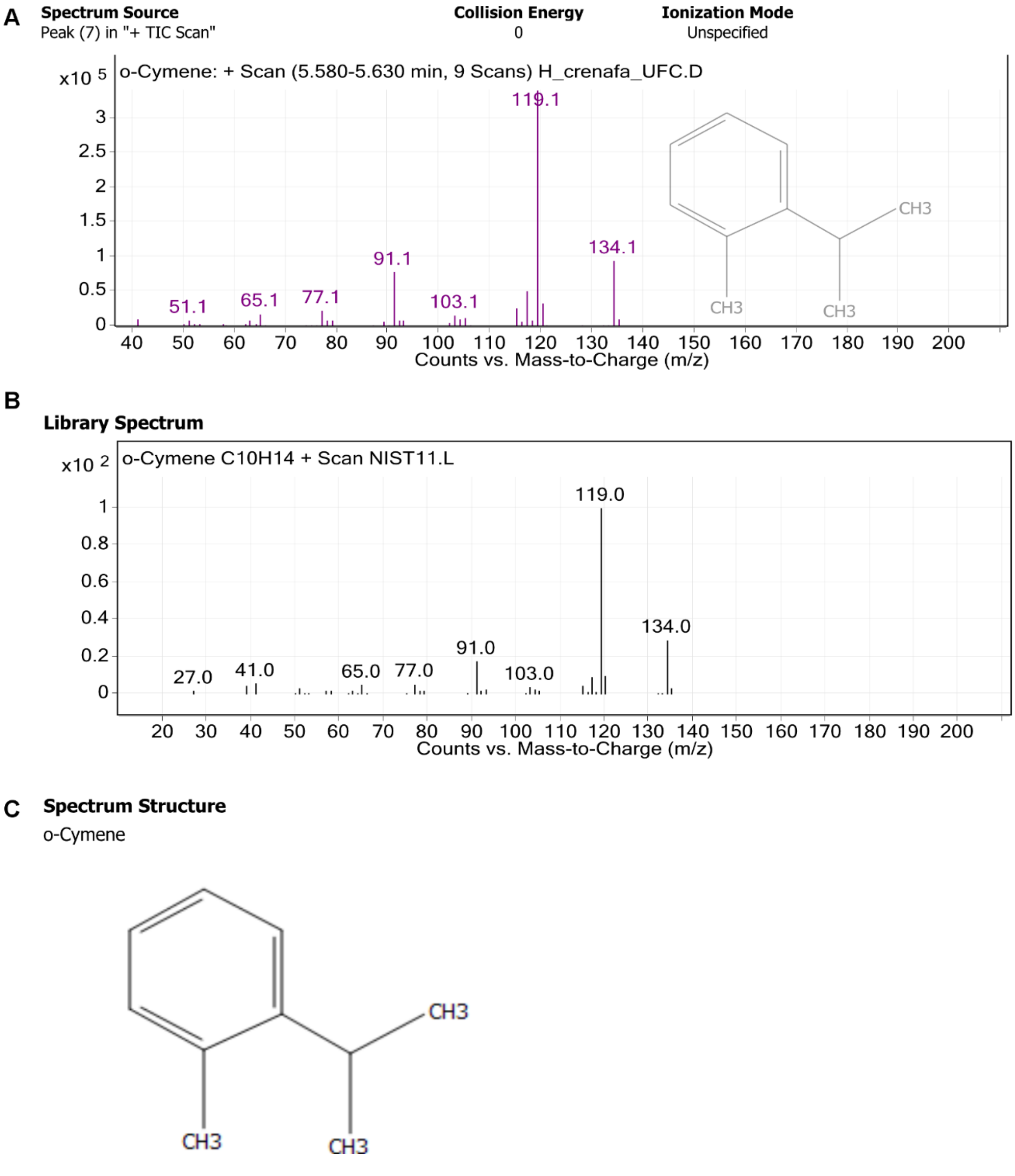

Figure S8 - Mass spectrum of Peak 7 from the TIC chromatogram, assigned to **o-Cymene.** (A) Experimental mass spectrum extracted from the chromatographic analysis. (B) Reference mass spectrum from the NIST11 library used for identification. (C) Chemical structure of the identified compound.

FIGURE S9

A **Spectrum Source** Peak (8) in "+ TIC Scan" — **Collision Energy** 0 — **Ionization Mode** Unspecified

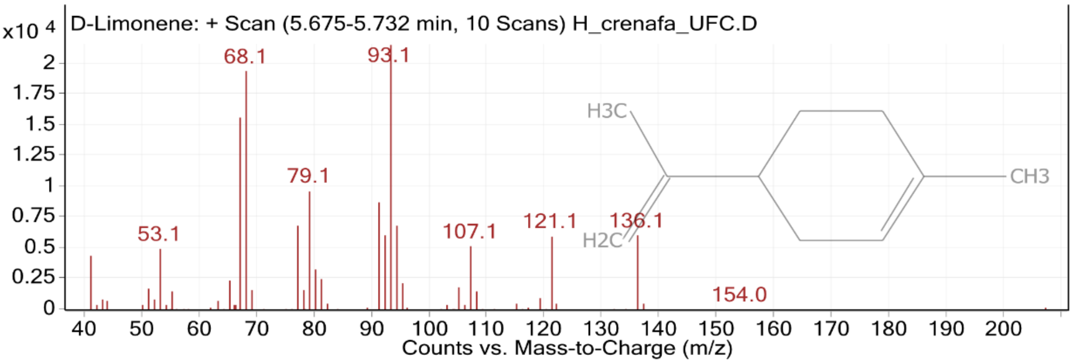

B **Library Spectrum**

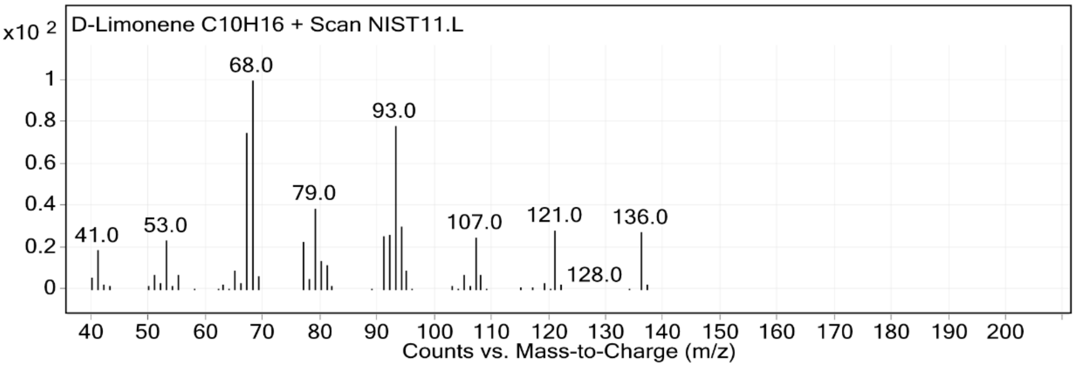

C **Spectrum Structure**
D-Limonene

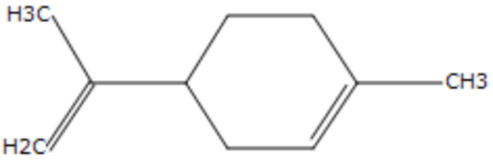

Figure S9 - Mass spectrum of Peak 8 from the TIC chromatogram, assigned to **D-Limonene.** (A) Experimental mass spectrum extracted from the chromatographic analysis. (B) Reference mass spectrum from the NIST11 library used for identification. (C) Chemical structure of the identified compound.

FIGURE S10

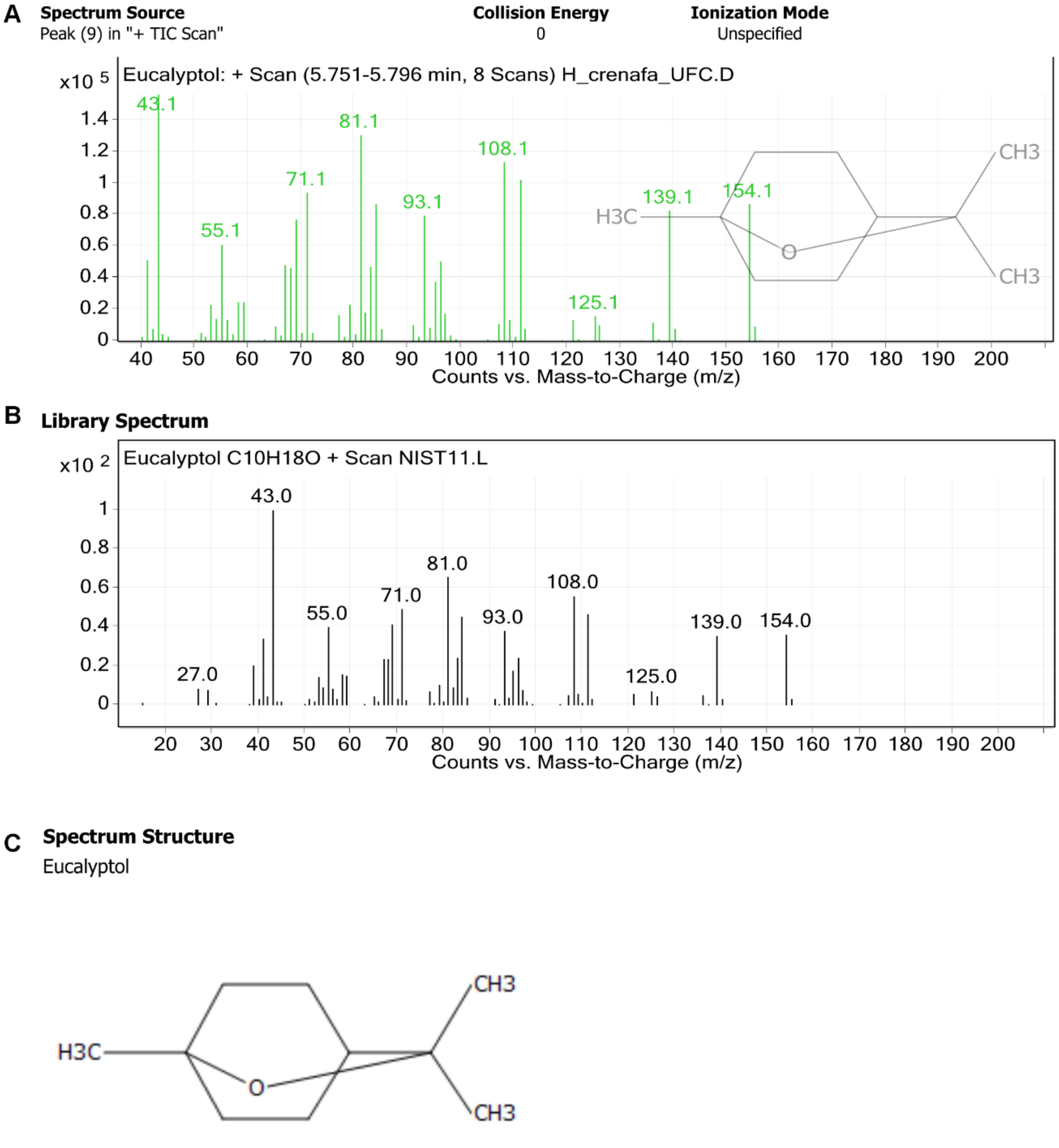

Figure S10 - Mass spectrum of Peak 9 from the TIC chromatogram, assigned to **Eucalyptol**. (A) Experimental mass spectrum extracted from the chromatographic analysis. (B) Reference mass spectrum from the NIST11 library used for identification. (C) Chemical structure of the identified compound.

FIGURE S11

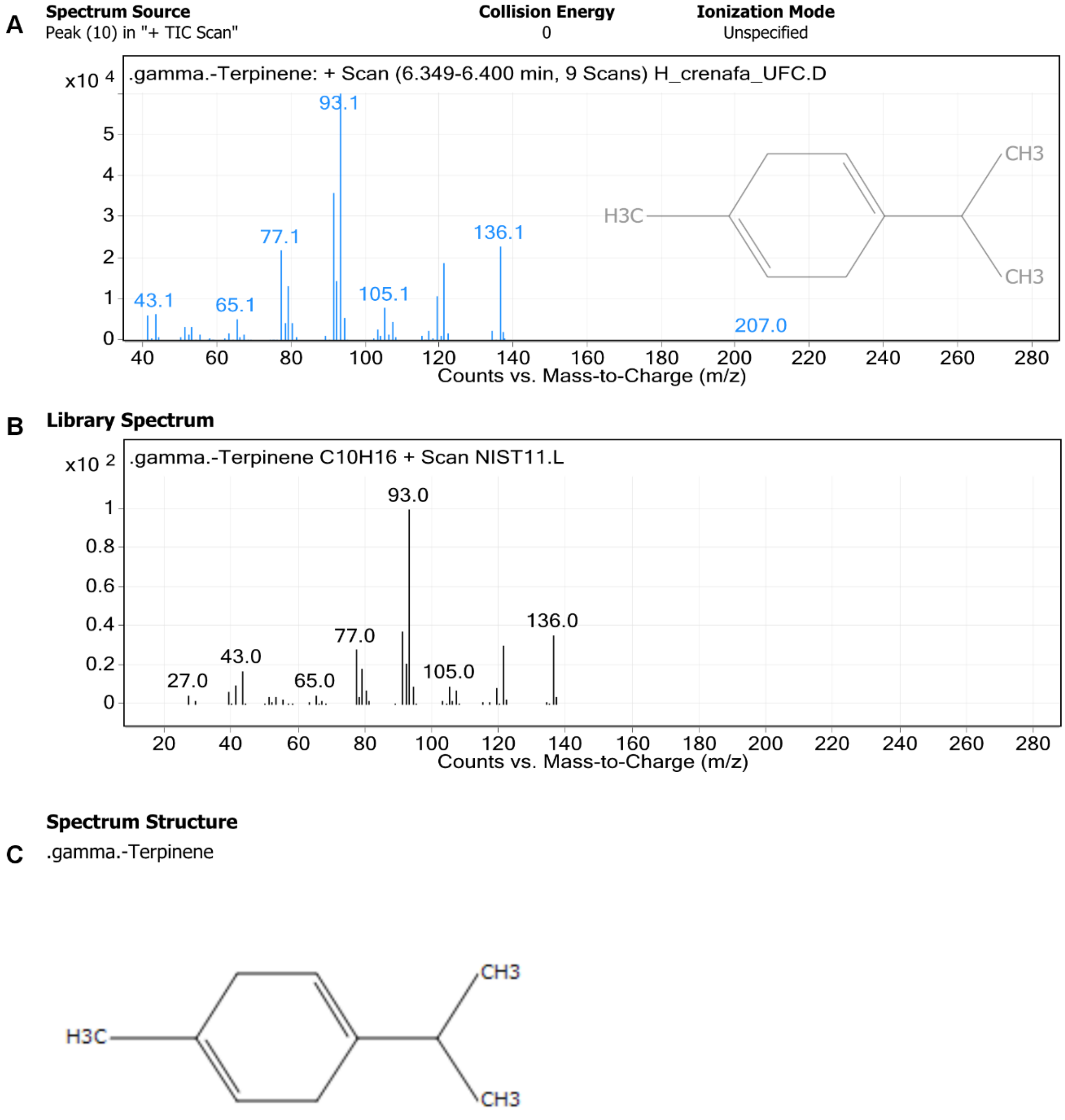

Figure S11 - Mass spectrum of Peak 10 from the TIC chromatogram, assigned to **.gamma.-Terpinene.** (A) Experimental mass spectrum extracted from the chromatographic analysis. (B) Reference mass spectrum from the NIST11 library used for identification. (C) Chemical structure of the identified compound.

FIGURE S12

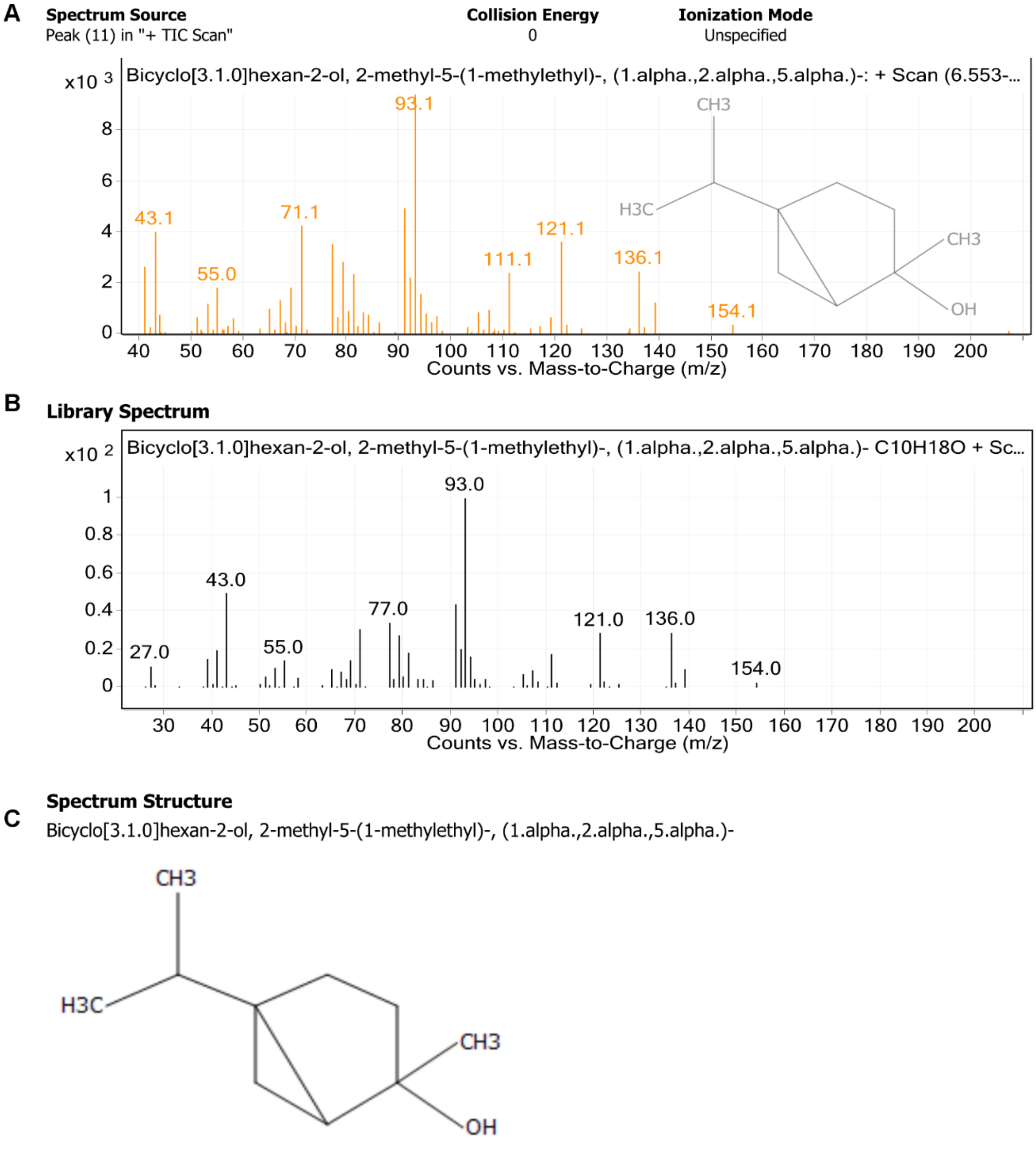

Figure S12 - Mass spectrum of Peak 11 from the TIC chromatogram, assigned to **Bicyclo[3.1.0]hexan-2-ol, 2-methyl-5-(1-methylethyl)-, (1-alpha., 2.alpha., 5.alpha.).** (A) Experimental mass spectrum extracted from the chromatographic analysis. (B) Reference mass spectrum from the NIST11 library used for identification. (C) Chemical structure of the identified compound.

FIGURE S13

A **Spectrum Source**
Peak (12) in "+ TIC Scan"

Collision Energy
0

Ionization Mode
Unspecified

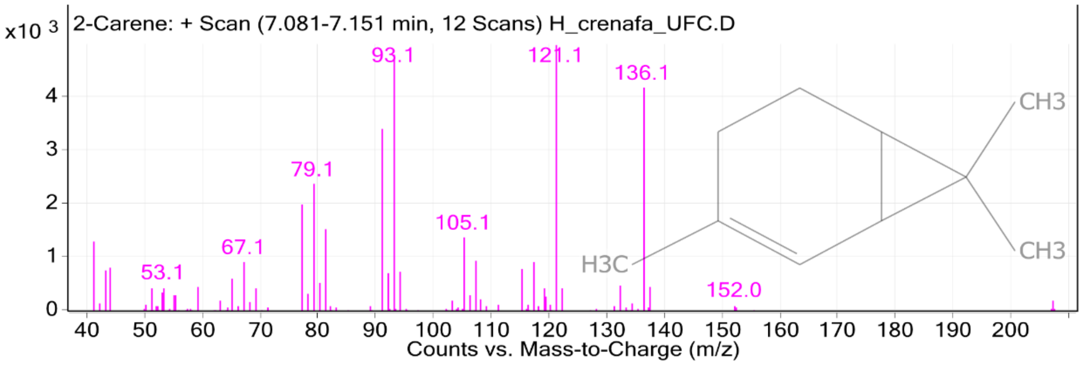

B **Library Spectrum**

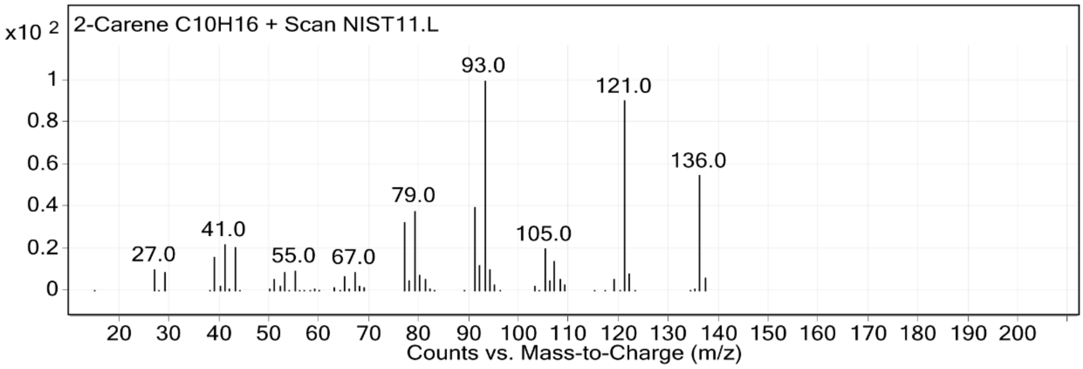

C **Spectrum Structure**
2-Carene

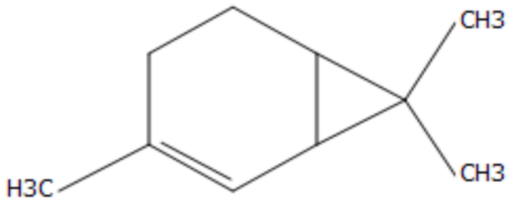

Figure S13 - Mass spectrum of Peak 12 from the TIC chromatogram, assigned to **2-Carene.** (A) Experimental mass spectrum extracted from the chromatographic analysis. (B) Reference mass spectrum from the NIST11 library used for identification. (C) Chemical structure of the identified compound.

FIGURE S14

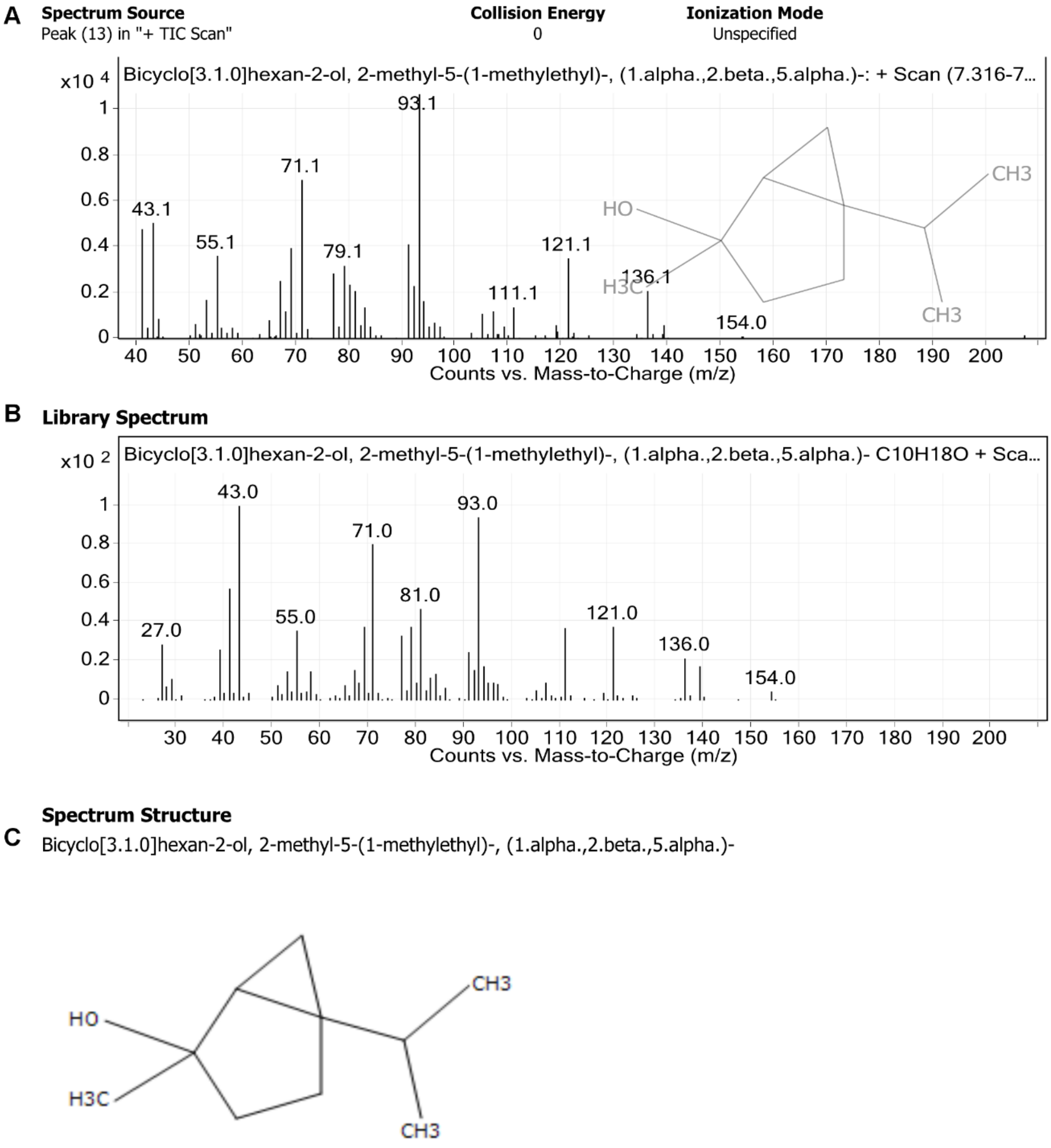

Figure S14 - Mass spectrum of Peak 13 from the TIC chromatogram, assigned to **Bicyclo[3.1.0]hexan-2-ol, 2-methyl-5-(1-methylethyl)-, (1-alpha., 2.alpha., 5.alpha.)** for the second time. (A) Experimental mass spectrum extracted from the chromatographic analysis. (B) Reference mass spectrum from the NIST11 library used for identification. (C) Chemical structure of the identified compound.

FIGURE S15

A **Spectrum Source** Peak (14) in "+ TIC Scan" | **Collision Energy** 0 | **Ionization Mode** Unspecified

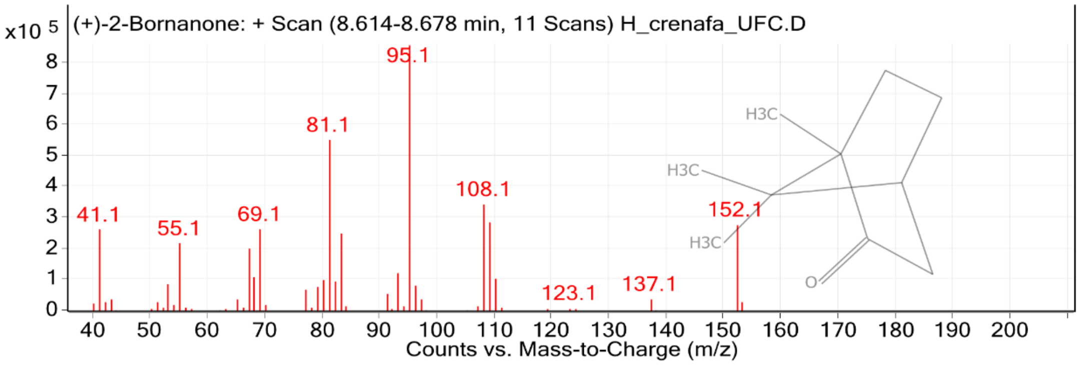

B **Library Spectrum**

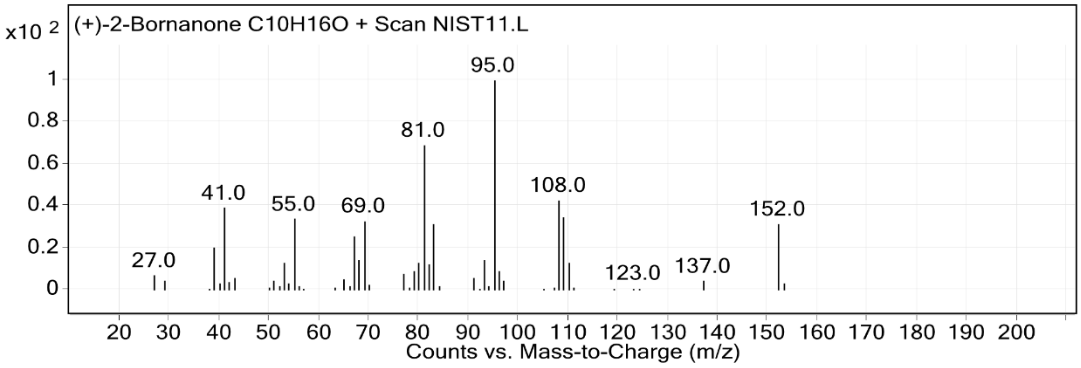

C **Spectrum Structure**
(+)-2-Bornanone

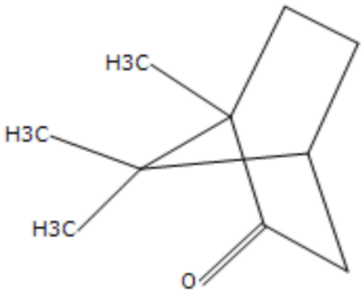

Figure S15 - Mass spectrum of Peak 14 from the TIC chromatogram, assigned to **(+)-2-Bornanone.** (A) Experimental mass spectrum extracted from the chromatographic analysis. (B) Reference mass spectrum from the NIST11 library used for identification. (C) Chemical structure of the identified compound.

FIGURE S16

A **Spectrum Source** Peak (15) in "+ TIC Scan" | **Collision Energy** 0 | **Ionization Mode** Unspecified

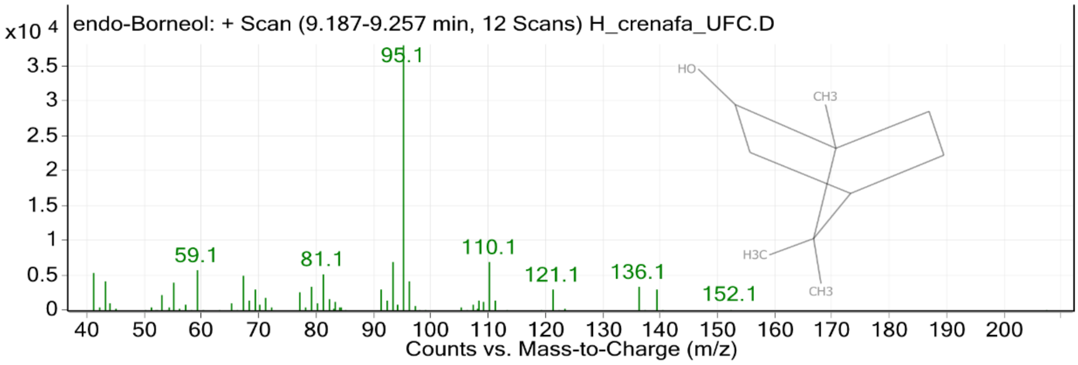

B **Library Spectrum**

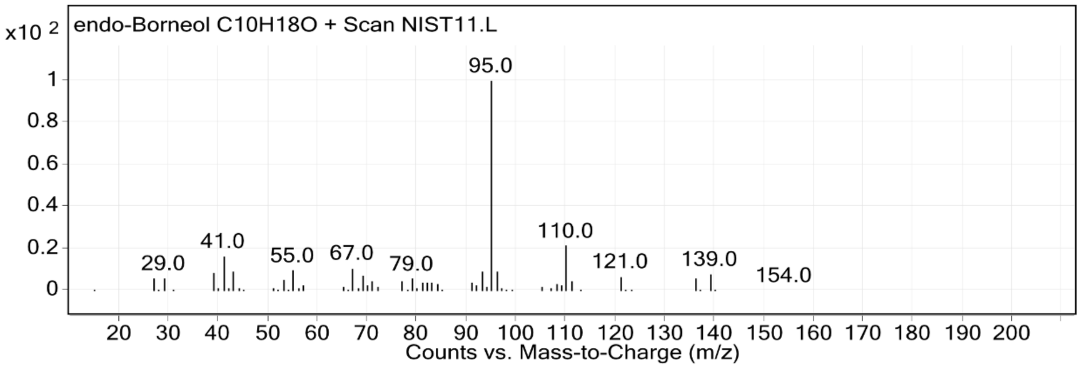

Spectrum Structure

C endo-Borneol

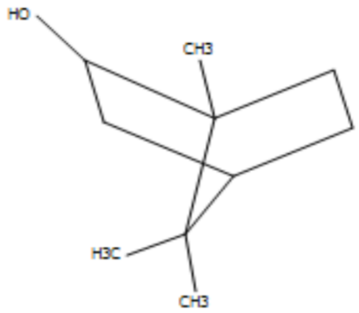

Figure S16 - Mass spectrum of Peak 15 from the TIC chromatogram, assigned to **endo-Borneol.** (A) Experimental mass spectrum extracted from the chromatographic analysis. (B) Reference mass spectrum from the NIST11 library used for identification. (C) Chemical structure of the identified compound.

FIGURE S17

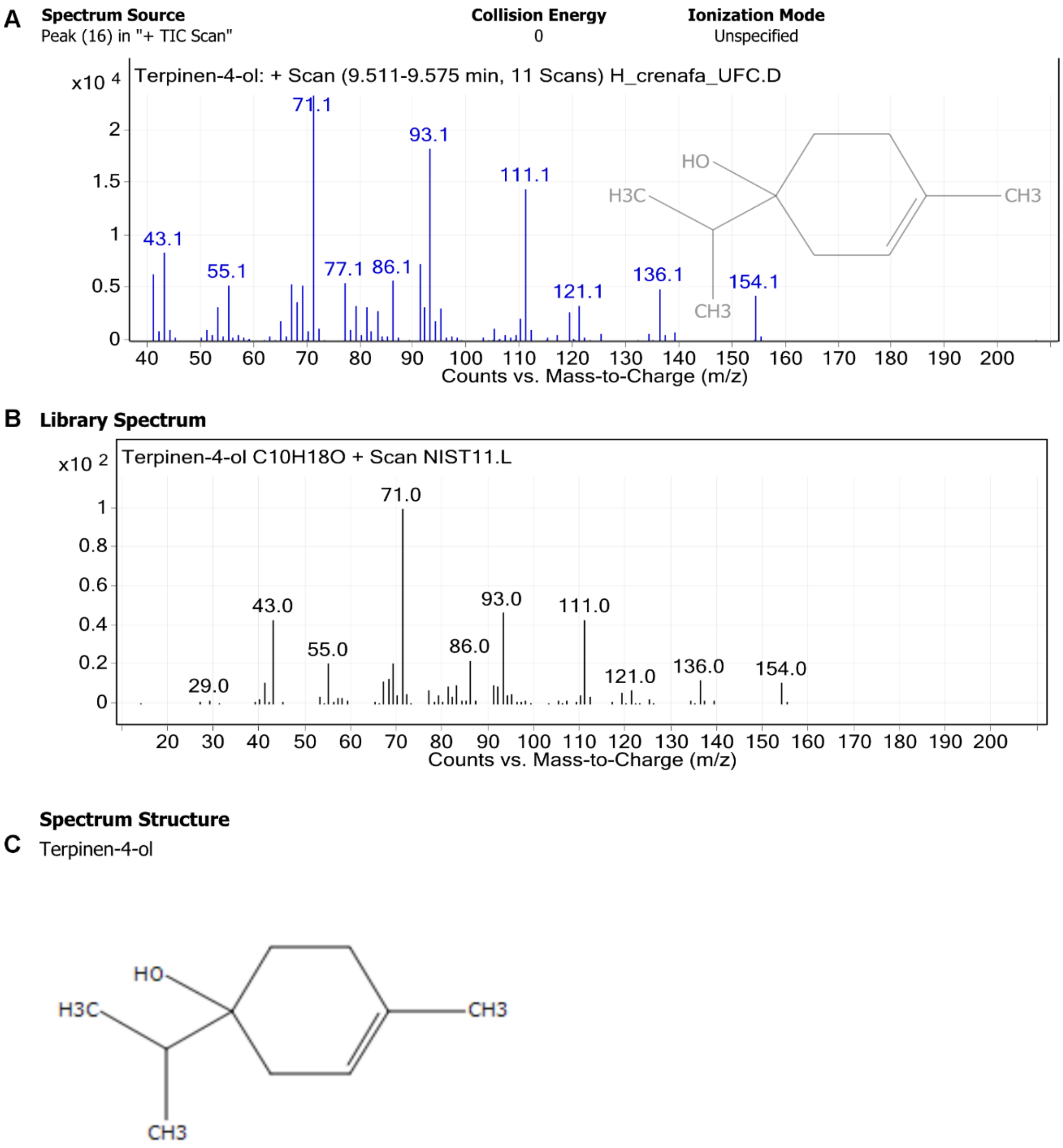

Figure S17 - Mass spectrum of Peak 16 from the TIC chromatogram, assigned to **Terpinen-4ol.** (A) Experimental mass spectrum extracted from the chromatographic analysis. (B) Reference mass spectrum from the NIST11 library used for identification. (C) Chemical structure of the identified compound.

FIGURE S18

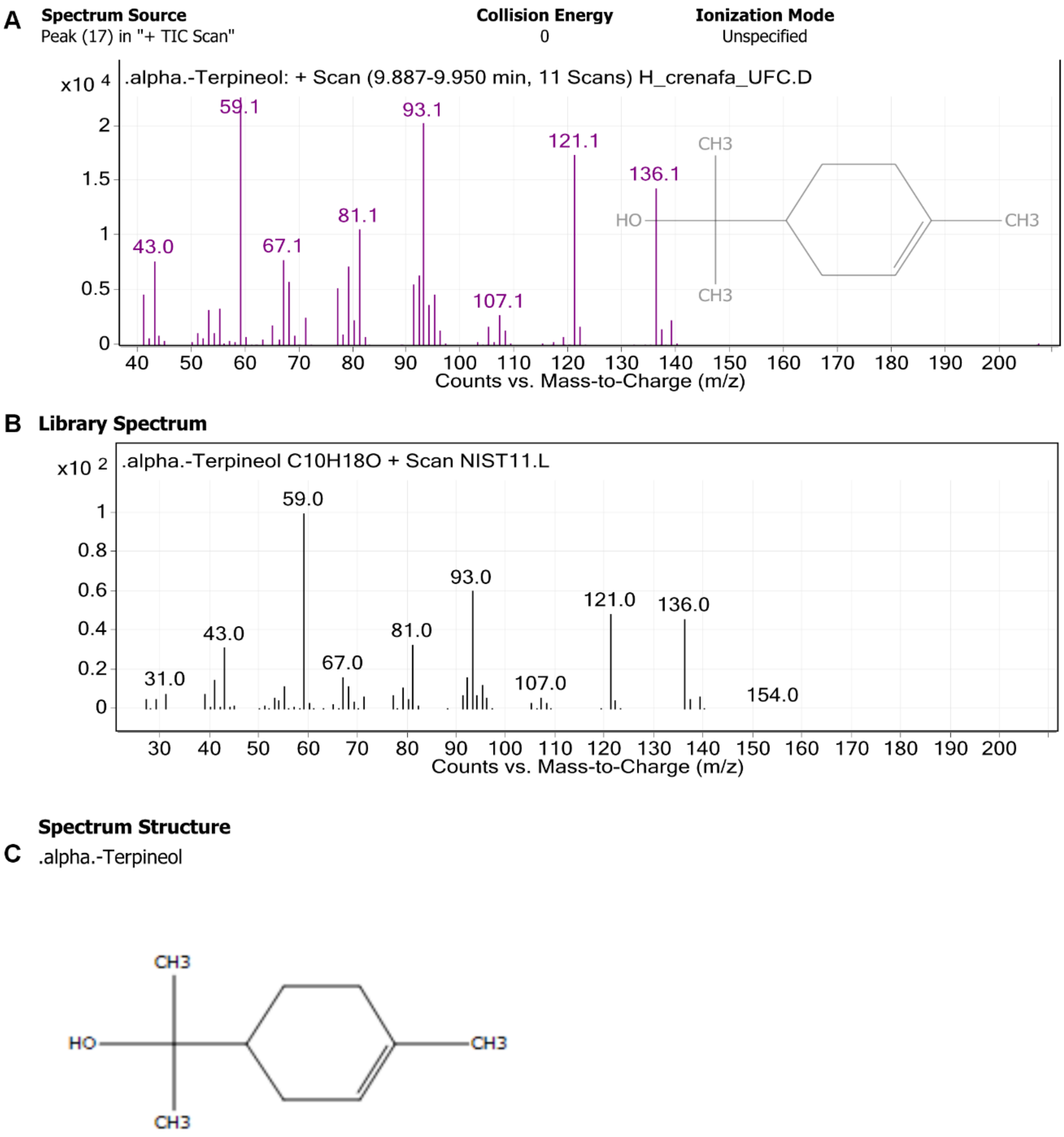

Figure S18 - Mass spectrum of Peak 17 from the TIC chromatogram, assigned to **.alpha.-Terpineol.** (A) Experimental mass spectrum extracted from the chromatographic analysis. (B) Reference mass spectrum from the NIST11 library used for identification. (C) Chemical structure of the identified compound.

FIGURE S19

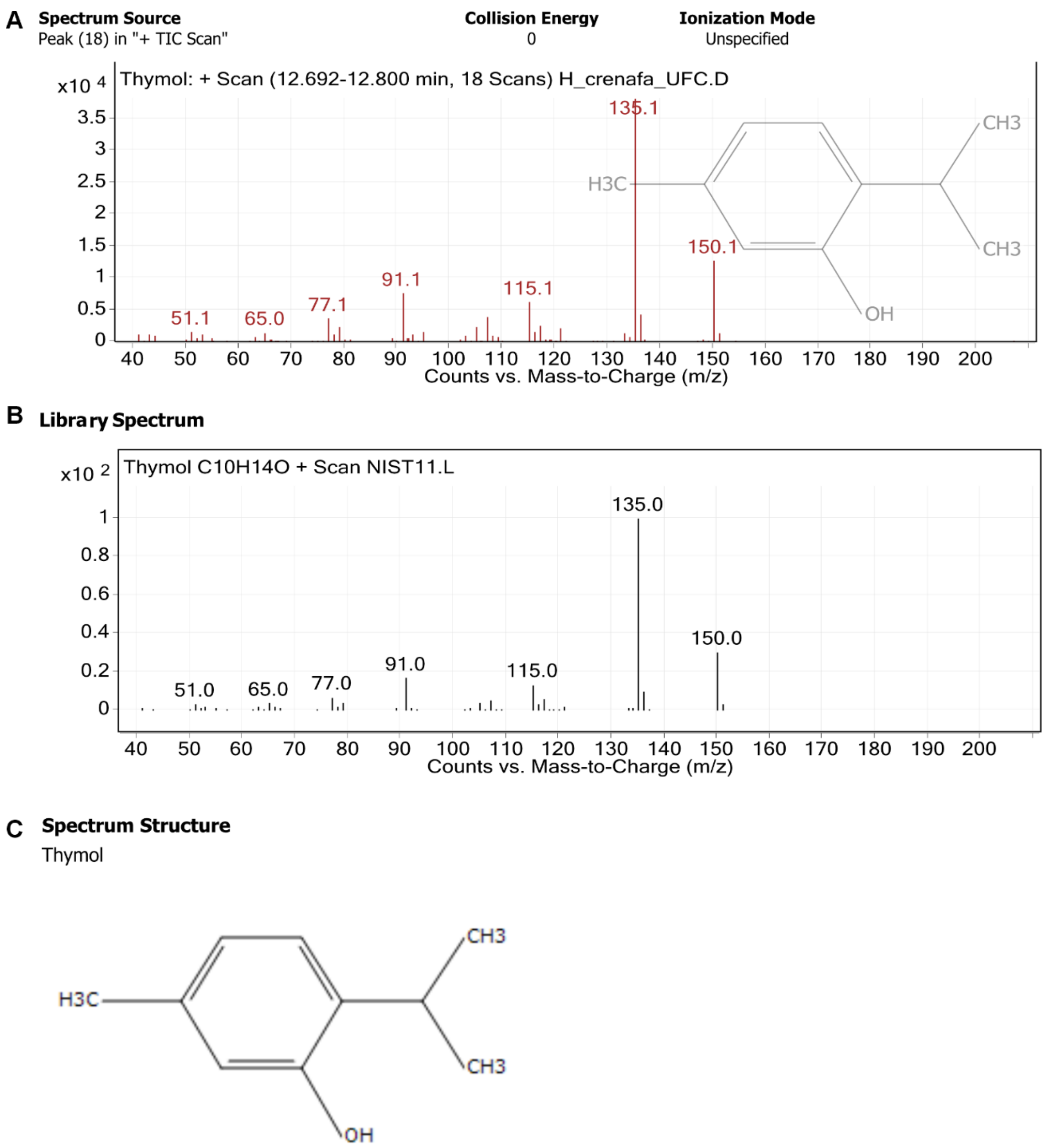

Figure S19 - Mass spectrum of Peak 18 from the TIC chromatogram, assigned to **Thymol.** (A) Experimental mass spectrum extracted from the chromatographic analysis. (B) Reference mass spectrum from the NIST11 library used for identification. (C) Chemical structure of the identified compound.

FIGURE S20

A **Spectrum Source** Peak (19) in "+ TIC Scan" — **Collision Energy** 0 — **Ionization Mode** Unspecified

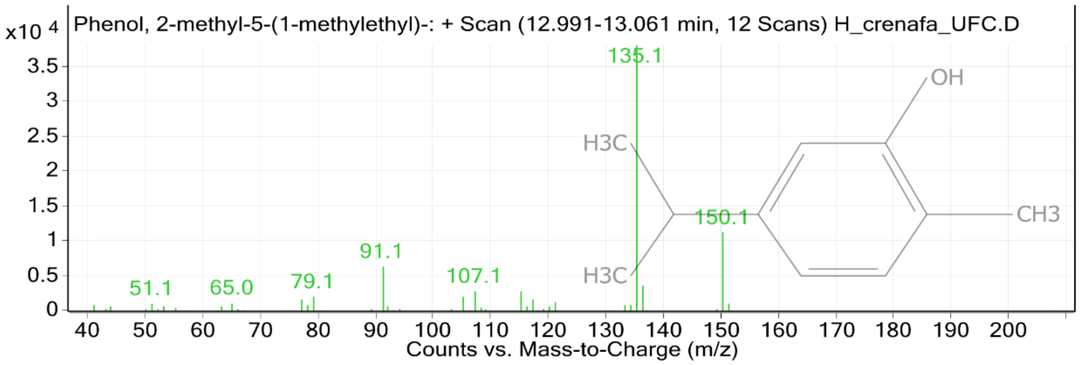

B **Library Spectrum**

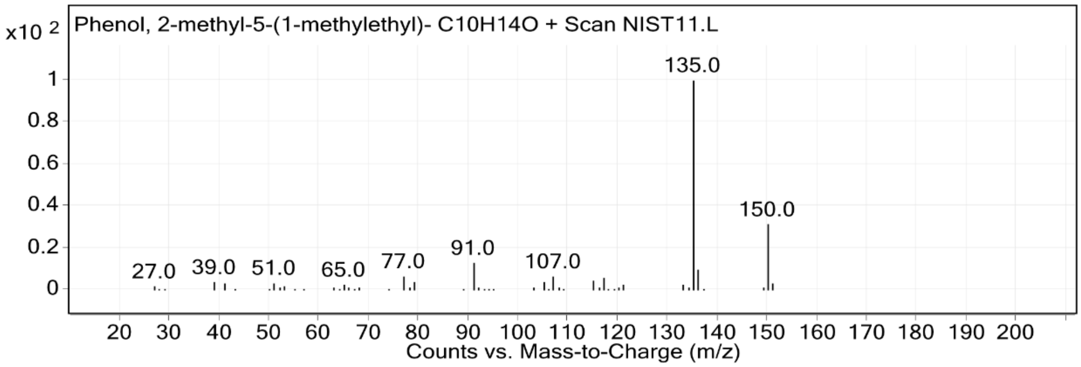

C **Spectrum Structure**

Phenol, 2-methyl-5-(1-methylethyl)-

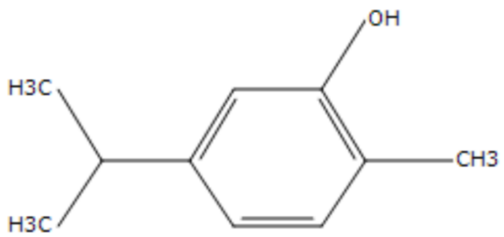

Figure S20 - Mass spectrum of Peak 19 from the TIC chromatogram, assigned to **Phenol, 2-methyl-5-(1-methylethyl)-.** (A) Experimental mass spectrum extracted from the chromatographic analysis. (B) Reference mass spectrum from the NIST11 library used for identification. (C) Chemical structure of the identified compound.

FIGURE S21

A **Spectrum Source** Peak (20) in "+ TIC Scan" **Collision Energy** 0 **Ionization Mode** Unspecified

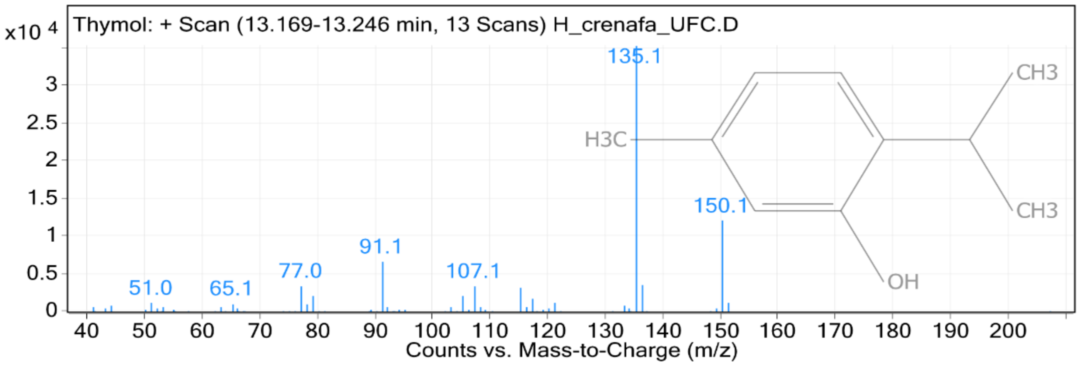

B **Library Spectrum**

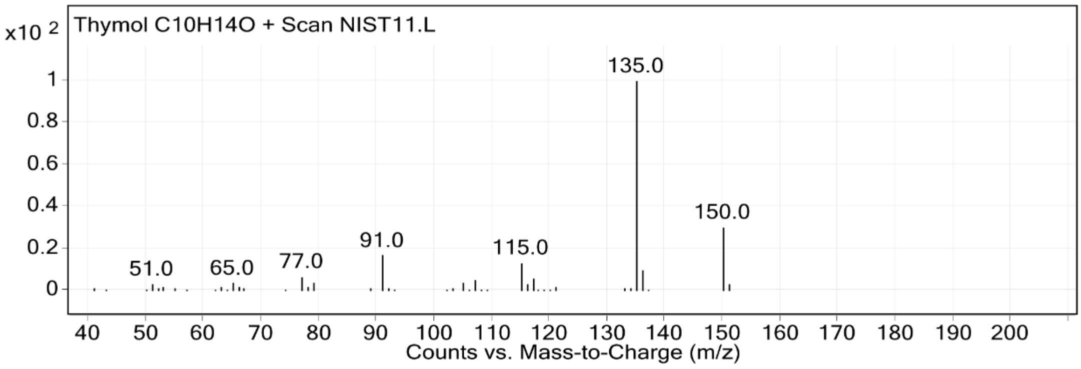

C **Spectrum Structure**
Thymol

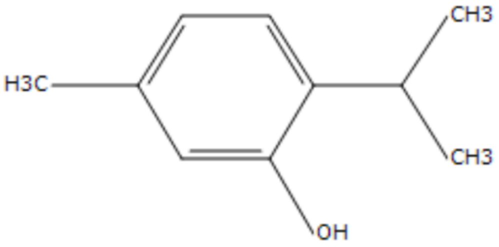

Figure S21 - Mass spectrum of Peak 20 from the TIC chromatogram, assigned to **Thymol.** (A) Experimental mass spectrum extracted from the chromatographic analysis. (B) Reference mass spectrum from the NIST11 library used for identification. (C) Chemical structure of the identified compound.

FIGURE S22

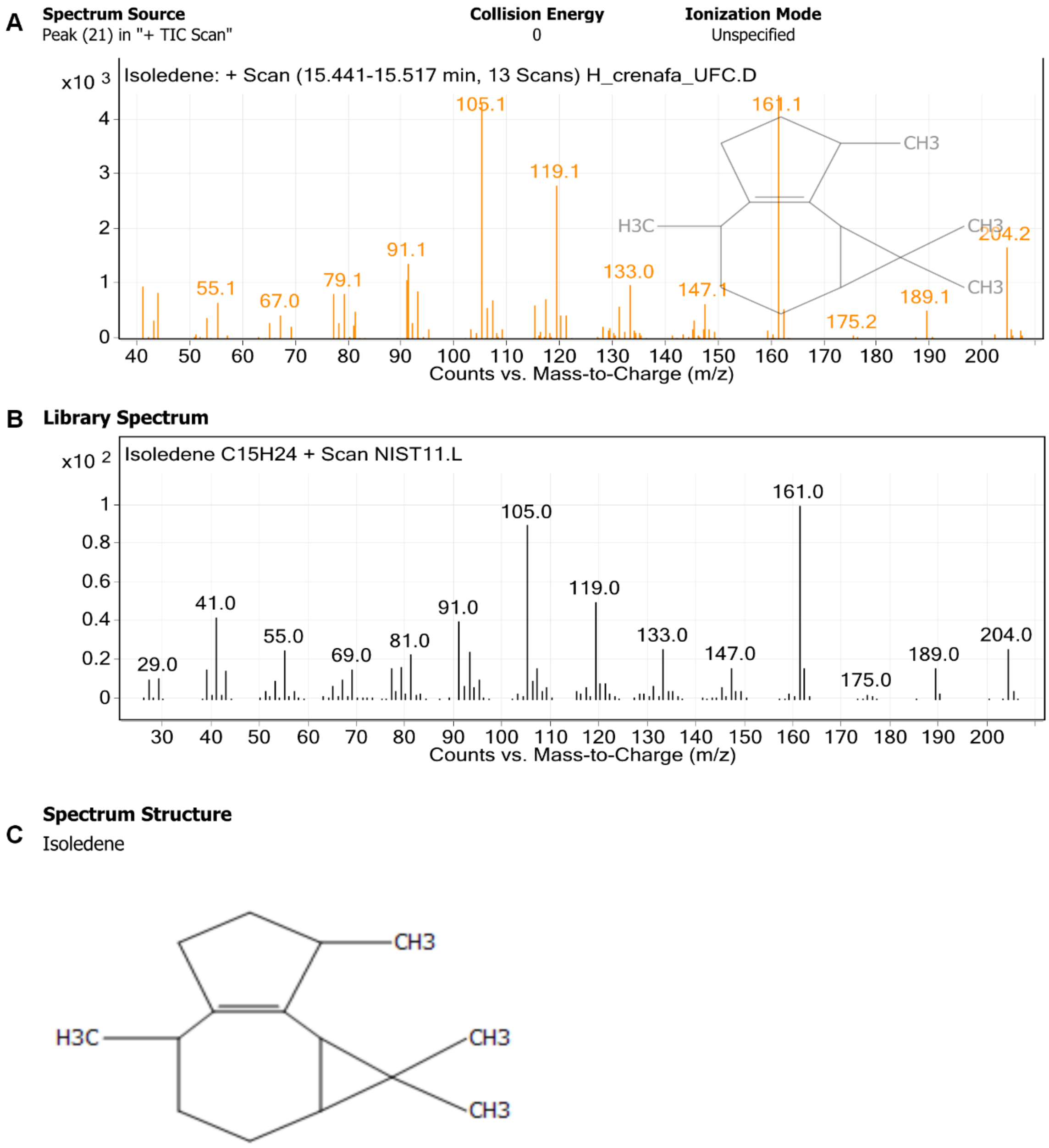

Figure S22 - Mass spectrum of Peak 21 from the TIC chromatogram, assigned to **Isoledene**. (A) Experimental mass spectrum extracted from the chromatographic analysis. (B) Reference mass spectrum from the NIST11 library used for identification. (C) Chemical structure of the identified compound.

FIGURE S23

A **Spectrum Source** Peak (22) in "+ TIC Scan" **Collision Energy** 0 **Ionization Mode** Unspecified

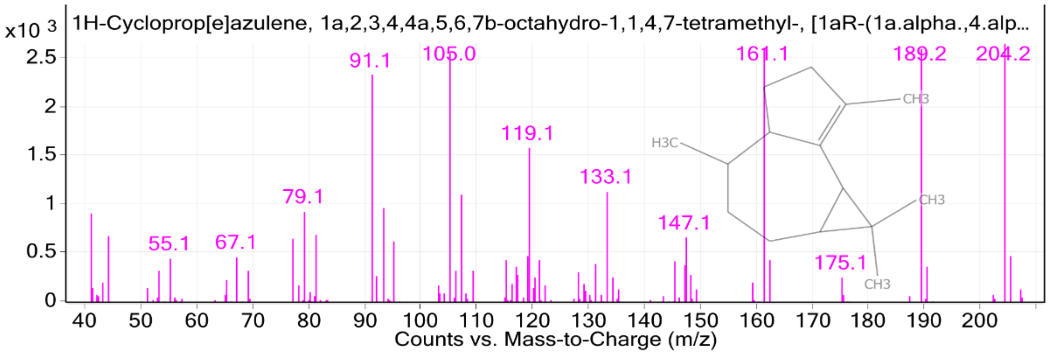

B **Library Spectrum**

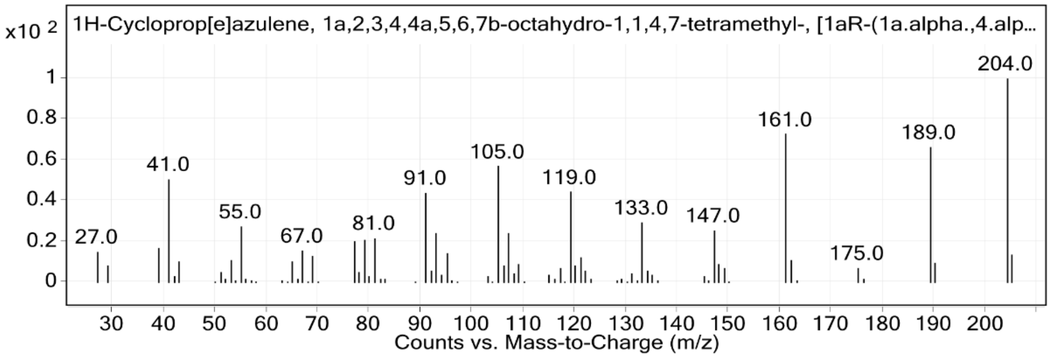

C **Spectrum Structure**

1H-Cycloprop[e]azulene, 1a,2,3,4,4a,5,6,7b-octahydro-1,1,4,7-tetramethyl-, [1aR-(1a.alpha.,4.alpha.,4a.beta.,7b.alpha.)]-

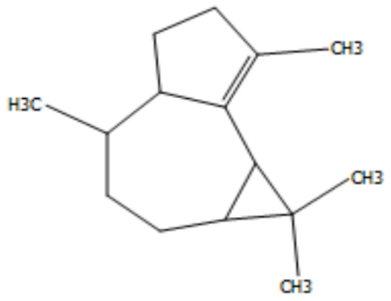

Figure S23 - Mass spectrum of Peak 22 from the TIC chromatogram, assigned to **1H-Cycloprop[e]azulene, 1a,2,3,4,4a,5,6,7b-octahydro-1,1,4,7-tetramethyl-, [1aR-(1a.alpha.,4.alpha.,4a.beta.,7b.alpha.)]-.** (A) Experimental mass spectrum extracted from the chromatographic analysis. (B) Reference mass spectrum from the NIST11 library used for identification. (C) Chemical structure of the identified compound.

FUGURE S24

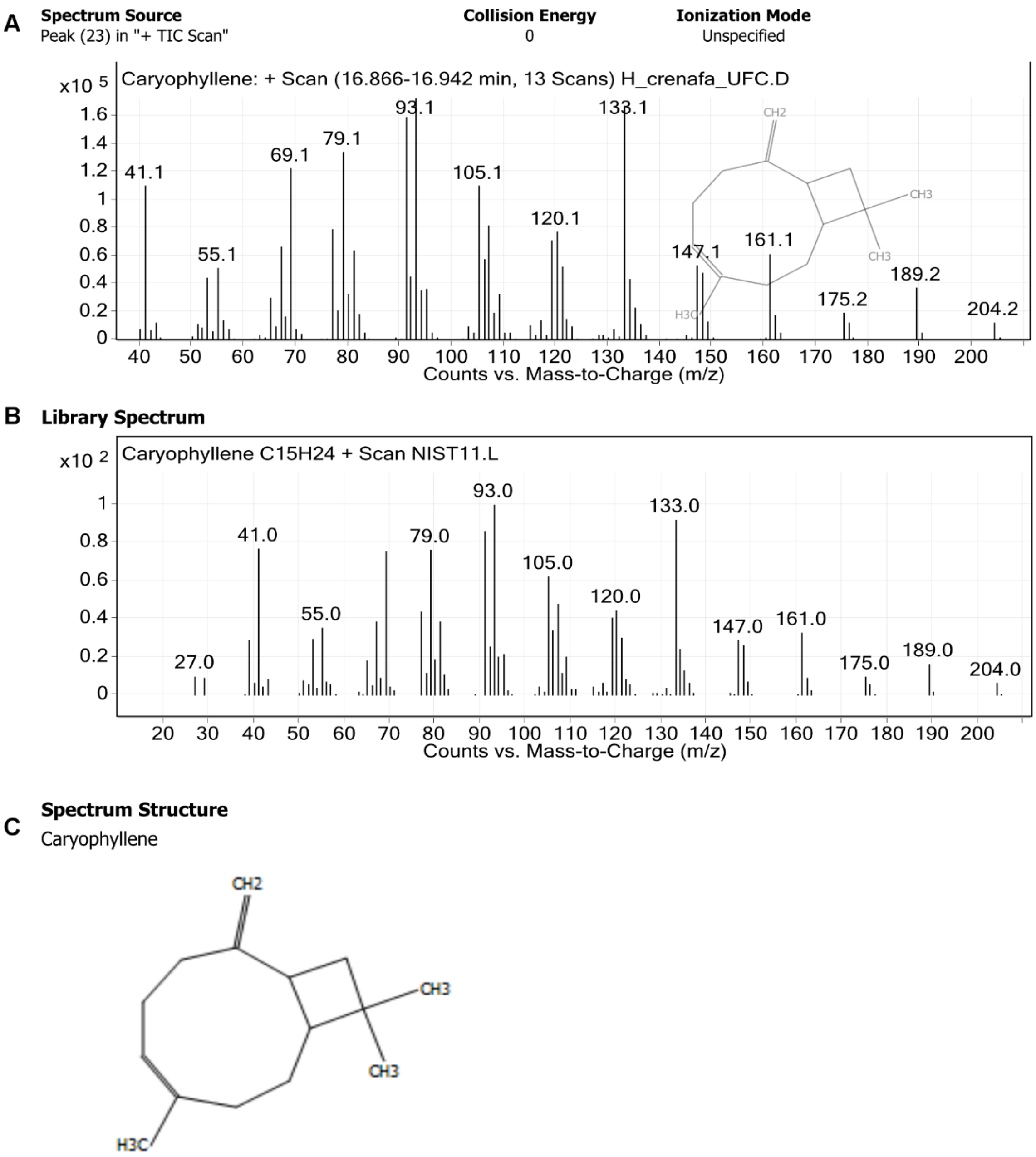

Figure S24 - Mass spectrum of Peak 23 from the TIC chromatogram, assigned to **Caryophyllene.** (A) Experimental mass spectrum extracted from the chromatographic analysis. (B) Reference mass spectrum from the NIST11 library used for identification. (C) Chemical structure of the identified compound.

FIGURE S25

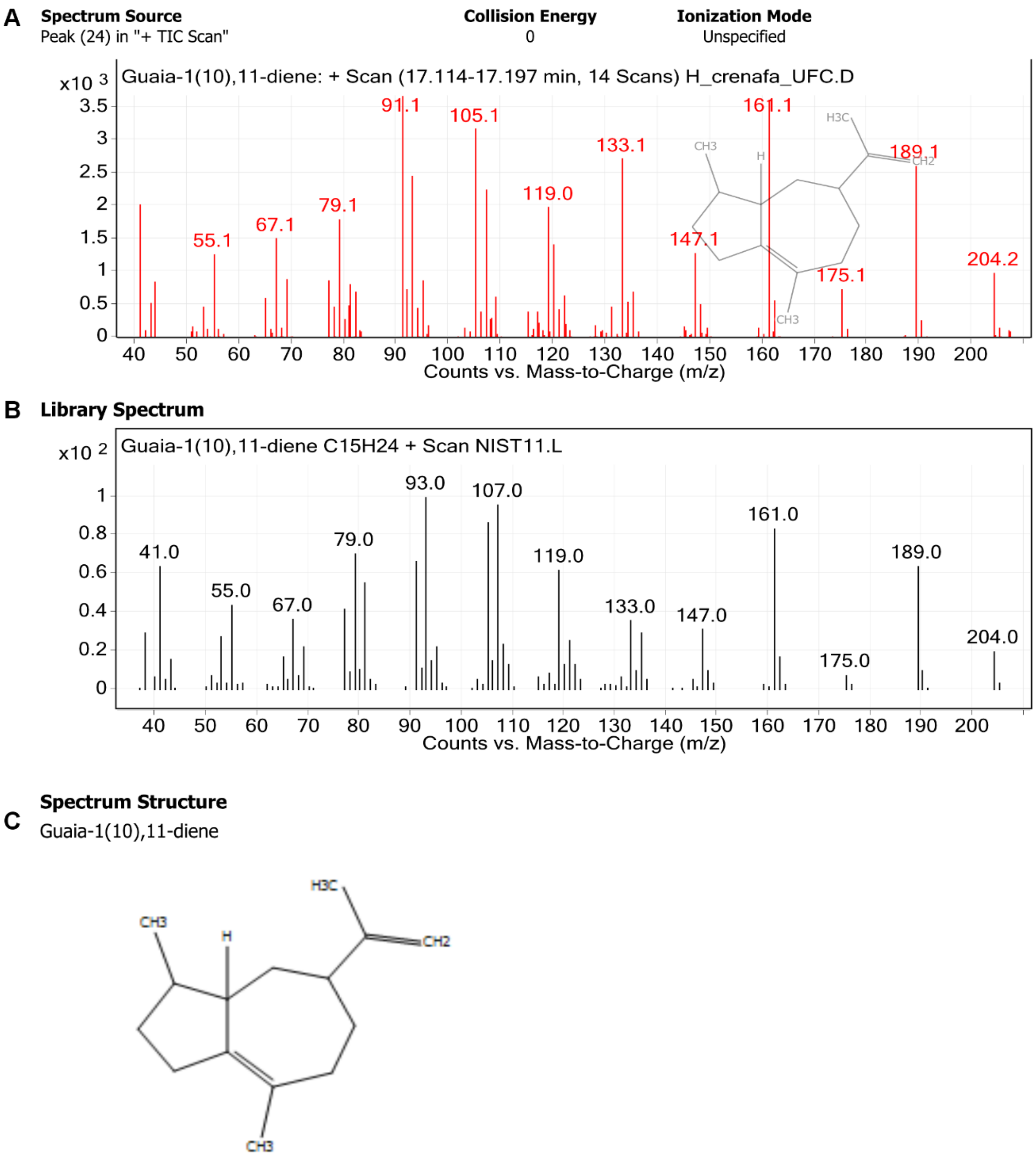

Figure S25 - Mass spectrum of Peak 24 from the TIC chromatogram, assigned to **Guaia-1(10), 11-diene.** (A) Experimental mass spectrum extracted from the chromatographic analysis. (B) Reference mass spectrum from the NIST11 library used for identification. (C) Chemical structure of the identified compound.

FIGURE S26

A **Spectrum Source** Peak (25) in "+ TIC Scan" **Collision Energy** 0 **Ionization Mode** Unspecified

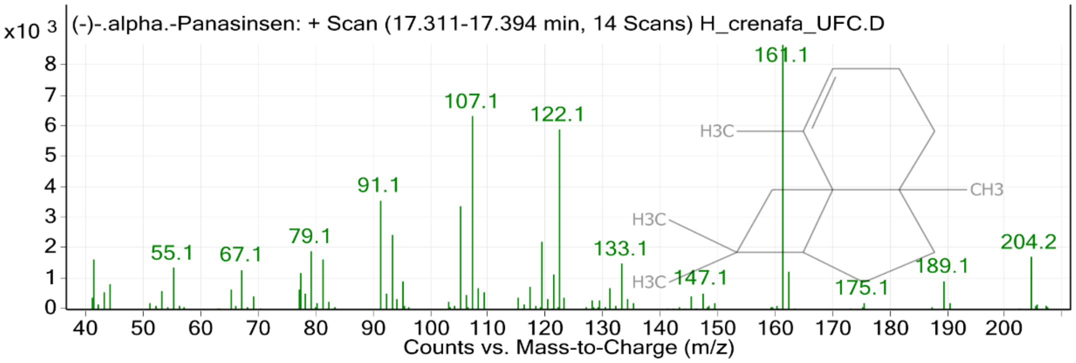

B **Library Spectrum**

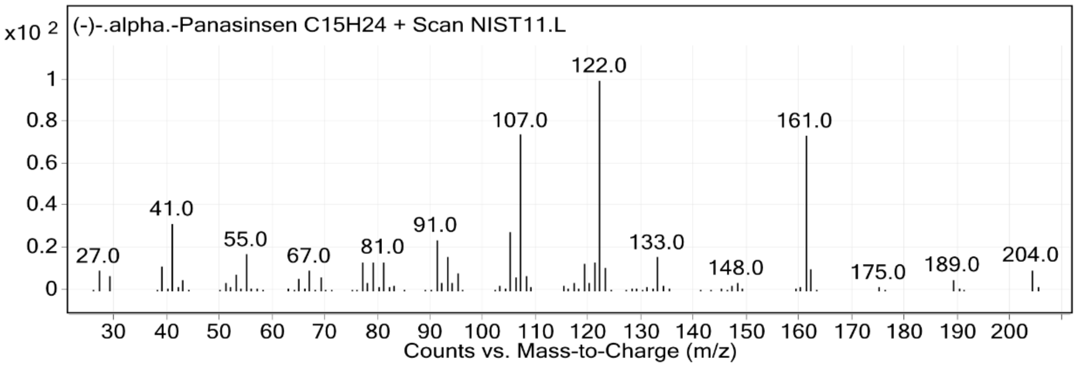

C **Spectrum Structure**
(-)-.alpha.-Panasinsen

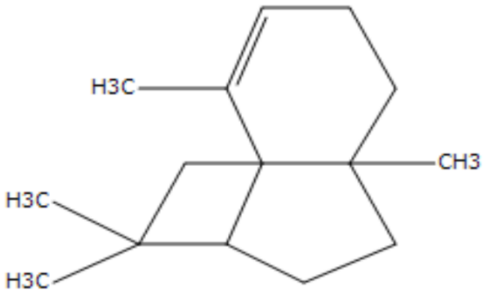

Figure S26 - Mass spectrum of Peak 25 from the TIC chromatogram, assigned to **(-)-.alpha.-Panasinsen.** (A) Experimental mass spectrum extracted from the chromatographic analysis. (B) Reference mass spectrum from the NIST11 library used for identification. (C) Chemical structure of the identified compound.

FIGURE S27

A

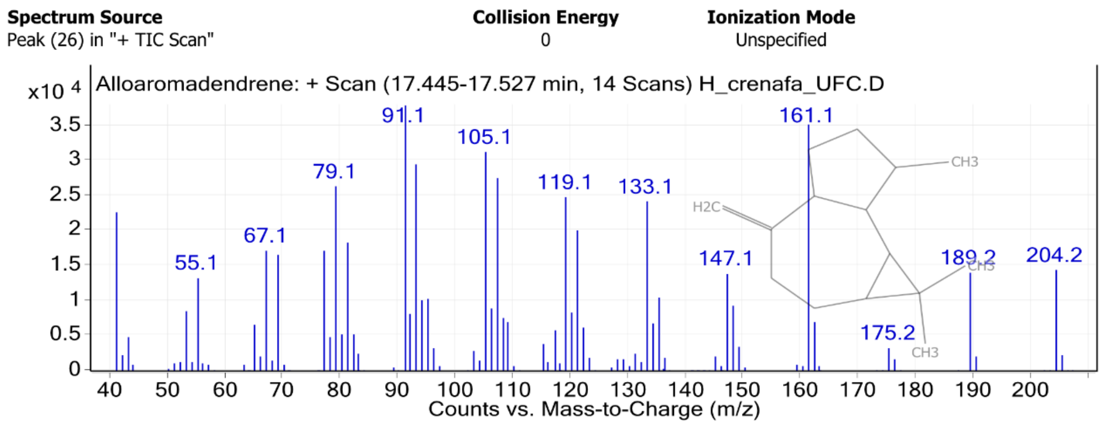

B

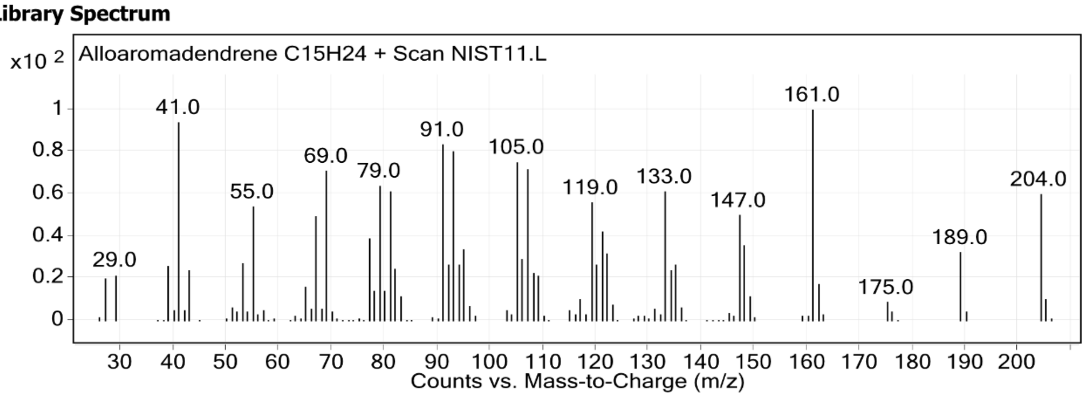

C

Spectrum Structure

Alloaromadendrene

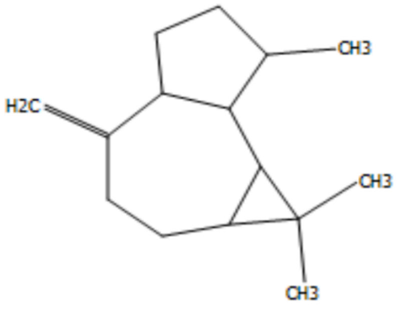

Figure S27 - Mass spectrum of Peak 26 from the TIC chromatogram, assigned to **Alloaromadendrene.** (A) Experimental mass spectrum extracted from the chromatographic analysis. (B) Reference mass spectrum from the NIST11 library used for identification. (C) Chemical structure of the identified compound.

FIGURE S28

A **Spectrum Source** Peak (27) in "+ TIC Scan" — **Collision Energy** 0 — **Ionization Mode** Unspecified

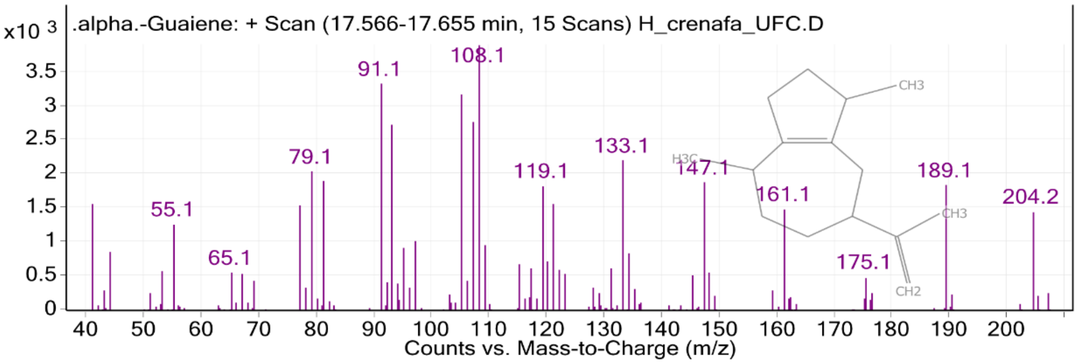

B **Library Spectrum**

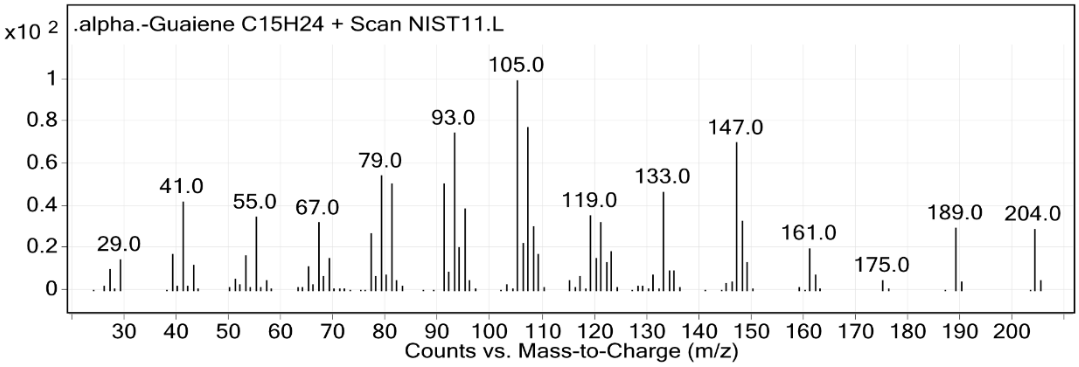

C **Spectrum Structure**

.alpha.-Guaiene

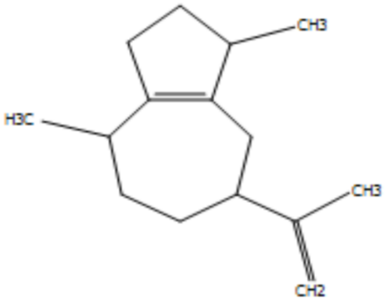

Figure S28 - Mass spectrum of Peak 27 from the TIC chromatogram, assigned to **.alpha.-Guaiene.** (A) Experimental mass spectrum extracted from the chromatographic analysis. (B) Reference mass spectrum from the NIST11 library used for identification. (C) Chemical structure of the identified compound.

FIGURE S29

A **Spectrum Source** Peak (28) in "+ TIC Scan" **Collision Energy** 0 **Ionization Mode** Unspecified

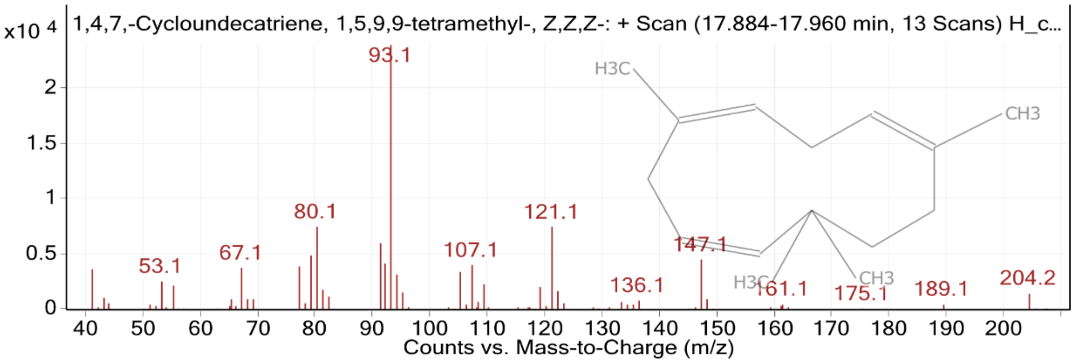

B **Library Spectrum**

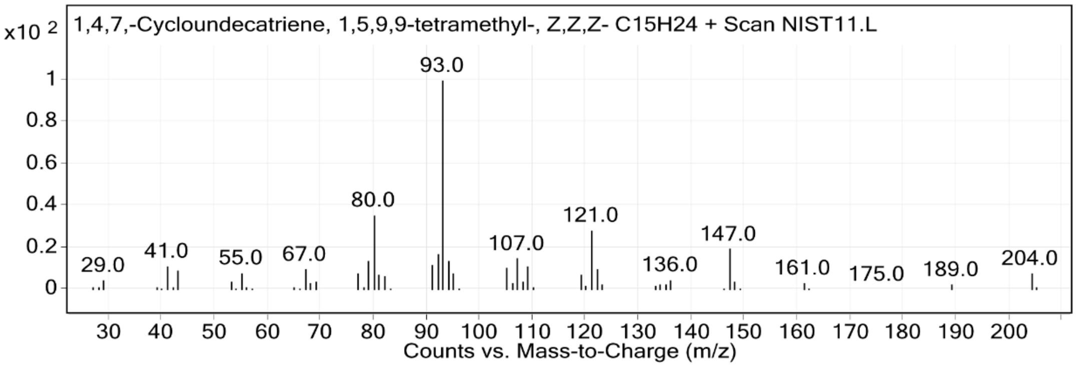

C **Spectrum Structure**
1,4,7,-Cycloundecatriene, 1,5,9,9-tetramethyl-, Z,Z,Z-

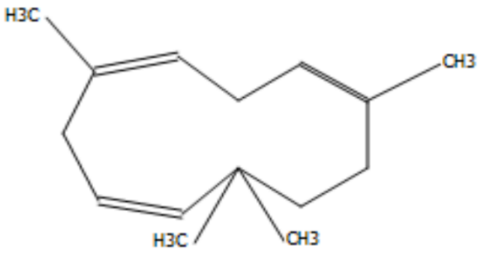

Figure S29 - Mass spectrum of Peak 28 from the TIC chromatogram, assigned to **1,4,7,-Cycloundecatriene, 1,5,9,9-tetramethyl-,Z,Z,Z-.** (A) Experimental mass spectrum extracted from the chromatographic analysis. (B) Reference mass spectrum from the NIST11 library used for identification. (C) Chemical structure of the identified compound.

FIGURE S30

A **Spectrum Source** Peak (29) in "+ TIC Scan" | **Collision Energy** 0 | **Ionization Mode** Unspecified

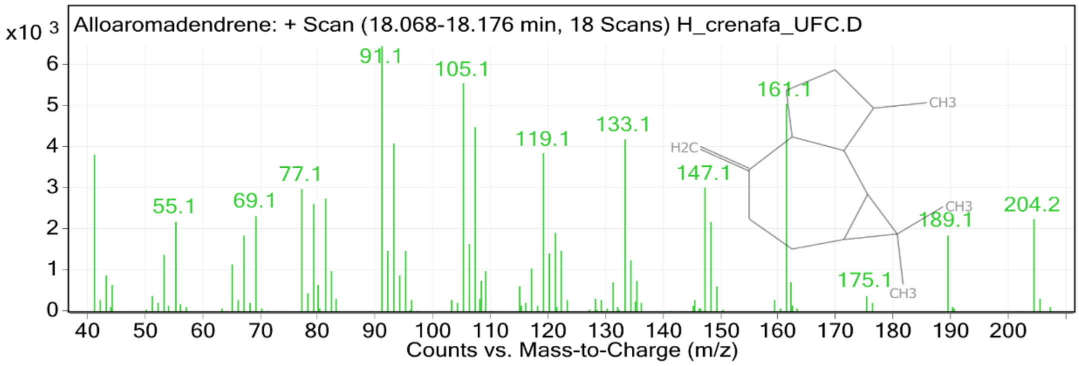

B **Library Spectrum**

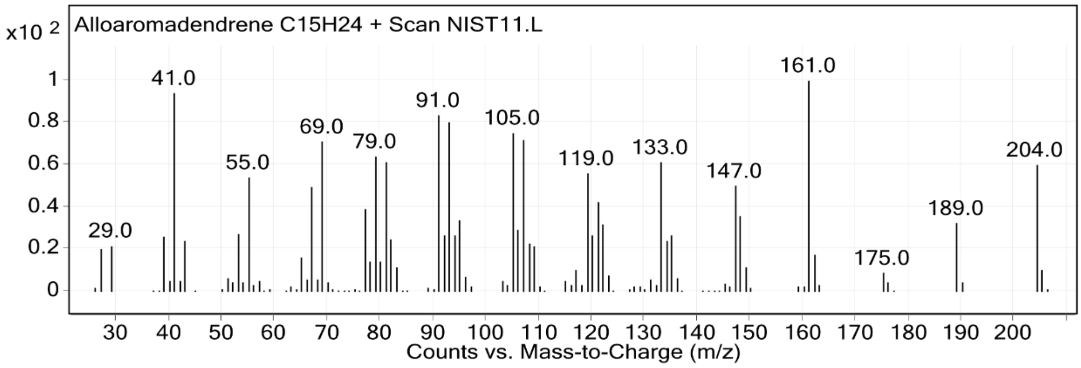

C **Spectrum Structure**
Alloaromadendrene

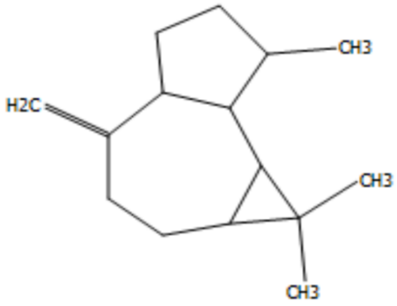

Figure S30 - Mass spectrum of Peak 29 from the TIC chromatogram, assigned to **Alloaromadendrene,** for the second time**.** (A) Experimental mass spectrum extracted from the chromatographic analysis. (B) Reference mass spectrum from the NIST11 library used for identification. (C) Chemical structure of the identified compound.

FIGURE S31

A **Spectrum Source** Peak (30) in "+ TIC Scan" | **Collision Energy** 0 | **Ionization Mode** Unspecified

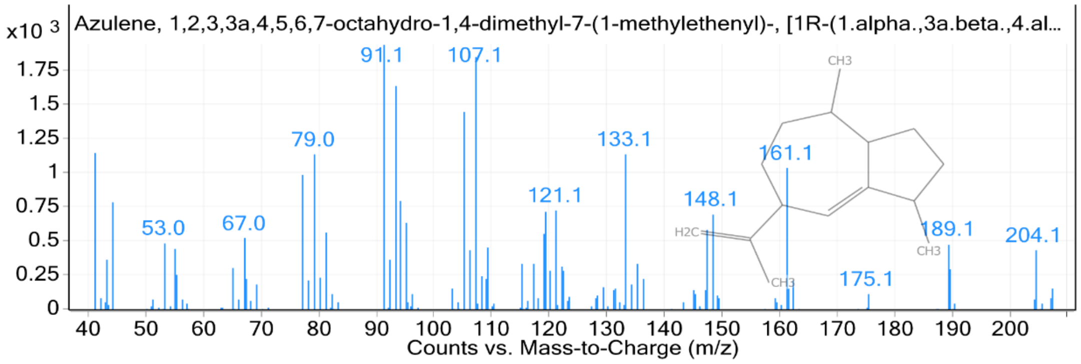

B **Library Spectrum**

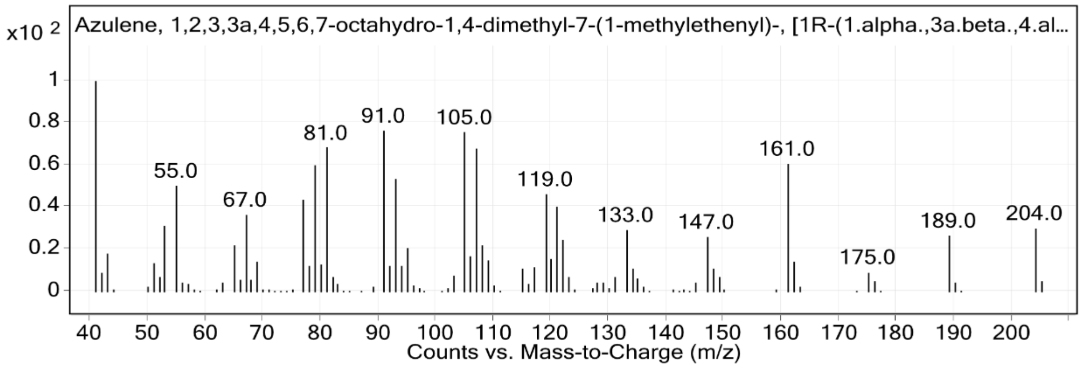

C **Spectrum Structure**
Azulene, 1,2,3,3a,4,5,6,7-octahydro-1,4-dimethyl-7-(1-methylethenyl)-, [1R-(1.alpha.,3a.beta.,4.alpha.,7.beta.)]-

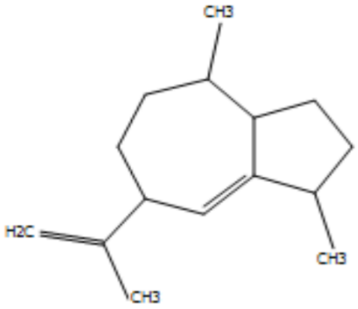

Figure S31 - Mass spectrum of Peak 30 from the TIC chromatogram, assigned to **Azulene, 1,2,3,3a,4,5,6,7-octahydrol-1,4-dimethyl-7-(1-methylethenyl)-, [1R-(1.alpha., 3a.beta., 4.alpha.,7.beta.)]-.** (A) Experimental mass spectrum extracted from the chromatographic analysis. (B) Reference mass spectrum from the NIST11 library used for identification. (C) Chemical structure of the identified compound.

FIGURE S32

A

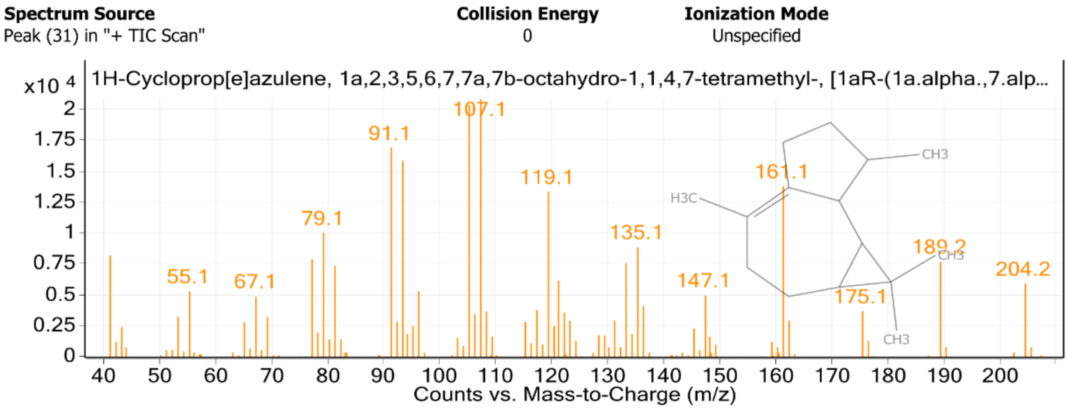

B

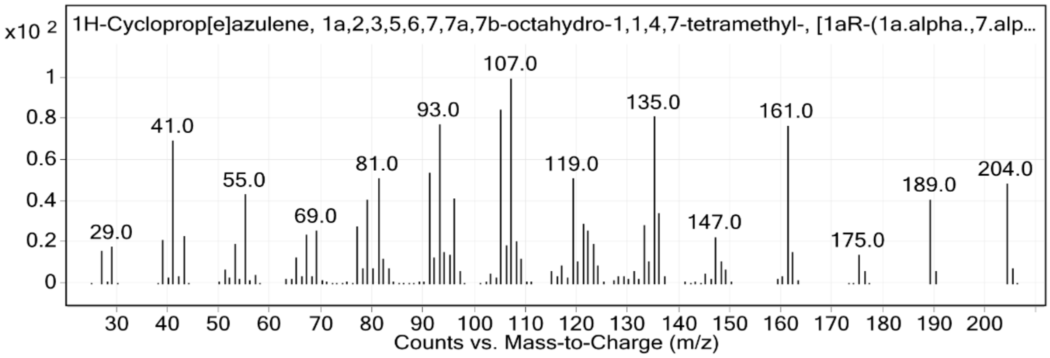

Spectrum Structure

C

1H-Cycloprop[e]azulene, 1a,2,3,5,6,7,7a,7b-octahydro-1,1,4,7-tetramethyl-, [1aR-(1a.alpha.,7.alpha.,7a.beta.,7b.alpha.)]-

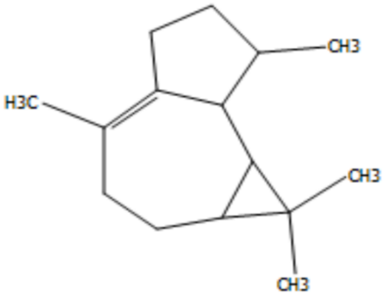

Figure S32 - Mass spectrum of Peak 31 from the TIC chromatogram, assigned to **1H-Cycloprop[e]azulene, 1a,2,3,4,4a,5,6,7b-octahydro-1,1,4,7-tetramethyl-, [1aR-(1a.alpha.,4.alpha.,4a.beta.,7b.alpha.)]-,** for the second time. (A) Experimental mass spectrum extracted from the chromatographic analysis. (B) Reference mass spectrum from the NIST11 library used for identification. (C) Chemical structure of the identified compound.

FIGURE S33

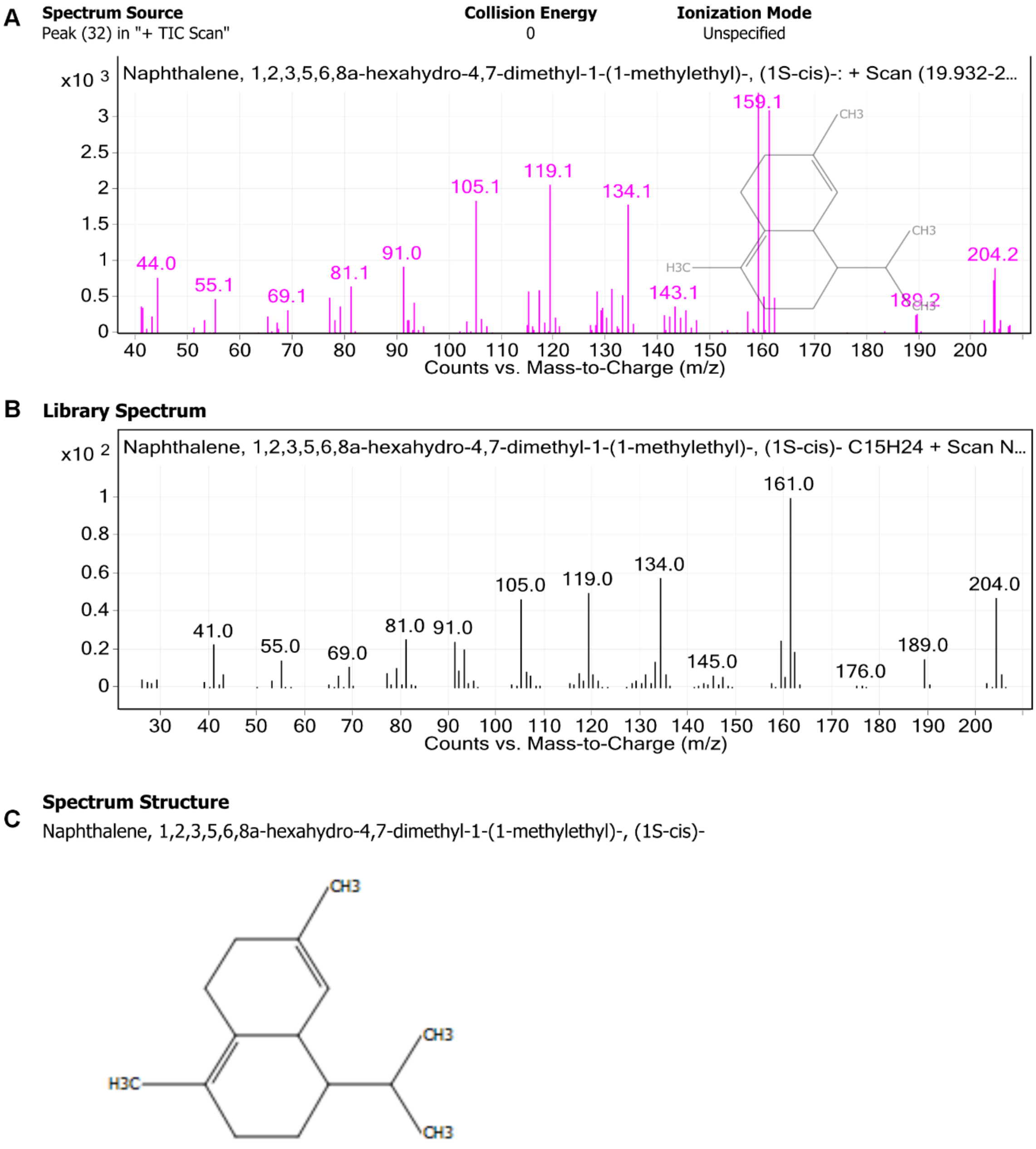

Figure S33 - Mass spectrum of Peak 32 from the TIC chromatogram, assigned to **Naphthalene, 1,2,3,5,6,8a-hexahydrol-4,7-dimethyl-1-(1-methylethyl)-,(1S-cis)-.** (A) Experimental mass spectrum extracted from the chromatographic analysis. (B) Reference mass spectrum from the NIST11 library used for identification. (C) Chemical structure of the identified compound.

FIGURE S34

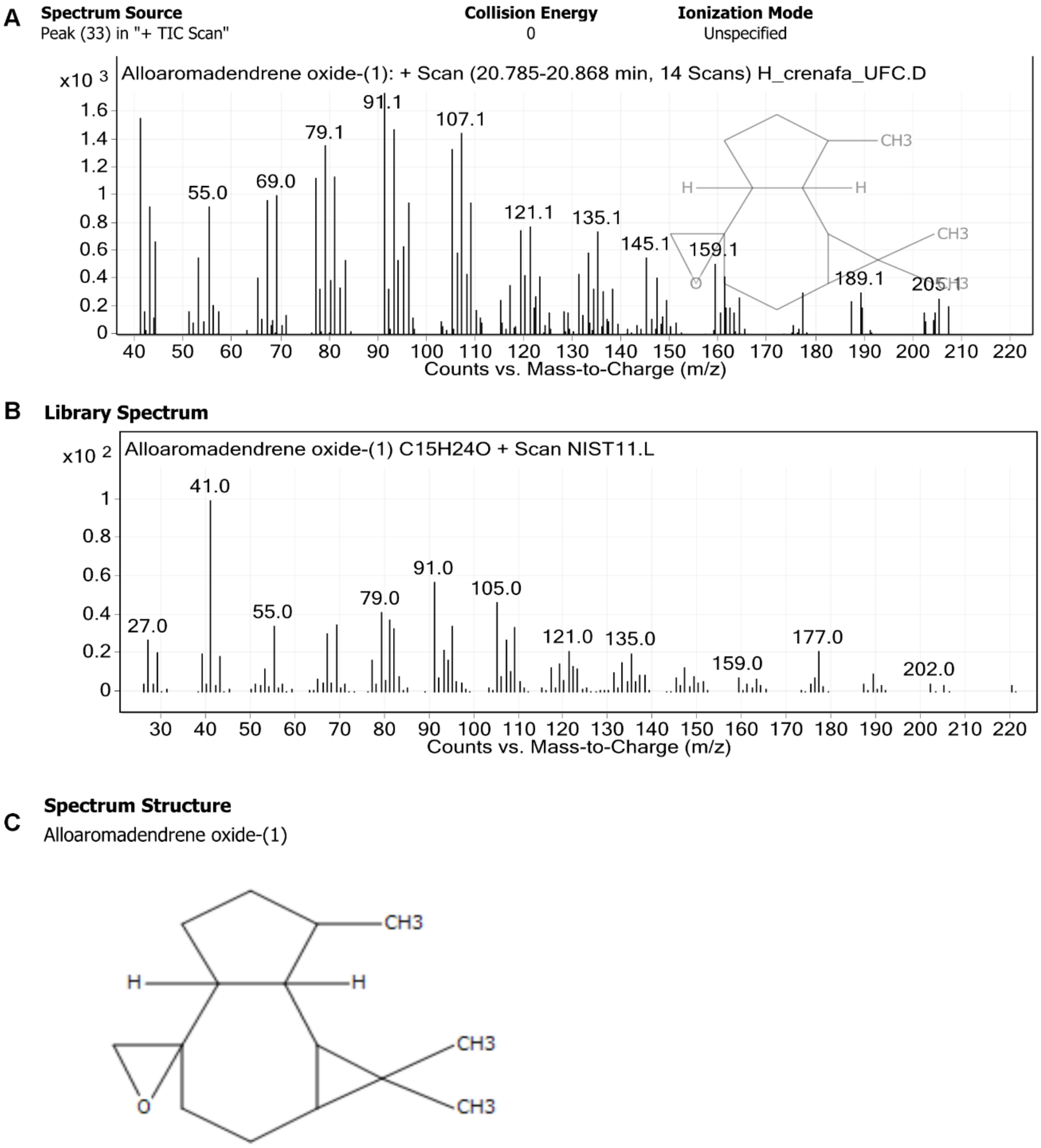

Figure S34 - Mass spectrum of Peak 33 from the TIC chromatogram, assigned to **Alloaromadendrene oxide-(1).** (A) Experimental mass spectrum extracted from the chromatographic analysis. (B) Reference mass spectrum from the NIST11 library used for identification. (C) Chemical structure of the identified compound.

FIGURE S35

A **Spectrum Source** Peak (34) in "+ TIC Scan" | **Collision Energy** 0 | **Ionization Mode** Unspecified

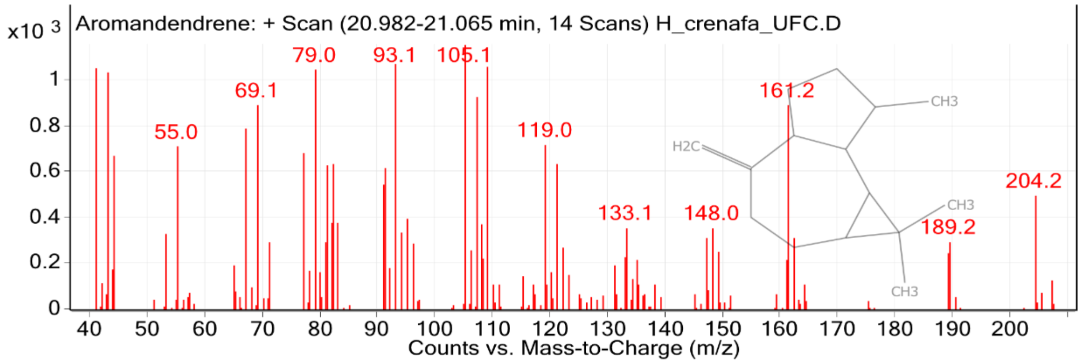

B **Library Spectrum**

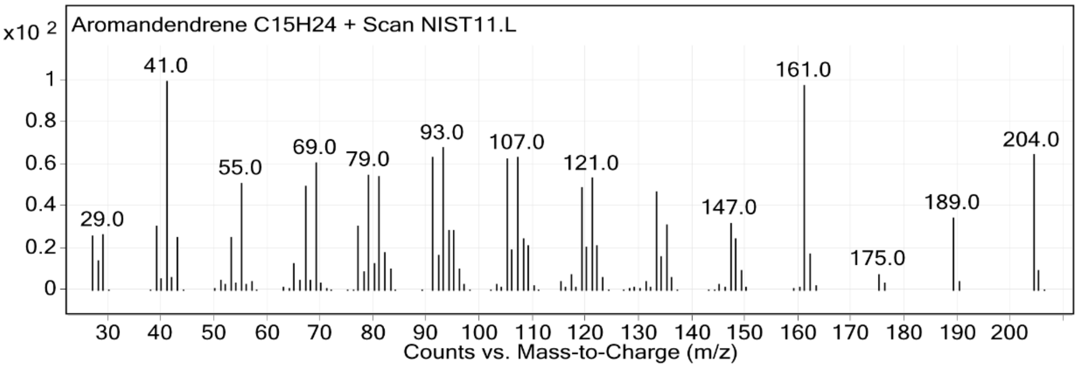

C **Spectrum Structure**
Aromandendrene

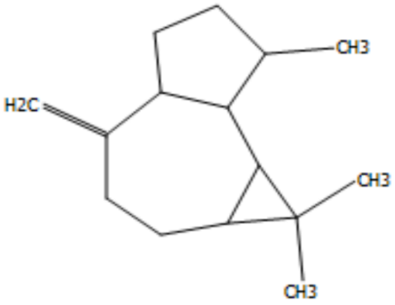

Figure S35 - Mass spectrum of Peak 34 from the TIC chromatogram, assigned to **Alloaromadendrene**, for the second time. (A) Experimental mass spectrum extracted from the chromatographic analysis. (B) Reference mass spectrum from the NIST11 library used for identification. (C) Chemical structure of the identified compound.

FIGURE S36

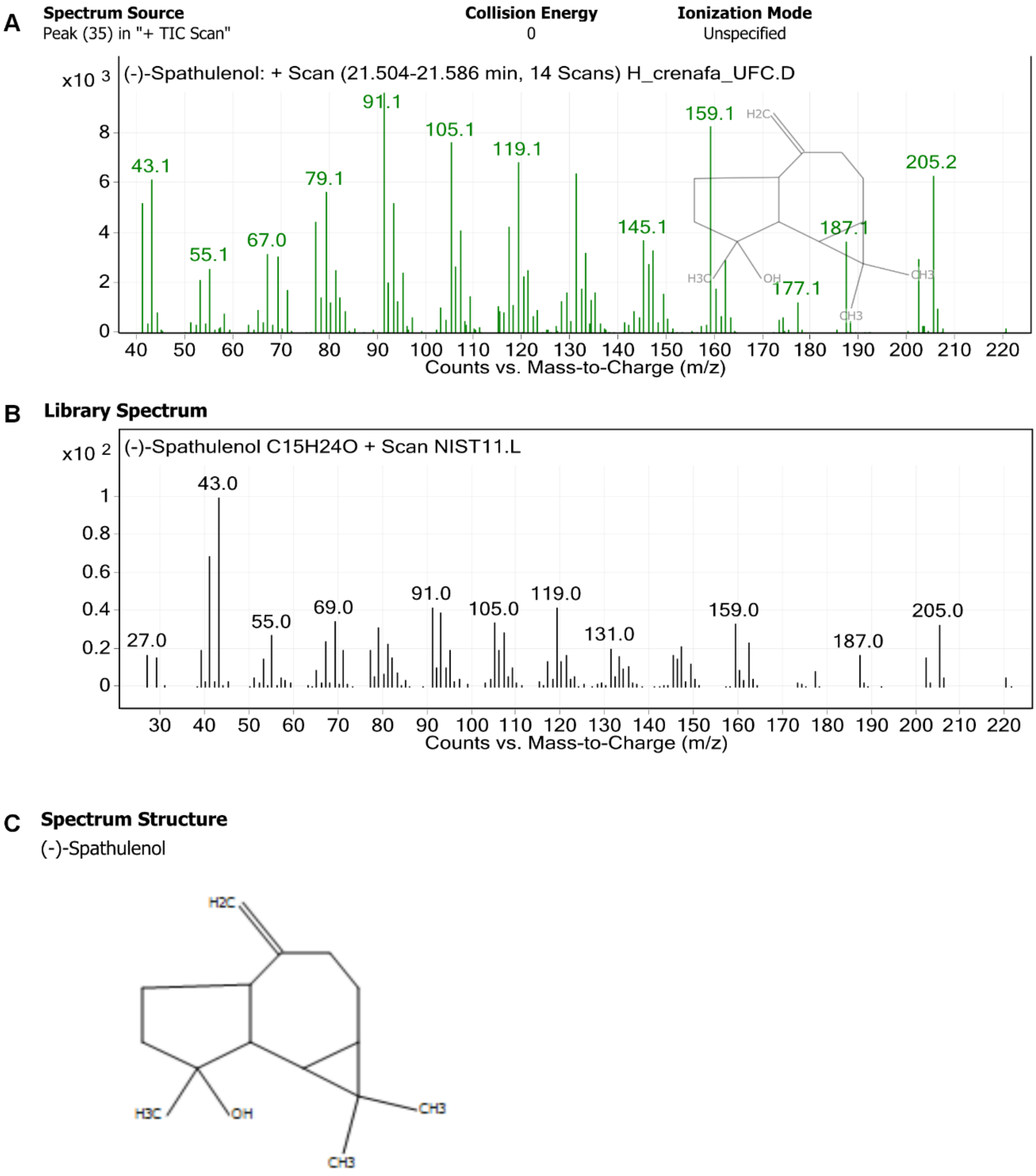

Figure S36 - Mass spectrum of Peak 35 from the TIC chromatogram, assigned to **(-)-Spathulenol.** (A) Experimental mass spectrum extracted from the chromatographic analysis. (B) Reference mass spectrum from the NIST11 library used for identification. (C) Chemical structure of the identified compound.

FIGURE S37

A **Spectrum Source** Peak (36) in "+ TIC Scan" | **Collision Energy** 0 | **Ionization Mode** Unspecified

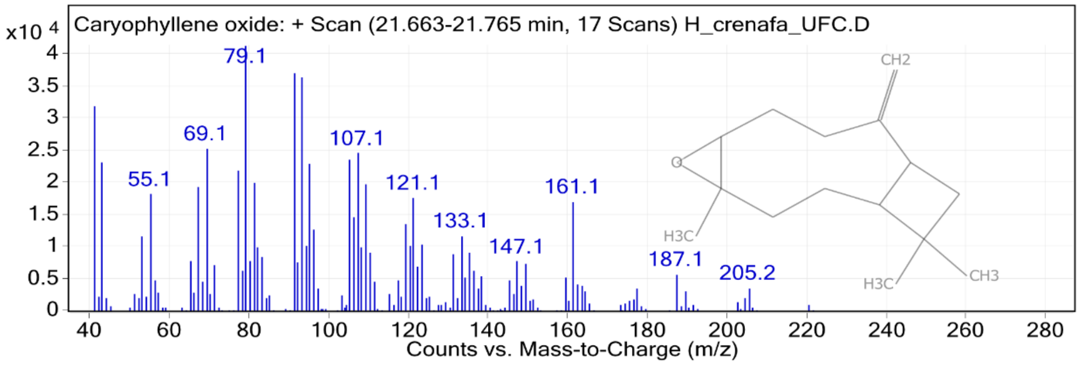

B **Library Spectrum**

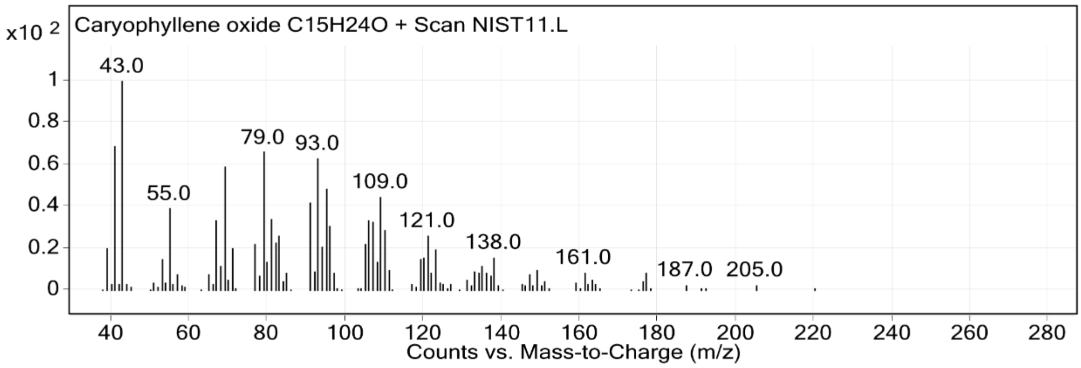

C **Spectrum Structure**
Caryophyllene oxide

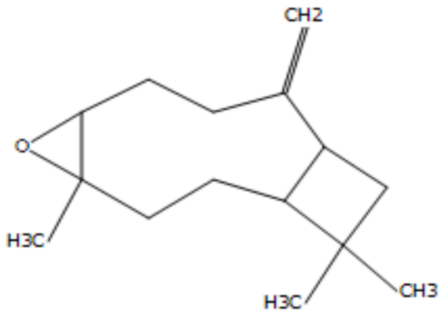

Figure S37 - Mass spectrum of Peak 36 from the TIC chromatogram, assigned to **Caryophyllene oxide.** (A) Experimental mass spectrum extracted from the chromatographic analysis. (B) Reference mass spectrum from the NIST11 library used for identification. (C) Chemical structure of the identified compound.

FIGURE S38

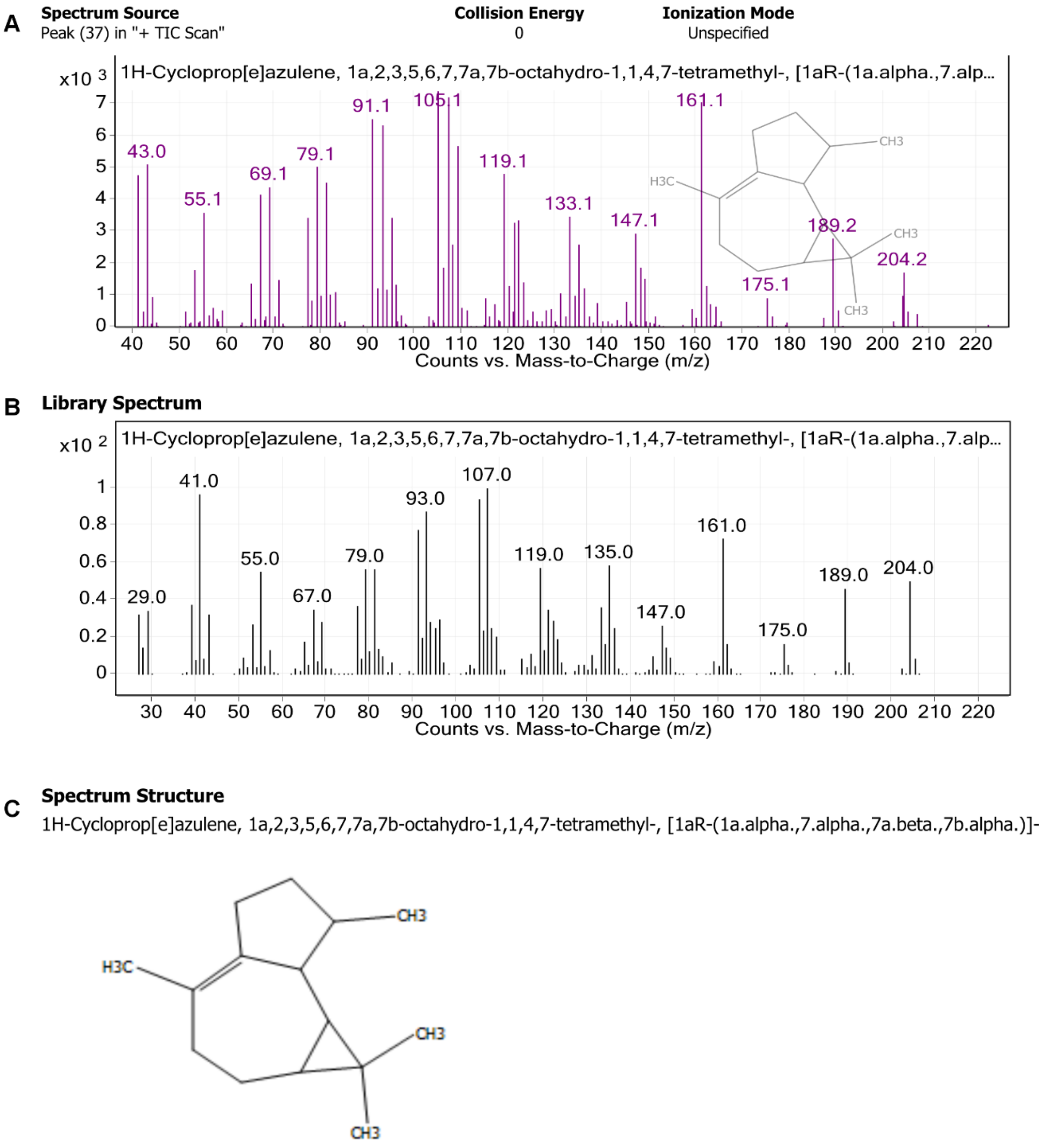

Figure S38 - Mass spectrum of Peak 37 from the TIC chromatogram, assigned to **1H-Cycloprop[e]azulene, 1a,2,3,56,7,7a,7b-octahydro-1,1,4,7-tetramethyl-, [1aR-(1a.alpha.,7.alpha.,7a.beta.,7b.alpha.)]-.** (A) Experimental mass spectrum extracted from the chromatographic analysis. (B) Reference mass spectrum from the NIST11 library used for identification. (C) Chemical structure of the identified compound.

FIGURE S39

A

Spectrum Source Peak (38) in "+ TIC Scan" | **Collision Energy** 0 | **Ionization Mode** Unspecified

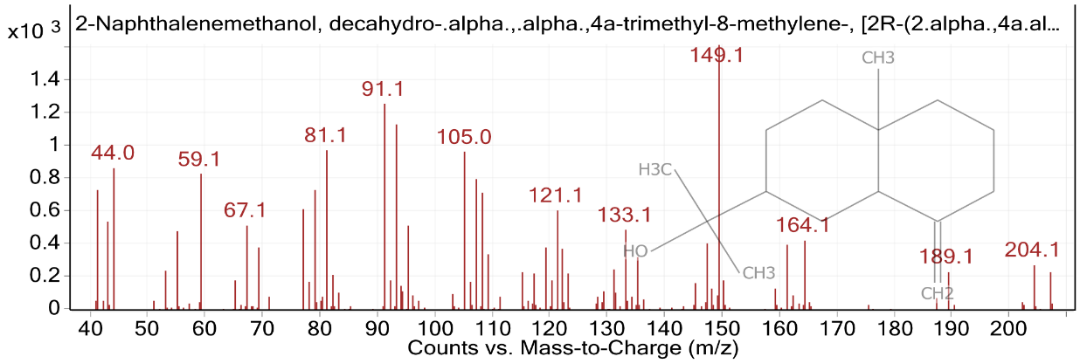

B **Library Spectrum**

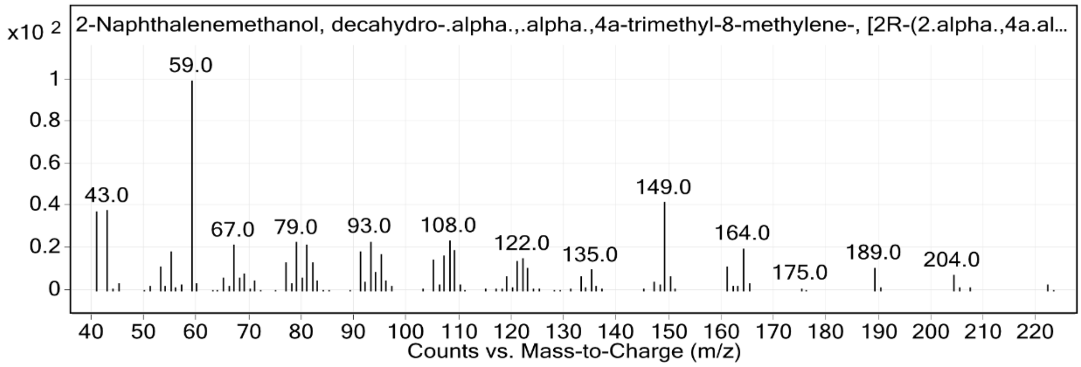

C **Spectrum Structure**

2-Naphthalenemethanol, decahydro-.alpha.,.alpha.,4a-trimethyl-8-methylene-, [2R-(2.alpha.,4a.alpha.,8a.beta.)]-

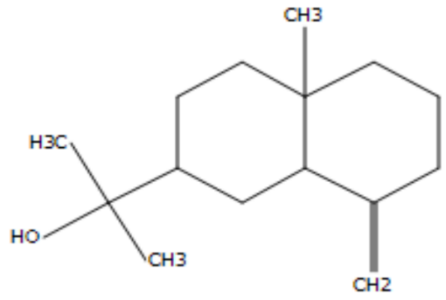

Figure S39 - Mass spectrum of Peak 38 from the TIC chromatogram, assigned to **2-Naphthalenemethanol. Decahydro-.alpha.,.alpha.,4a-trimethy-8-methylene-,[2R-(2.alpha.,4a.alpha.,8a.beta.)]-.** (A) Experimental mass spectrum extracted from the chromatographic analysis. (B) Reference mass spectrum from the NIST11 library used for identification. (C) Chemical structure of the identified compound.

FIGURE S40

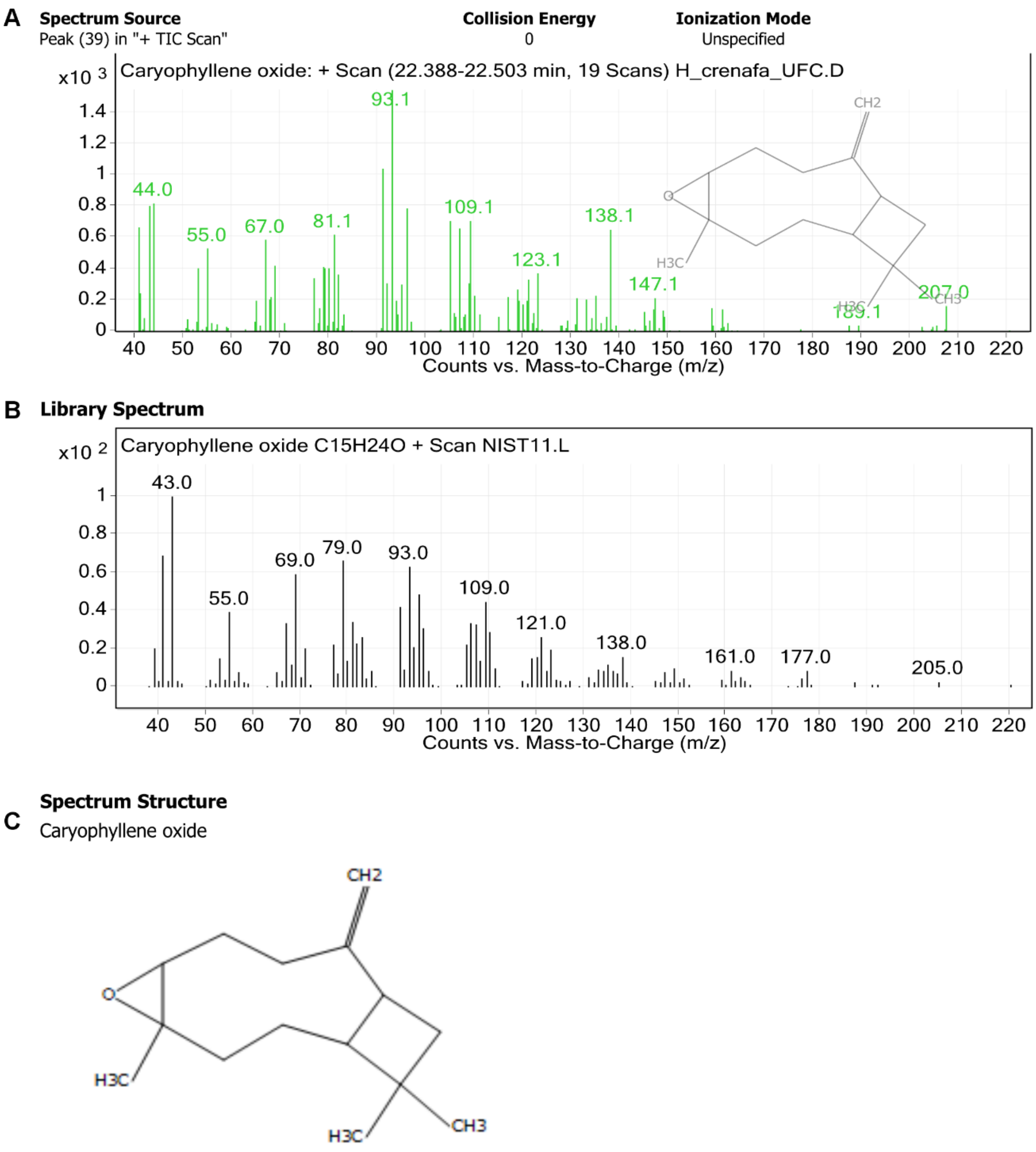

Figure S40 - Mass spectrum of Peak 39 from the TIC chromatogram, assigned to **Caryophyllene**, for the second time. (A) Experimental mass spectrum extracted from the chromatographic analysis. (B) Reference mass spectrum from the NIST11 library used for identification. (C) Chemical structure of the identified compound.

FIGURE S41

A

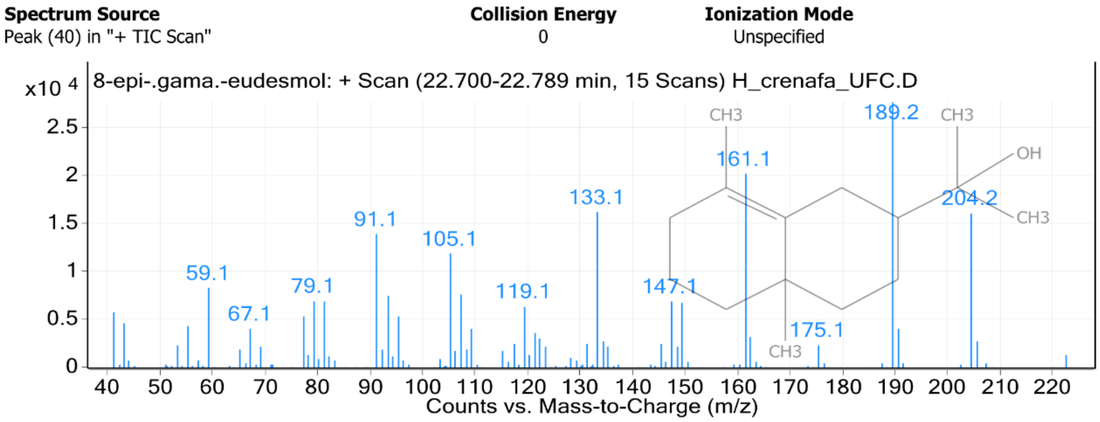

B

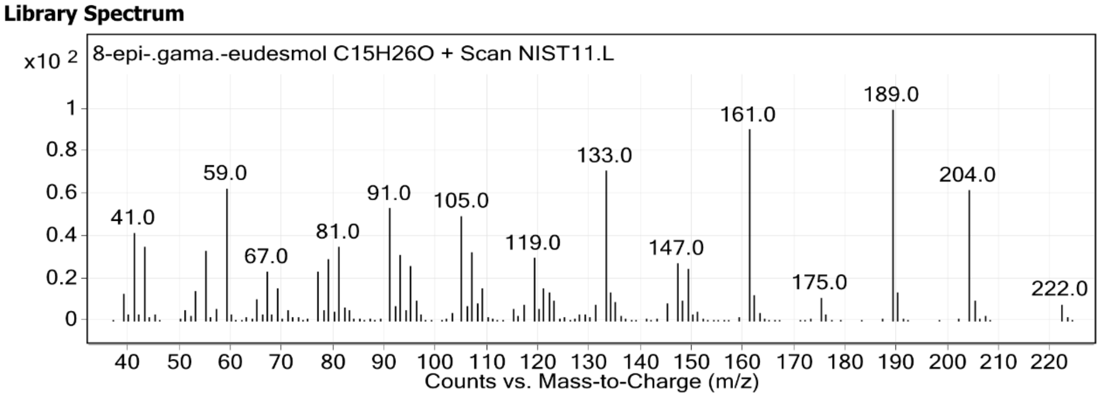

C

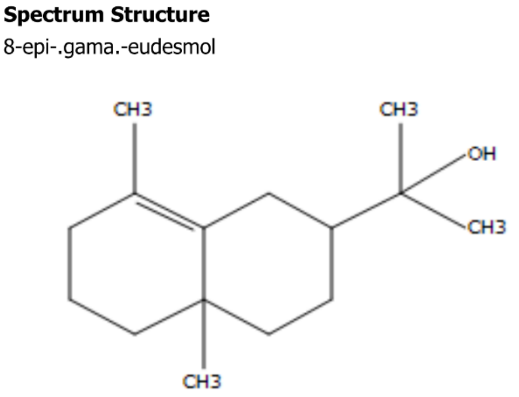

Figure S41 - Mass spectrum of Peak 40 from the TIC chromatogram, assigned to **8-epi-.gama.-eudesmol.** (A) Experimental mass spectrum extracted from the chromatographic analysis. (B) Reference mass spectrum from the NIST11 library used for identification. (C) Chemical structure of the identified compound.

FIGURE S42

A **Spectrum Source** Peak (41) in "+ TIC Scan" — **Collision Energy** 0 — **Ionization Mode** Unspecified

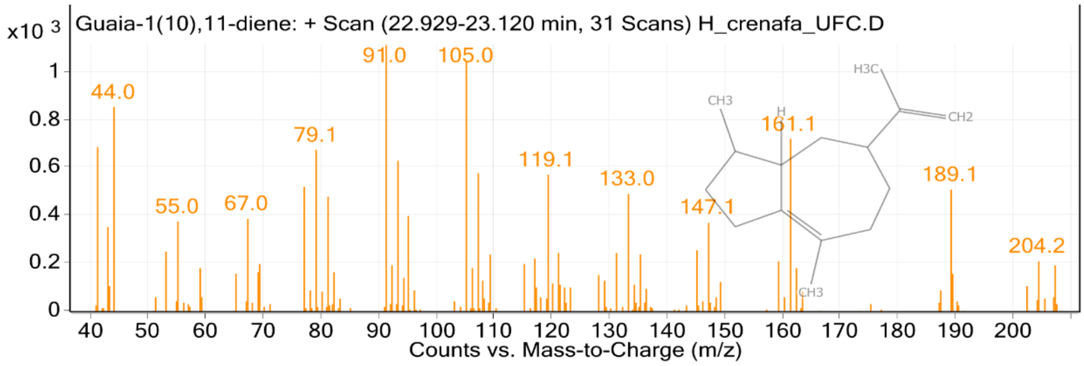

B **Library Spectrum**

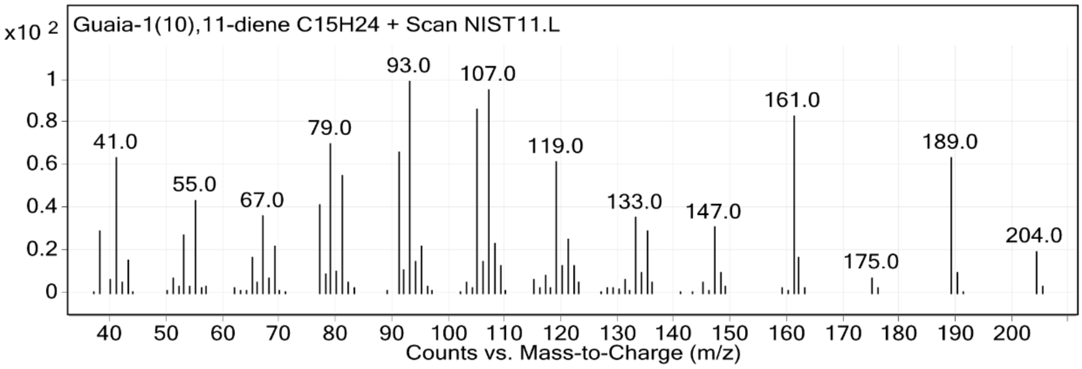

C **Spectrum Structure**
Guaia-1(10),11-diene

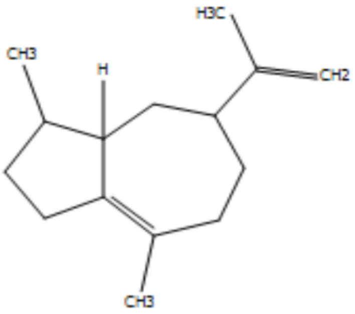

Figure S42 - Mass spectrum of Peak 41 from the TIC chromatogram, assigned to **Guaia-1(10),11-diene.** (A) Experimental mass spectrum extracted from the chromatographic analysis. (B) Reference mass spectrum from the NIST11 library used for identification. (C) Chemical structure of the identified compound.

FIGURE S43

A **Spectrum Source** Peak (42) in "+ TIC Scan" — **Collision Energy** 0 — **Ionization Mode** Unspecified

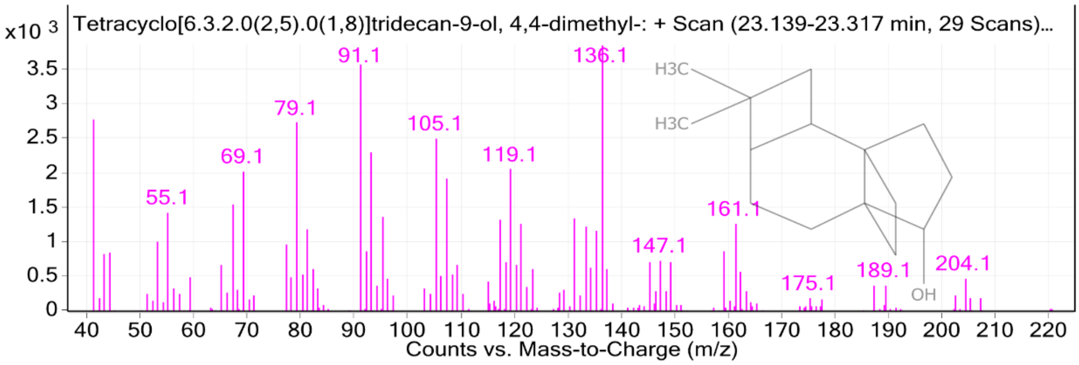

B **Library Spectrum**

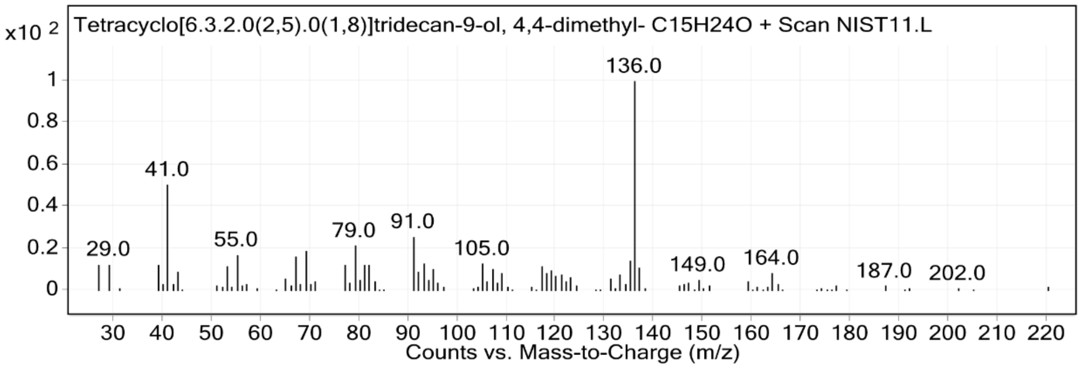

C **Spectrum Structure**

Tetracyclo[6.3.2.0(2,5).0(1,8)]tridecan-9-ol, 4,4-dimethyl-

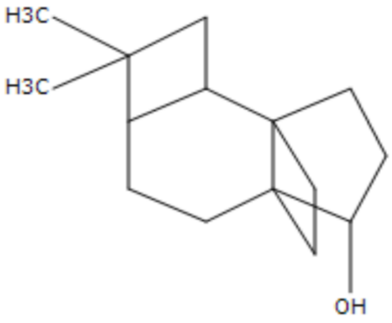

Figure S43 - Mass spectrum of Peak 42 from the TIC chromatogram, assigned to **Tetracyclo[6.3.2.0(2,5).0(1,8)]tridecan-9-ol, 4,4-dimethyl-.** (A) Experimental mass spectrum extracted from the chromatographic analysis. (B) Reference mass spectrum from the NIST11 library used for identification. (C) Chemical structure of the identified compound.

FIGURE S44

A

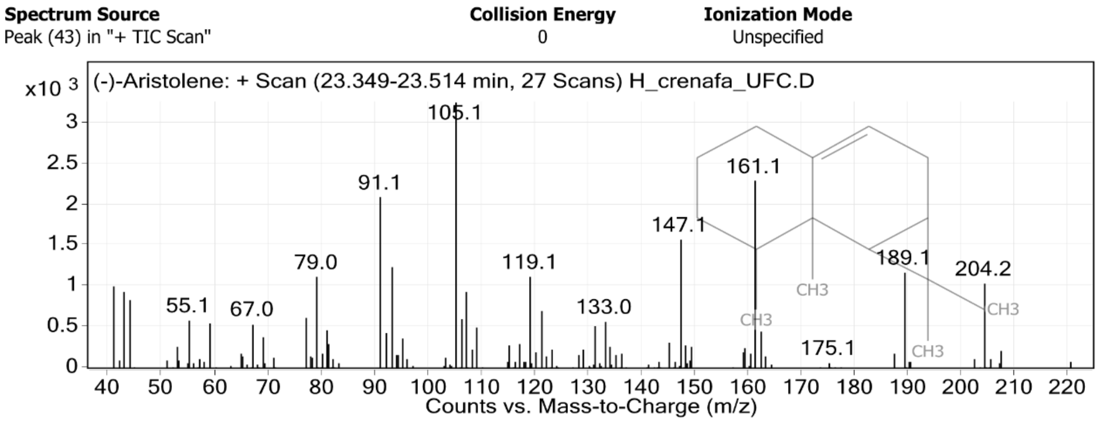

B

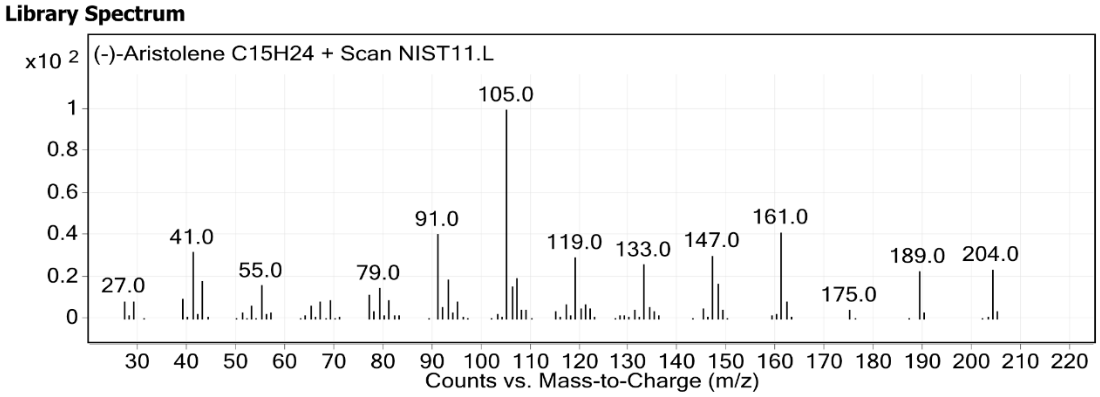

C

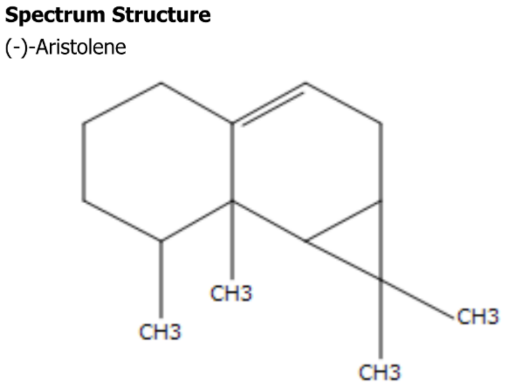

Figure S44 - Mass spectrum of Peak 43 from the TIC chromatogram, assigned to **(-)-Aristolene.** (A) Experimental mass spectrum extracted from the chromatographic analysis. (B) Reference mass spectrum from the NIST11 library used for identification. (C) Chemical structure of the identified compound.

FIGURE S45

A **Spectrum Source** Peak (44) in "+ TIC Scan" — **Collision Energy** 0 — **Ionization Mode** Unspecified

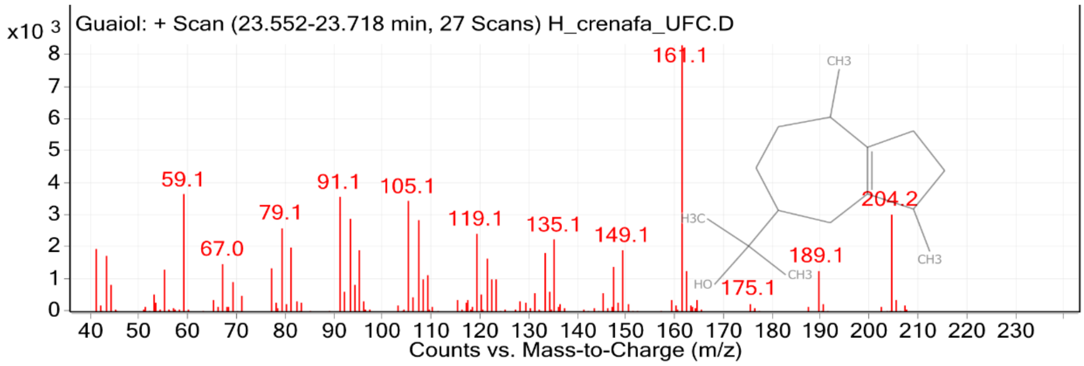

B **Library Spectrum**

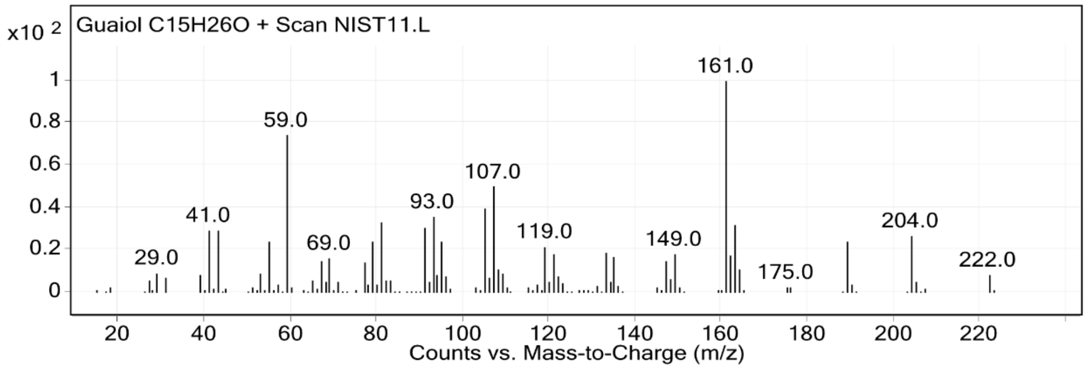

C

Spectrum Structure

Guaiol

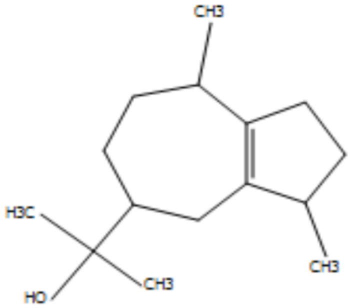

Figure S45 - Mass spectrum of Peak 44 from the TIC chromatogram, assigned to **Guaiol.** (A) Experimental mass spectrum extracted from the chromatographic analysis. (B) Reference mass spectrum from the NIST11 library used for identification. (C) Chemical structure of the identified compound.

FIGURE S46

A **Spectrum Source** Peak (45) in "+ TIC Scan" — **Collision Energy** 0 — **Ionization Mode** Unspecified

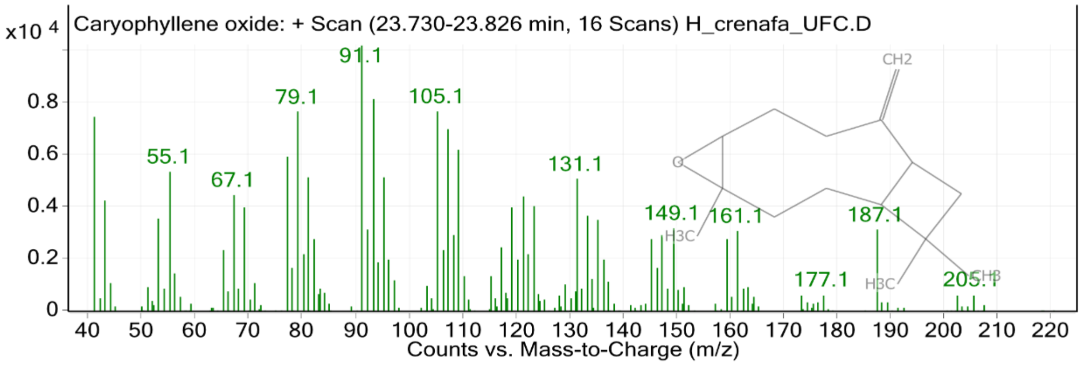

B **Library Spectrum**

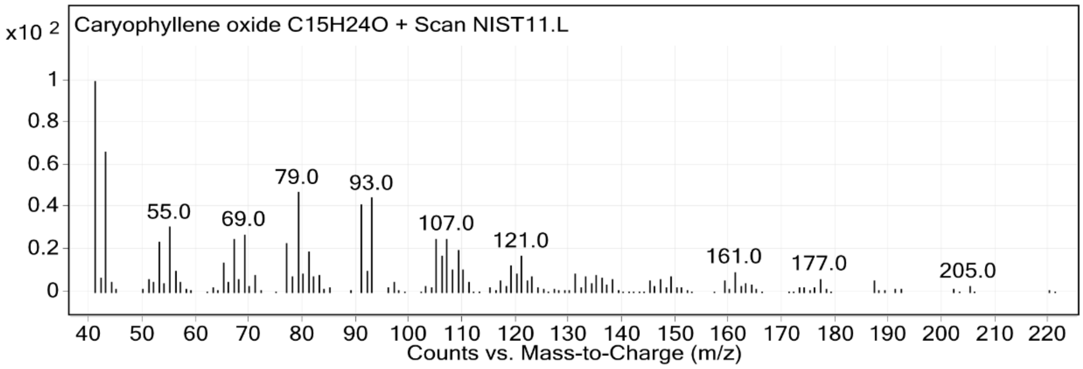

C **Spectrum Structure**

Caryophyllene oxide

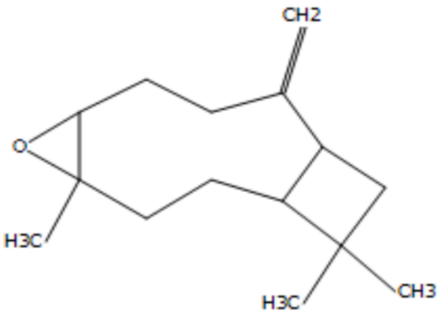

Figure S46 - Mass spectrum of Peak 45 from the TIC chromatogram, assigned to **Caryophyllene oxide**, for the second time. (A) Experimental mass spectrum extracted from the chromatographic analysis. (B) Reference mass spectrum from the NIST11 library used for identification. (C) Chemical structure of the identified compound.

FIGURE S47

A

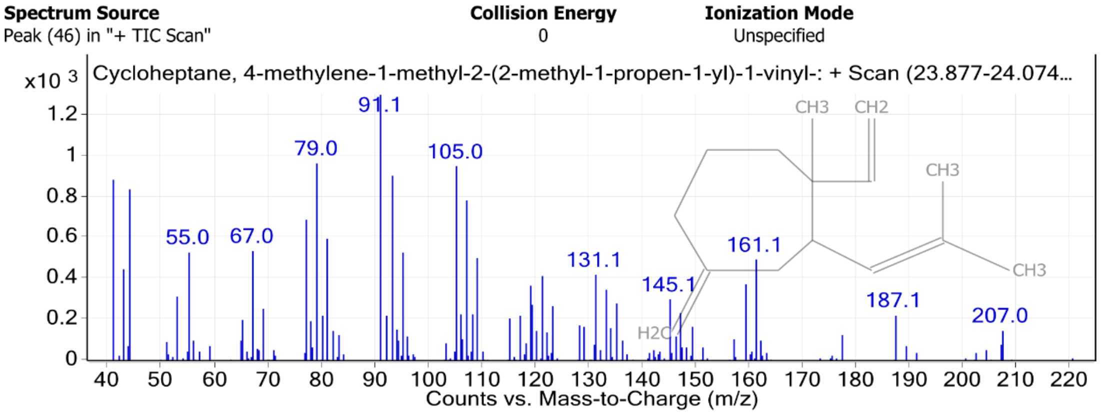

B

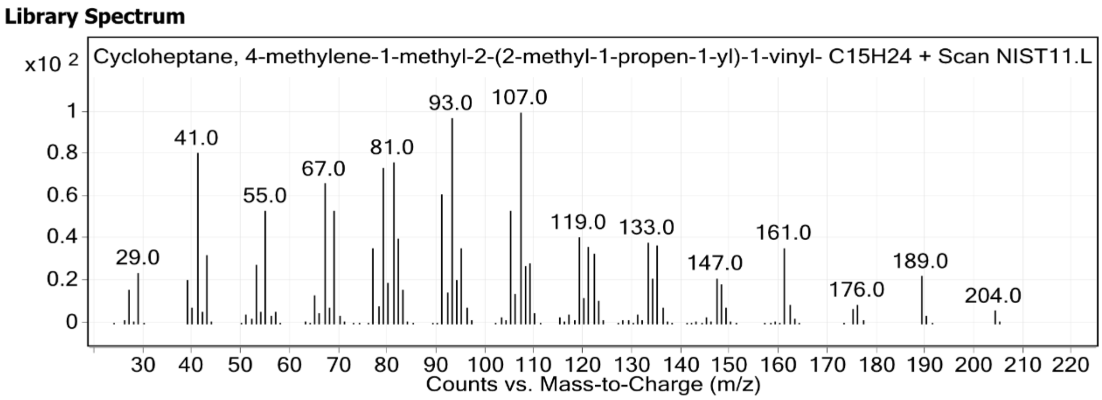

C

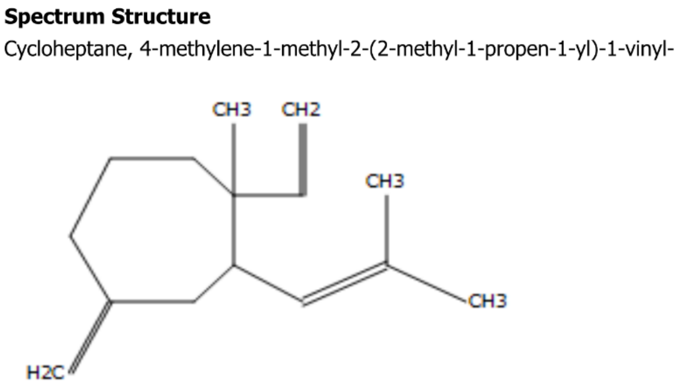

Figure S47 - Mass spectrum of Peak 46 from the TIC chromatogram, assigned to **Cycloheptane,4-methyllene-1-methyl-2-(2-methyl-1-propen-1yl)-1-vinyl-.** (A) Experimental mass spectrum extracted from the chromatographic analysis. (B) Reference mass spectrum from the NIST11 library used for identification. (C) Chemical structure of the identified compound.

FIGURE S48

A

**Spectrum Source**	**Collision Energy**	**Ionization Mode**
Peak (47) in "+ TIC Scan"	0	Unspecified

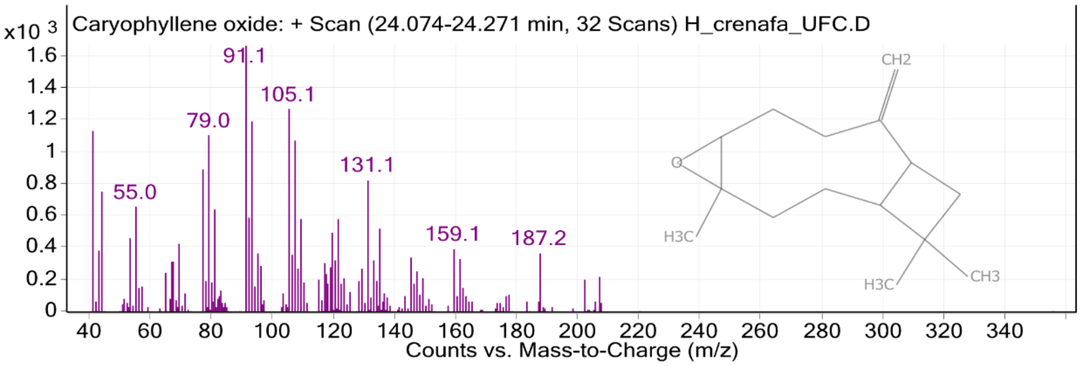

B **Library Spectrum**

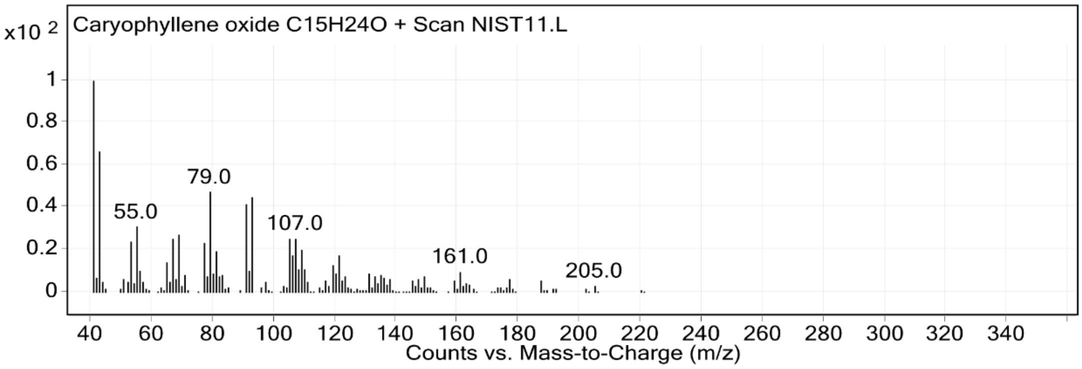

C

Spectrum Structure

Caryophyllene oxide

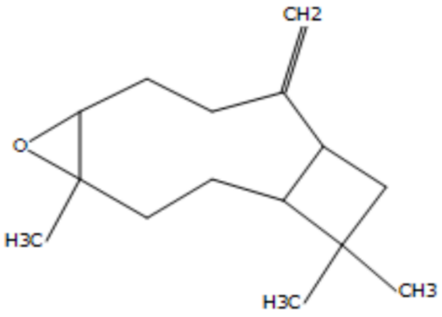

Figure S48 - Mass spectrum of Peak 47 from the TIC chromatogram, assigned to **Caryophyllene oxide**, for the second time. (A) Experimental mass spectrum extracted from the chromatographic analysis. (B) Reference mass spectrum from the NIST11 library used for identification. (C) Chemical structure of the identified compound.

TABLE S2

Binding Site	Compounds	Segments	Amino Acids	Interaction/Distance	Affinity (kcal.mol^{-1})
	Camphor	S4I, S6I, S6II	Leu^{517}, Asn^{632}, Ala^{636}, Ile^{1028}, Phe^{1032}	Hydrophobic, Alkyl/Pi-Alkyl (4–5 Å), H-Bond (2–3 Å)	-5.7
	β-Caryophyllene	S4I, S6II	Leu^{517}, Leu^{521}, Phe^{1023}, Phe^{1024}, Phe^{1032}	Hydrophobic, Alkyl/Pi-Alkyl (4–5 Å)	-7.9
**Site 1**	Caryophyllene Oxide	S4I, S6I, S6II	Leu^{517}, Leu^{521}, Ile^{900}, Phe^{1023}, Phe^{1024}, Ile^{1028}, Phe^{1032}	Hydrophobic, Alkyl/Pi-Alkyl (4–5 Å)	-7.8
	Cineole	S4I, S6I, S6II	Leu^{517}, Asn^{632}, Ala^{636}, Ile^{1028}, Phe^{1032}	Hydrophobic, Alkyl/Pi-Alkyl (4–5 Å)	-5.6
	Aromadendrene	S4I, S6I, S6II	Leu^{517}, Asn^{632}, Ala^{636}, Phe^{1032}	Hydrophobic, Alkyl/Pi-Alkyl (4–5 Å), Pi-Sigma (3–4 Å)	-7.4
	Camphor	S4IV, S6I, S6IV	Leu^{165}, Ile^{168}, Ala^{169}, Val^{172}, Val^{298}, Val^{615}	Hydrophobic, Alkyl/Pi-Alkyl (4–5 Å)	-5.6
	β-Caryophyllene	S4IV, S6I, S6IV	Leu^{165}, Ile^{168}, Ala^{169}, Val^{172}, Val^{298}, Leu^{295}, Val^{615}, Tyr^{619}, Leu^{622}	Hydrophobic, Alkyl/Pi-Alkyl (4–5 Å)	-7.3
**Site 2**	Caryophyllene Oxide	S4IV, S6I, S6IV	Leu^{165}, Val^{172}, Val^{298}, Leu^{295}, Val^{615}, Tyr^{619}, Leu^{622}	Hydrophobic, Alkyl/Pi-Alkyl (4–5 Å)	-7.3
	Cineole	S4IV, S6I, S6IV	Leu^{165}, Ile^{168}, Ala^{169}, Val^{172}, Val^{298}, Val^{615}, Tyr^{619}, Leu^{622}	Hydrophobic, Alkyl/Pi-Alkyl (4–5 Å)	-5.5
	Aromadendrene	S4IV, S6IV	Leu^{165}, Ile^{168}, Ala^{169}, Val^{172}, Val^{298}, Leu^{295}	Hydrophobic, Alkyl/Pi-Alkyl (4–5 Å), Pi-Sigma (3–4 Å)	-7.6
	Camphor	S6III, S6IV	Leu^{295}, Leu^{299}, Ile^{134}1, Ile^{1345}, Phe^{1345}	Hydrophobic, Alkyl/Pi-Alkyl (4–5 Å), Pi-Sigma (3–4 Å)	-5.6
	β-Caryophyllene	S6II, S6III, S6IV	Leu^{299}, Phe^{1029}, Val^{1030}, Ile^{1341}, Ile^{1342}, Phe^{1345}	Hydrophobic, Alkyl/Pi-Alkyl (4–5 Å), Pi-Sigma (3–4 Å)	-7.3
**Site 3**	Caryophyllene Oxide	S6II, S6III, S6IV	Leu^{299}, Phe ^{1029}, Val ^{1030}, Ile ^{1341}, Ile ^{1342}, Phe ^{1345}	Hydrophobic, Alkyl/Pi-Alkyl (4–5 Å), Pi-Sigma (3–4 Å)	-7.3
	Cineole	S6III, S6IV	Leu^{295}, Leu^{299}, Ile^{1341}, Ile^{1345}, Phe^{1345}	Hydrophobic, Alkyl/Pi-Alkyl (4–5 Å), Pi-Sigma (3–4 Å)	-5.5
	Aromadendrene	S6II, S6III, S6IV	Leu^{295}, Leu^{299}, Phe^{292}, Phe^{1029}, Ile^{1341}, Ile^{1342}, Phe^{1345}	Hydrophobic, Alkyl/Pi-Alkyl (4–5 Å)	-7.6
	β-Caryophyllene	S6II, S4II, S6III	Ile^{894}, Ile^{897}, Met^{1025}, Phe^{1029}, Val^{901}, Met^{1335}, Phe^{1339}	Hydrophobic, Alkyl/Pi-Alkyl (4–5 Å)	-7.4
**Site 4**	Caryophyllene Oxide	S6II, S4II, S6III	Ile^{894}, Ile^{897}, Ile^{1342}, Met^{1025}, Phe^{1029}, Val^{901}, Met^{1335}, Phe^{1339}	Hydrophobic, Alkyl/Pi-Alkyl (4–5 Å)	-7.2
	Cineole	S6II, S4II	Ile^{894}, Ile^{897}, Met^{1025}, Phe^{1029}, Val^{901}	Hydrophobic, Alkyl/Pi-Alkyl (4–5 Å)	-5.3
	Aromadendrene	S6II, S4II, S6III	Ile^{894}, Ile^{897}, Ile^{1028}, Ile^{1342}, Ala^{1338}, Met^{1025}, Met^{1335}, Phe^{1029}, Phe^{1339}, Val^{901}	Hydrophobic, Alkyl/Pi-Alkyl (4–5 Å)	-7.3

Binding Site	Compounds	Segments	Amino Acids	Interaction/Distance	Affinity (kcal.mol^{-1})
**Site 5**	β-Caryophyllene	S6I, S6IV	Phe^{306}, Arg^{310}, Ala^{627}, Val^{630}	Hydrophobic, Alkyl/Pi-Alkyl (4–5 Å)	-6.9
	Caryophyllene Oxide	S6I, S6IV	Phe^{306}, Ala^{627}	Hydrophobic, Alkyl/Pi-Alkyl (4–5 Å)	-6.8
	Aromadendrene	S6I, S6IV	Phe^{306}, Arg^{310}, Ala^{627}, Val^{630}	Hydrophobic, Alkyl/Pi-Alkyl (4–5 Å)	-6.9

Molecular docking interactions of selected compounds with the target protein across five predicted binding sites. Each row details a ligand's interaction profile at a specific binding site, including involved transmembrane segments, amino acid residues, interaction types and distances, and estimated binding affinities (in kcal.mol^{-1}). Interactions include hydrophobic contacts, alkyl and π-alkyl stacking, hydrogen bonds, and π-sigma interactions, with typical interaction distances in angstrom (Å).

TABLE S3

In Silico ADMET and Toxicological Profiling of Compounds

Category	Parameter	Camphor	Caryophyllene	Caryophyllene Oxide	Cineole	Aromadendrene
Physicochemical	MW	152.12	204.19	220.18	154.14	204.19
Physicochemical	LogP	2.324	5.341	4.536	2.582	4.585
Physicochemical	TPSA	17.07	0	12.53	9.23	0
Physicochemical	LogD	2.179	4.756	4.183	2.787	4.389
Absorption	GIA	High	High	High	Low	High
Absorption	Caco2	1.485	1.505	1.501	1.404	1.493
Absorption	Pg-sub	Yes	No	No	No	No
Absorption	PgI and II inhibitors	No	No	No	No	No
Distribution	VDss	0.331	0.652	0.586	0.491	0.753
Distribution	PPB	57%	97%	96%	93%	98%
Excretion	Total Clearance	0.701	0.1	0.17	0.352	0.04
Excretion	Renal OCT2	No	No	No	No	No
Metabolism	CYP1A2	No	No	No	No	No
Metabolism	CYP2C19	No	No	No	No	Yes
Metabolism	CYP2C9	No	No	Yes	No	No
Metabolism	CYP2D6	No	No	Yes	No	No
Metabolism	CYP3A4	No	No	Yes	No	No
Metabolism	CYP2D6 Substrate	No	No	No	No	No
Metabolism	CYP3A4 Substrate	No	No	No	No	No
Toxicity	AMES	0.025	0.004	0.014	0.01	0.011
Toxicity	Hepatotoxicity	0.218	0.157	0.281	0.448	0.256
Toxicity	DILI	0.041	0.131	0.397	0.046	0.243
Toxicity	Nephrotoxicity	0.124	0.273	0.208	0.138	0.057
Toxicity	Cardiotoxicity	No	No	No	No	No
Toxicity	Neurotoxicity	0.607	0.678	0.618	0.551	0.618
Toxicity	Hematotoxicity	0.295	0.419	0.416	0.267	0.17

Physicochemical properties, pharmacokinetics (absorption, distribution, metabolism, and excretion), and toxicity predictions for the five compounds (Camphor, Caryophyllene, Caryophellene Oxide, Cineole and Aromadendrene). Data were obtained using various *in silico* tools, and include parameters relevant to drug-likeness, bioavailability, metabolic interactions (CYP450), and potential toxicological effects.
